# Effectiveness and efficiency of an 11-week exercise intervention for patients with hip or knee osteoarthritis: a protocol for a controlled study in the context of health services research

**DOI:** 10.1186/s12889-016-3030-0

**Published:** 2016-04-30

**Authors:** Inga Krauss, Gerhard Mueller, Georg Haupt, Benjamin Steinhilber, Pia Janssen, Nicola Jentner, Peter Martus

**Affiliations:** Medical Clinic, Department of Sports Medicine, University Hospital, Hoppe-Seyler-Str. 6, 72076 Tuebingen, Germany; Allgemeine Ortskrankenkasse AOK, Baden-Wuerttemberg, Germany; Institute for Clinical Epidemiology and Applied Biometry, University Hospital, Tuebingen, Germany; Institute of Occupational and Social Medicine and Health Services Research, University Hospital, Tuebingen, Germany

**Keywords:** Hip osteoarthritis, Knee osteoarthritis, Group training, Home exercise, Propensity score matching, Health care research, WOMAC, Long-term effects, Cost-analysis, Safety

## Abstract

**Background:**

Osteoarthritis is the most common reason for pain in older adults, and the individual and economic burden of this disease is immense. The chronic character of osteoarthritis requires a long-term therapeutic treatment. In this regard life-style interventions such as physical exercises that can be carried out by the patient himself are recommended as first line treatment. There is evidence for the short-term benefit of exercise therapy in terms of pain reduction and physical functioning. Nonetheless research agendas highlight the need for multifaceted interventions that incorporate exercise strategies into patient care. Studies should be conducted with appropriate sample sizes and should allow statements on long-term effects as well as cost-utility and safety. These open questions are under the scope of this study.

**Methods/design:**

This is a controlled study in the context of health services research. The study population consists of *n* = 1400 subjects with hip or knee osteoarthritis. The intervention group will be recruited from participants of a country-wide health insurance offer for people with hip or knee osteoarthritis. Potential participants for the control group (ratio 10:1 (control vs. intervention) will be filtered out from the insurance data base according to pre-defined matching criteria and asked by letter for their participation. The final statistical twins from the responders (1:1) will be determined via propensity score matching. The progressive training intervention comprises 8 supervised group sessions, supplemented by home exercises (2/week over 11 weeks). Exercises include mobilization, strengthening and training of postural control. Primary outcomes are pain and function measured with the WOMAC Index immediately after the intervention period. Among other things, health related quality of life, self-efficacy, cost utility and safety will be evaluated as secondary outcomes. Participants will be followed up 6, 12 and 24 month after baseline.

**Discussion:**

Results of this trial will document the effects of clinical as well as economic outcomes in a regular health care setting on the basis of a large sample size. As such, results of this trial might have great impact on future implementations of group- and home-based exercises in hip or knee osteoarthritis.

**Trail registration:**

German Clinical Trial Register DRKS00009251. Registered 10 September 2015.

**Electronic supplementary material:**

The online version of this article (doi:10.1186/s12889-016-3030-0) contains supplementary material, which is available to authorized users.

## Background

### Epidemiology

Osteoarthritis (OA) is the most common reason for pain in older adults. OA is further associated with joint stiffness, crepitus, occasional effusion, variable degrees of inflammation, functional impairment, worsening of the general health status and a decrease in health related quality of life [1–3]. Several intrinsic and extrinsic risk factors are related to OA such as age (older), overweight, gender (women), genetic factors, occupational joint loads etc. In the German health reporting system 2010, approximately 27 % of the women and 18 % of the men reported a previous OA diagnosis [2]. The true value may even be higher as not every person suffering from typical OA symptoms will consult a doctor [2]. The knee and hip joints are the most commonly affected weight bearing joints. According to disease related symptoms and its prevalence, OA has a relevant impact on the individual person and the need for health services and consequently increases direct and indirect health care costs [2]. Cumulative cost of absence from work, medical costs and community and social services in Europe is currently estimated at 0.5 % of gross national product [4]. These costs will further increase in due consideration of the demographic change with a growing number of older adults and the increasing incidence of obesity as a relevant risk factor for knee OA [1, 5]. Research priorities should therefore be placed on the development and evaluation of effective and cost-efficient treatment strategies to counteract the aforementioned increase of personal as well as economic burden of OA.

### Treatment of lower limb osteoarthritis

To date joint replacement may be the only option for severe symptoms in hip or knee OA. In earlier stages, conservative therapeutic interventions are important to reduce pain and increase function and health-related quality of life [6]. Pharmacological therapies have significant toxicities limiting their permanent use in chronic diseases [4]. Non-pharmaceutical interventions such as physical exercise programs are therefore important therapeutic options. Treatment effects of these programs are similar to simple analgesics and oral non-steroidal anti-inflammatory drugs. However reports on known side-effects such as pain increase or falls are rare, thus exercise appears to be a safe intervention [7–9]. It is therefore hardly surprising that various international guidelines, expert committees and health institutions recommend a regular exercise regimen as a first line treatment for OA. Regimes should account for strengthening and/or low impact aerobic exercises. Further recommendations include aquatic exercises, Tai Chi, range of motion exercises and neuromuscular education [10–14].

Evidence is given for the short-term efficacy of joint specific exercise programs with respect to pain reduction and increase of function in subjects with knee and hip OA [8, 15, 16].

While the evidence suggests that exercise is an effective intervention further studies are needed:

- to test multi-faceted interventions that incorporate the exercise strategies into patient care [15].
- to assess effectiveness and safety of non-pharmacological management strategies [11].
- to assess the long-term effectiveness in terms of symptom relief, disease progression and exercise adherence [8, 11, 15].
- to assess efficiency of the intervention from an economic perspective [11].
- to assess which professional can best deliver the intervention [11].
- to conduct studies with appropriate sample sizes [11].

The above mentioned research topics will be addressed in this study on exercise intervention in subjects with hip and/or knee OA.

### Study purpose

This study will be conducted in the context of health services research. The exercise program is based on an intervention that was previously evaluated in a randomized placebo-controlled trial [17].

The primary aim of this study is to determine whether an 11-week progressive training program comprised of group sessions and home-based exercises decreases symptom related pain and increases physical function in subjects with hip and/or knee osteoarthritis in comparison to a matched control group in the short term. Effectiveness is quantified by the subscales *pain* and *physical function* of the WOMAC Index 3.1 NRS.

Secondary outcomes will be differentiated into a short-term perspective (3 months), mid-term perspective (6 months) and a long-term perspective (follow-up data 12 and 24 months after initiation of the intervention).

The secondary aims are to determine whether an 11-week progressive training program comprising group sessions and home-based exercises…

(I) …improves health-related quality of life and/or global rating of health.
(II) …decreases pain (mid-term, long-term only, as short-term is the primary study outcome)
(III) …improves physical function (mid-term, long-term only, as short-term is the primary study outcome)
(IV) …improves self-efficacy and physical activity levels
(V) …delays time to surgery
(VI) …is efficient with regard to personal and/or social costs

…in comparison to a matched control group (CO).

It is of further interest if group sessions and home-based exercises are feasible in a general health care setting in terms of exercise adherence and safety. Both will be monitored throughout the intervention period in the intervention group only.

## Methods/design

### Design (Fig. 1)

**Fig. 1:**
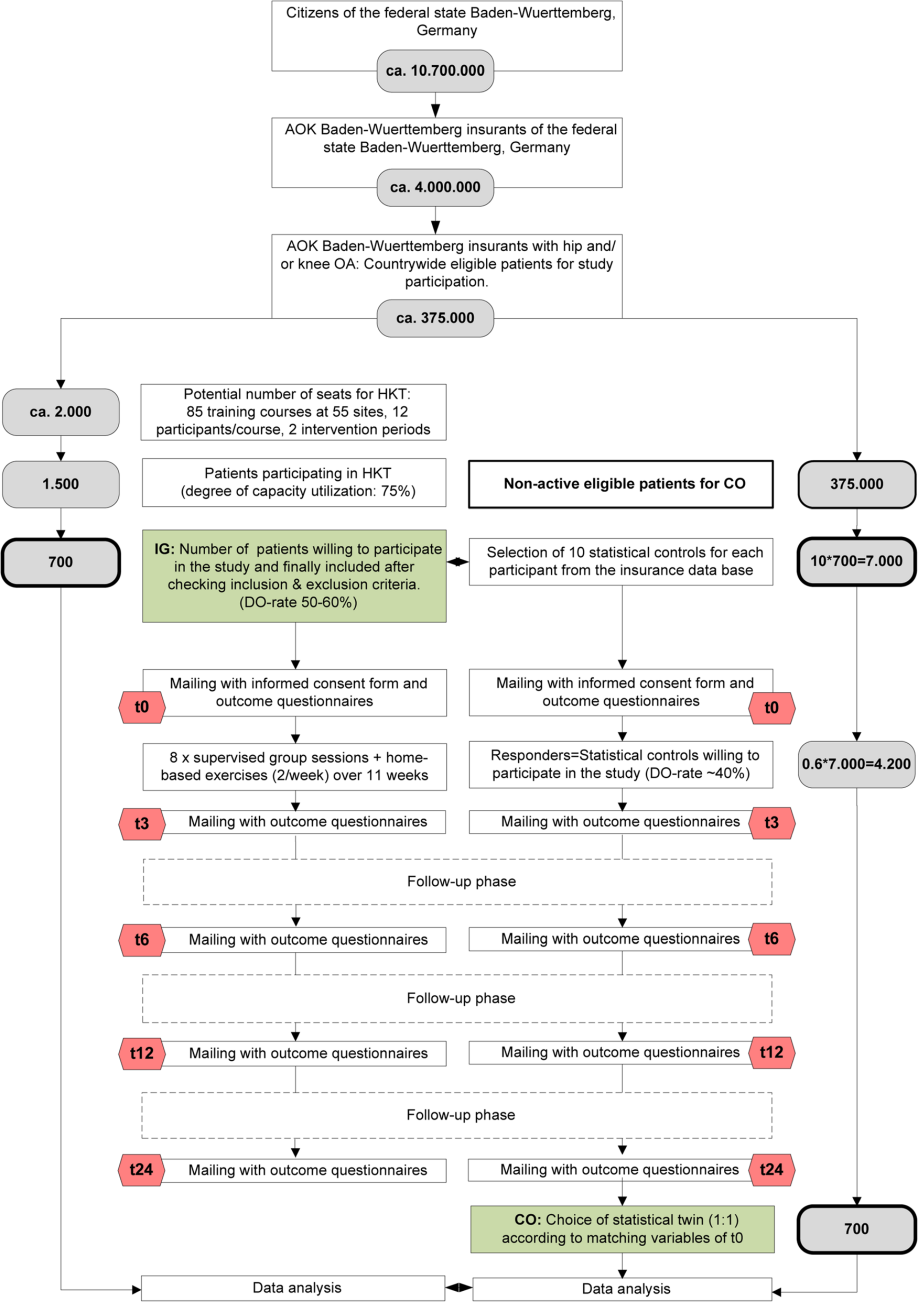
Study flow chart

This is a multi-center non-randomized controlled trial of an 11-week hip and knee training (HKT) with a 24 months follow-up period in the context of health services research. The exercise intervention comprises 8 supervised group sessions (1× / week for 8 weeks) and a home exercise program (2× / week for 11 weeks). All participants are insurance holders of a large statutory health insurance company covering approx. 40 % of the total population of the state Baden-Wuerttemberg. Participants of the intervention group (IG) will be compared to “statistical twins” (matched pairs) to investigate the effects of an exercise intervention on clinical and economic outcomes. Measurements will be taken at baseline (t0), 3 months (t3), 6 months (t6), 12 months (t12) and 24 months (t24). Economic data will further be evaluated retrospectively for two years prior to baseline.

- Recruitment of the intervention group (IG)
  - IG is recruited out of eligible participants of a countrywide health care offer of the above mentioned insurance company. Prerequisites for exercise participation are (1) membership in the insurance company, (2) referral from an orthopaedic surgeon or general practitioner because of complaints at the hip and/or knee. To be included into the accompanying study, exercise participants further have to confirm a self-reported lifetime prevalence of hip and/or knee OA. They have to affirm the following two questions according to the German Federal State health reporting system criteria [2]:
    - 1. “Have you ever visited a doctor because of complaints in the hip and/or knee joint?”
    - 2. “If yes, did a medical doctor ever ascertain OA or a degenerative disorder at your knee and/or hip joint?”
    - Further inclusion- and exclusion criteria for study participation are listed in Table 1.

**Table 1:** Inclusion and exclusion criteria for study participation (t0 = baseline)

Inclusion criteria (all criteria relevant for study eligibility)
• Insurance holder of the insurance company offering the exercise program since two or more years prior t0
• Referral from an orthopaedic surgeon or general practitioner
• Self-reported lifetime prevalence of hip and/or knee OA that was previously diagnosed by a medical practitioner • Physical and mental ability to participate in the interventional program and to answer self-administered questionnaires at t0
Exclusion criteria (one or more positive answers lead to non-eligibility)
• Significant established osteoporosis requiring treatment, previous spontaneous or low impact fracture prior t0
• Co-morbidities leading to major impairments in everyday life and representing contra-indications for physical activities at t0
• Artificial joint replacement at the knee and/or hip joint within the last 6 months prior t0
• Artificial joint replacement at the knee and/or hip joint with instable anchoring at t0
• Artificial joint replacement at the knee and/or hip joint with rradiologic signs of implant loosening at t0
• Artificial joint replacement at the knee and/or hip joint accompanied with acute joint inflammation at t0
• Current pain at rest or with activity due to artificial joint replacement at the knee and/or hip joint
• Luxation as an adverse event of artificial hip replacement prior t0
• Surgery at the lower extremity within the last 3 months prior t0
• Regular use of gait aids
• Self-reported acute illness at t0
• < 15 points^a^ on the WOMAC Index subscale pain (0–100) and < 15 points on the WOMAC Index subscale physical function (0–100) at t0
• Insufficient German language ability for self-administered questionnaires (IG) at t0
• Current employment in the health care insurance at t0

^a^Exclusion criteria is valid for the primary data analysis only. Low values indicate less pain and improved function: 0 points = no pain and maximum physical functioning respectively
  - The exercise intervention is accessible at 55 locations (study sites) of the country, offering 85 exercise courses each half year. The number of participants per exercise course is restricted to twelve persons. The recruitment period covers two course terms. Assuming twelve participants in each course, the intervention can be provided to approximately 2000 insurants. Eligible patients can participate on condition that their physicians prescribe the health care offer. Interested patients register for the intervention program at the location nearby and will be informed about the accompanying scientific evaluation of the exercise program. In this context, exclusion criteria are requested and only eligible patients will finally be allowed for the exercise intervention. Further details including the study information sheet for potential study participants, the informed consent and a check-list for all inclusion and exclusion criteria will be delivered by mail. This mail will be sent to each participant prior to the first training session. The mail further includes the self-administered questionnaires. In the context of all aforementioned stages it will be pointed out that study participation is not a presupposition for exercise participation. Eligibility of the patients to enter the exercise program and to participate in the study will finally be determined by checking the inclusion- and exclusion criteria for the exercise intervention 1st in the context of the first visit and 2nd by information recorded via the self-administered questionnaire at t0 (Table 1). Patients refraining from the study still are eligible to receive the exercise program.
- Feasibility of recruitment
  - We estimate that the offered exercise courses will be booked by 1500 patients (corresponding to 75 % occupancy rate). We further estimate a pre-study drop-out rate of 50–60 % due to lack of interest in study participation or exclusion according to study criteria. Thus approximately 700 patients will be allocated to the trial.
- Recruiting and selection of the control group (CO)
  - (1) In a first step the insurance ID of study participants will be forwarded to a specific department of the health insurance company. This department is responsible for the extraction of ten statistical controls for each study participant of the intervention group according to the procedure described in the statistical part of this manuscript. This pool of controls shall ensure the proper selection of one matched statistical twin for each participant in the second matching step according to number (5). The factor ten has already been proven successful in a previous study of the insurance company and was therefore adopted for the present study [18].
  - (2) The address and insurance number of the selected statistical controls according to (1) will be forwarded to another department of the company. This department will then forward study details (study information sheet for participants, informed consent form, questionnaires) by mail in order to ask if they are willing to participate in the control group of the study.
  - (3) Subsequently all duly completed questionnaires at t0 will be checked for in- and exclusion criteria which are part of the initial self-administered questionnaire (Table 1).
  - (4) Included study participants will then be recontacted to ask for follow-up data at t3, t6 and t12 (follow-up data will be gathered from all controls as the availability of economic data at t0 is temporally delayed for 12 month for organizational reasons).
  - (5) The final statistical twins (1:1 matching) will be selected using Propensity Score Matching (PSM). According to Austin, the propensity score can be explained as “the probability of treatment assignment conditional on observed baseline characteristics”. Matched-pairs are generated with nearest neighbor matching. “The nearest neighbor matching selects for matching to a given treated subject that untreated subject, whose propensity score is closest to that of the treated subject” [19]. Input variables for PSM are derived from the baseline data of the self-administered questionnaires (SAQ) as well as economic data from the insurance data base (IDB). The detailed matching procedure is described in the statistical part of this manuscript.
- Feasibility of recruitment
  - The selection of statistical twins is designed according to a successful procedure of a previous study of the insurance company. In this study on the efficacy of an exercise program for patients with back pain, a responder rate of the controls of 60 % could be achieved [18].

### Randomization process and allocation concealment

Not applicable.

### Blinding

The blinding of subjects to treatment is not possible as treatment exposure is evident.

The blinding of assessors of questionnaires is not applicable, as outcome measures are self-administered by the patients.

The blinding of the statistician who performs propensity score matching of controls is warranted with regard to any follow-up data (t3, t6, t12, t24) until all subjects of the intervention groups have their statistical twin.

The blinding for analysis of primary outcomes is warranted as group allocation is blinded for the statistician from t0 to t3.

The blinding for further outcomes is not warranted as interim reports will unblind results of the primary analysis.

### Interventions

All participants of the intervention group are requested to refrain from seeking other forms of treatment during the 11 week-intervention period from t0 to t3.

#### Hip and knee training (HKT)

The HKT is a comprehensive training program based on a previously evaluated 12-week exercise program specifically designed for patients with hip OA (17;20). The HKT was modified to allow its application for knee patients and reduced to 11 weeks with 8 supervised training sessions.

The HKT contains 8 weeks of group (1× / week, 60–90 min) and home based exercises (2× / week, 30–40 min) followed by 3 weeks of home-based exercises only (2× / week, 30–40 min). The 8-week group sessions include theoretical education and practical contents and enhance social contacts. The educational part improves the accomplishment of exercises. In this regard, participants are introduced into anatomical basics. They learn how to find and feel anatomical landmarks such as the spina iliaca anterior superior or the junction from the pelvis to the lower spine. Anatomical landmarks are important reference points to control a proper exercise execution. Participants are further introduced into training principles. They learn how to rate their subjective physical strain, how to deal with pain and how to report their home training sessions and potential adverse events in the training log. The theoretical contents are imparted in the supervised training sessions and can also be read up in the exercise manual that every participant receives. The supervised sessions enhance social contacts by having group-based introductions and feedback before and after the exercises, and by employing partner and group exercises. In the group sessions, subjects are introduced to the exercises they have to do at home.

Both, group and home training sessions include hip and knee exercises for motor learning and mobilization (MM), strength training (S) and exercises to improve postural control (PC). Elastic rubber bands, stability trainers (pads), exercise balls and exercise mats are used as training devices. Intensity and structure of the exercises follow a progressive concept. A more detailed description of group and home-based training modalities is given in the classification of the training phases and the exercise dosages of the different exercise tasks (Tables 2 and 3).

**Table 2:** Phased exercise program “Hip and knee training (HKT)”

Phase	Week	Home-based training	Group sessions Theory / Training	Objective
1	1-3	✔	60 min / 30 min 40 min / 50 min 30 min / 60 min	Mobilization, Motor learning
2	4-7	✔	30 min / 60 min - / 60 min - / 60 min - / 60 min	Balance training for postural control (static conditions), Strength endurance
3	8	✔	- / 60 min	Balance training for postural control (dynamic conditions), Endurance & maximum strength
	9-11	✔	- / -	Balance training for postural control (dynamic conditions), Endurance & maximum strength

**Table 3:** Exercise dosage

Objective	Sets/Repetitions(reps)/Intensity	Rest
Motor learning Mobilization Stretching	1 sets of 10 reps at <30 % MVC 1 sets of 30 reps at <30 % MVC 2 sets of 20 sec	A few sec <1 min A few sec
Strength endurance	2 sets of 20–25 reps at 30-40 % MVC 3 sets of 20–25 reps at 30-40 % MVC	1 min 1 min
Maximum strength	3 sets of 10–15 reps at 70 % MVC 4 sets of 10–15 reps at 70 % MVC	1-2 min 1–2 min
Postural control (static) Postural control (dynamic)	1 set of 6 reps of 15 sec 1 set of 6 reps of 15 sec	30-60 sec 30–60 sec

*MVC* Maximum voluntary contraction

The home training consists of 30 exercises for MM and 20 exercises for S. All subjects receive the exercise manual “Das Tübinger Hüftkonzept” (“The Tübingen Hip Concept”) [21]. Aside of its theoretical contents as outlined above, the exercise manual includes written instructions and pictures for the home training exercises. Patients with knee OA further receive a complementary leaflet where twelve MM hip exercises and ten S hip exercises are substituted by corresponding knee specific exercises. A brief description of the exercises for hip and knee patients (Additional file 1, Additional file 2) and an excerpt of the German-language exercise manual (Additional file 3) are depicted in the additional files.

**Fig. 2:**
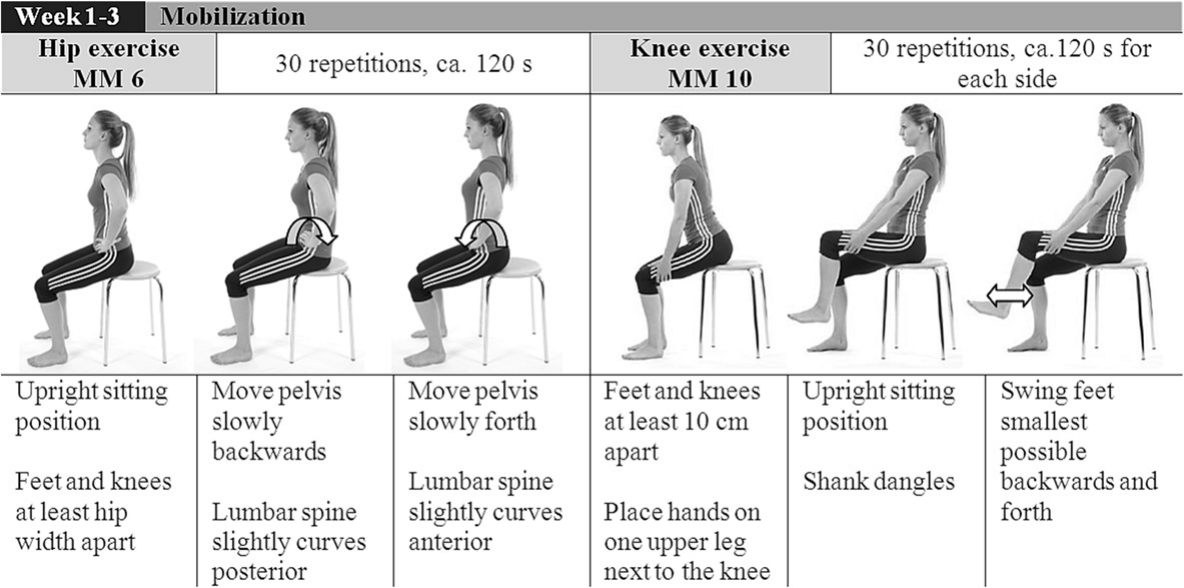
Example of an exercise for hip and knee motor learning and mobilization (MM)

Phase one (week 1–3) includes mobility exercises to increase flexibility of the pelvis, hip and knee joint and to enhance motor control to allow proper exercise execution. Examples of exercises for the hip and the knee joint are given in Fig. 2. Additional muscle stretching exercises address hip and knee flexors and extensors as well as hip adductors. Exercises in phase one are predominantly executed in a supine or seated position.

**Fig. 3:**
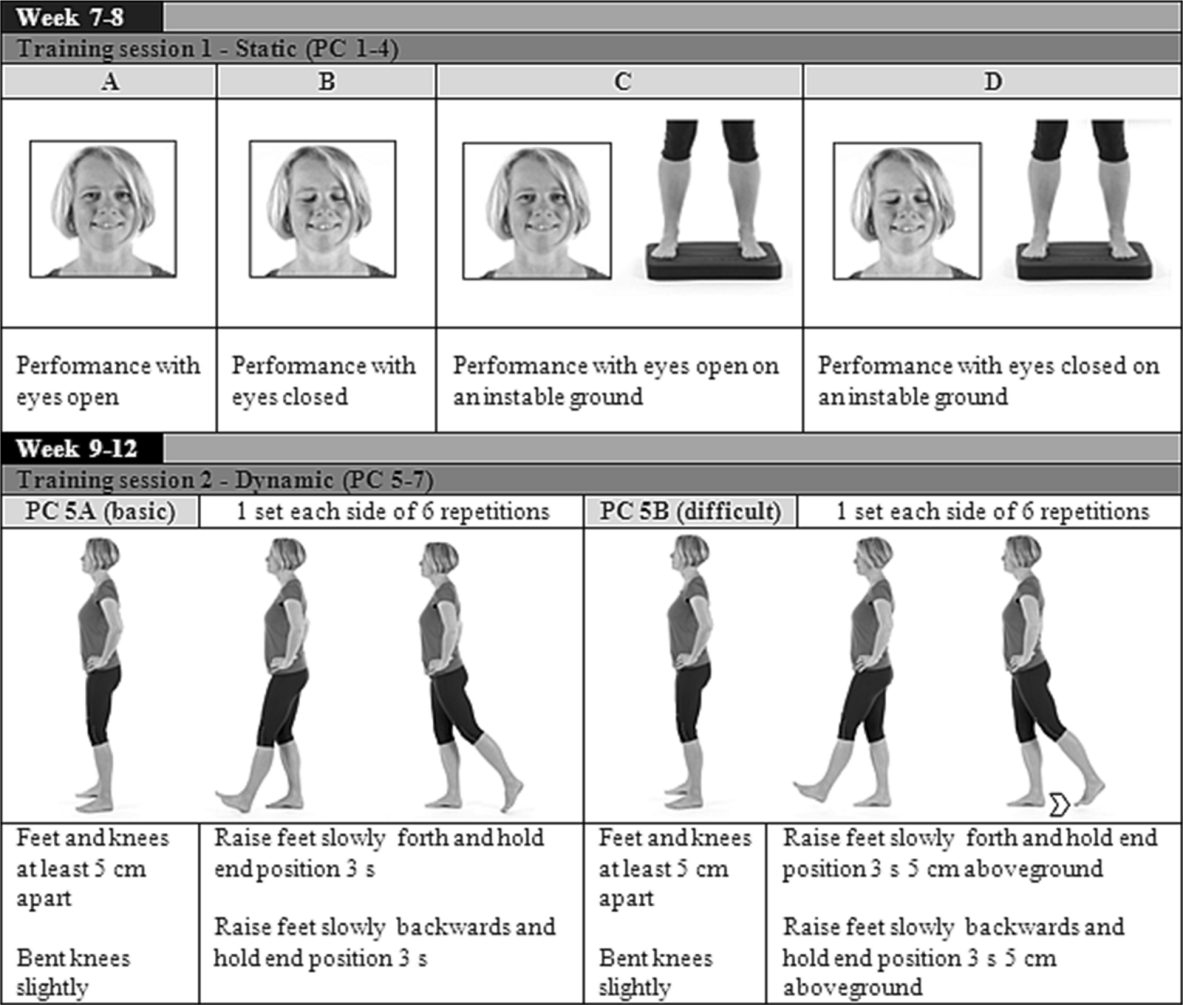
Example of static and dynamic exercises for postural control (PC)

**Fig. 4:**
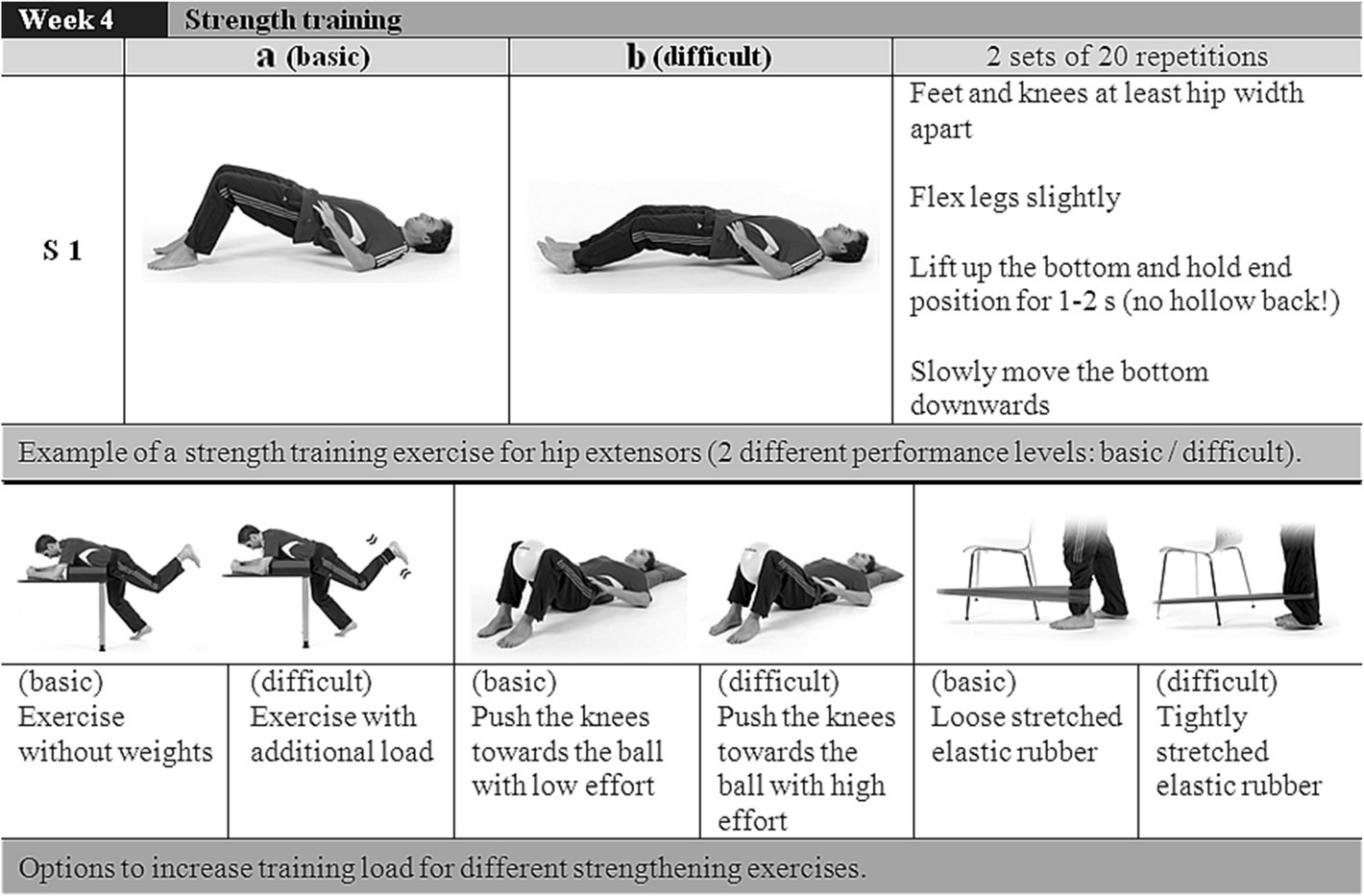
Examples for progressive strength training exercises (S)

The second phase (week 4–7) is divided into static balance exercises for postural control at the beginning of the training session followed by strengthening exercises. The difficulty of the static balance exercises can be adjusted according to the individual skills of the patients (difficulty level A–D, Fig. 3). Subsequent to the balance exercises the training program continues with open and closed kinetic chain exercises to improve muscular endurance. As displayed in Fig. 4, subjects have the possibility to match the strengthening exercises with the individual performance level by selecting exercise “a” or ”b”.

Demands of the exercises increase in phase three (week 8 to 11) to account for the progressive character of the exercise intervention. The balance exercises for postural control are expanded with three additional dynamic exercises. The difficulty of the dynamic balance training can be adjusted according to the individual skills of the patients (difficulty level PC-A or PC-B, Fig. 3). The strengthening exercises of this phase focus on the improvement of maximum strength.

The intensity of exercises in the different phases is controlled and quantified by the subjects’ subjective rating in dependence of the Borg perceived exertion scale [22]. While subjects should practice with low intensity during the first phase, the pre-settings of exercise intensity during the second and third phases are high. Subjects are advised to work out with a “hard” to “very hard” exercise intensity that would result in an exertion level of at least 14 on the Borg-Scale at the end of each set. The strength endurance phase (phase two) is characterized by subjects performing exercises with 2–3 series and 20 to 25 repetitions, corresponding to ~30–40 % maximum voluntary contraction (MVC) [23]. The hypertrophy phase includes exercises with 3–4 series and 10 to 15 repetitions, corresponding to ~70 % MVC [23]. The percentage MVC is estimated by the number of repetitions that can be conducted appropriately but with the subjective feeling of a hard workout. An overview of the exercise dosage is depicted in Table 3.

Group sessions will be conducted at the 55 country-wide training centres of the insurance company. They will be administered by health professionals of the company (i.e. physiotherapists, exercise scientists, trainers). All health professionals were additionally introduced into the hip and knee training in the context of a 2 days training program. They received a curriculum for the eight group sessions as well as detailed information on each particular exercise for the home training program [21], and they were introduced in how to teach participants accordingly. The educational program was held by the health professionals who designed the standardized intervention program.

#### Control group (CO)

CO receives general care. Participants of the control group may attend other offers of the health insurance company. Interventions are not restricted. However physical activity along the study period is monitored via self-administered questionnaires.

### Collection points (Fig. 5)

**Fig. 5:**
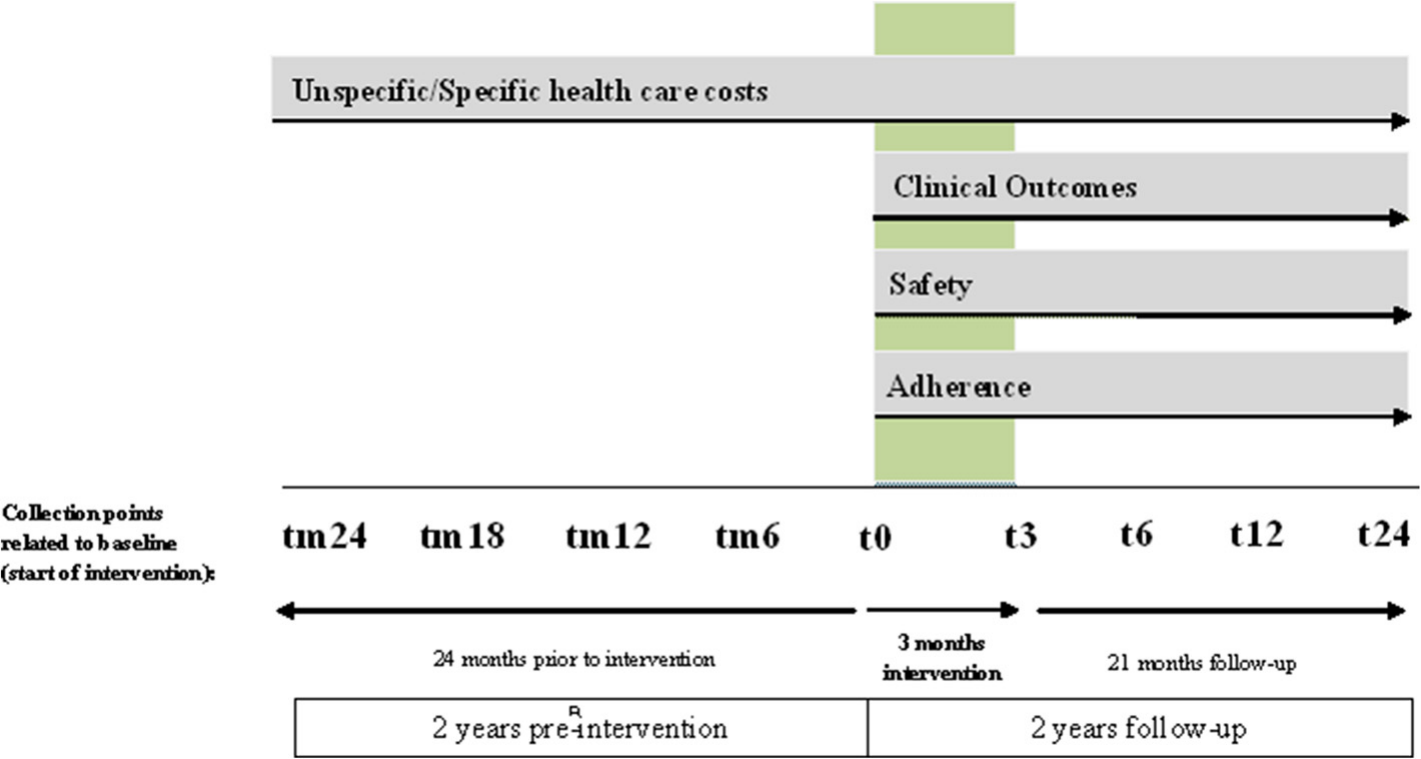
Collection points and outcome measures. Figure legend: t = collection point, m = minus. 0, 3, 6, 12, 24 = month of collection point related to baseline (t0)

Participants will be assessed at baseline (t0) and after (t3) the 11-week intervention period. Three follow-ups are conducted by mail 3 (t6), 6 (t12) and 21 (t24) months after the end of the intervention period. Economic evaluation will further compare data 6 (tm6), 12 (tm12), 18 (tm18) and 24 (tm24) months retrospectively (m = minus) prior to t0.

### Outcome measures (Table 4, Fig. 5)

**Table 4:** Outcome measures

Characteristics & confounders	Description and instrument	Data source	Sample	Collection points
Patient’s characteristics	Date of birth, gender, ethnicity, BMI (height, weight), site (s) of OA diagnosis, date of first OA diagnosis, labour situation (working, retired, unemployed, in rehabilitation status)	SAQ, IDB	IG, CO	t0, t3, t6, t12, t24
Primary outcome measure	Clinical Outcomes			
Pain	WOMAC Index 3.1 German (11-box NRS): subscale pain	SAQ	IG, CO	t0, t3
Function	WOMAC Index 3.1 German (11-box NRS): subscale physical functioning	SAQ	IG, CO	t0, t3
Secondary outcome measures	Clinical Outcomes		IG, CO	
Stiffness, disease specific impairment	WOMAC Index 3.1 German (11-box NRS): subscale stiffness, overall score	SAQ	IG, CO	t0, t3, t6, t12, t24
Pain, physical function	WOMAC Index 3.1 German (11-box NRS): subscale pain, subscale physical function	SAQ	IG, CO	t6, t12, t24
Health related quality of life	VR-12 incl. VR-6D utility index (4-week-time-slot)	SAQ	IG, CO	t0, t3, t6, t12, t24
General Self-Efficacy	General self-efficacy scale (GSE)	SAQ	IG, CO	t0, t3, t6, t12, t24
Response to exercise	Omeract-OARSI Set of Responder Criteria: composite score with minimum absolute and relative change levels for pain or pain & function	SAQ	IG, CO	t0, t3, t6, t12, t24
Physical activity status	Habitual physical and sports activity status	SAQ	IG, CO	t0, t3, t6, t12, t24
Time to surgery	Endpoint “elective joint replacement”	IDB	IG, CO	t0, t3, t6, t12, t24
	Patient satisfaction			
	Modified version of the ZUF-8 Questionnaire to assess patient satisfaction	SAQ	IG	t3
	Economic data			
Unspecific and specific (OA-related) health care costs	Out-patient costs, hospital costs, costs related to drugs, adjuvants and physical modalities, rehabilitation costs, sick-pay	IDB	IG, CO	tm24, tm18, tm12, tm6 t0, t3, t6, t12, t24
Unspecific/specific (OA related) periods of disability	Days of disability (overall and related to OA)	IDB	IG, CO	tm24, tm18, tm12, tm6 t0, t3, t6, t12, t24
Intervention related costs	Costs for human and physical resources/session	IDB	IG	t3, t6, t12, t24
	Adherence to exercise			
Training adherence	Summarized number of attended training sessions according to training log	SAQ	IG	t3
	Safety evaluation			
Adverse events and side-effects	Summarized number and details of adverse events and side-effects according to training log	SAQ	IG	t3

Data Source: Self-administered questionnaire via postal mailing (SAQ), insurance data base (IDB). Sample: Participants of the intervention group (IG), matched pairs control group (CO)

Annotation: Unless otherwise stated, measures are conducted at baseline and at all follow-ups.

The Western Ontario McMasters Universities Osteoarthritis Index (WOMAC® NRS 3.1 German Index) is a disease-specific instrument used to evaluate self-reported *pain*, *stiffness* and *functional impairment*. It is a valid, reliable and responsive score, easy to complete, simple to score and available in multiple language forms and scaling formats [24]. The scales will be transformed into values from zero (no limitation) to 100 (maximum limitation).

The OMERACT-OARSI Set of Responder Criteria is a composite score with minimum absolute and relative change levels for pain or pain & function. Input variables for pain and function will be derived from the WOMAC Subscales *pain* and *functional impairment* [25].

The VR-12 is an open access version of the SF-12 [26]. It comprises 8 different scales, four of them related to physical health (*physical functioning, role-physical, bodily pain, general health*), and four of them related to mental health (*vitality, social functioning, role-emotional, mental health*). The VR-6D is a utility measure derived from the VR-12. The 4 week time-slot will be used for analysis.

General self-efficacy will be measured with the German version of the General Self-Efficacy scale (GSE) with a four-point Likert scale (“not right” versus “definitely right”). Ten items are designed to tap this construct. Each item refers to successful coping and implies an internal-stable attribution of success. The scale ranges from 10 to 40 points with 40 points indicating maximum self-efficacy [27].

Habitual physical activity status will be quantified with regard to sports activities as well as physical activities in everyday life. Questions are similar to those being used in the German Health Update 2012 [28, 29].

Patient satisfaction (IG only, t3) will be quantified by participants of the IG with a four point Likert scale using a modified and shortened version of the ZUF-8 [30, 31]. Modifications were made in order to adapt the eight item instrument to the outpatient setting.

- Patient characteristics
  - Body Mass Index (BMI), site (s) of OA joint diagnosis, artificial joint replacement (hip or knee and time of event).
- Safety evaluation (IG only, t3)
  - Description of adverse events within the previous 11-week intervention period including frequency, intensity and duration of exercise related pain and possible reasons. Participants get a training log for home-based exercises where they are asked to report adherence as well as exercise related adverse events. In this way summarized data can be provided by the participants at the end of the intervention period by reviewing the training logs. Aside from this study-related safety report, patients are urged to contact their personal trainers or physicians in case of adverse events and side-effects.
- Adherence to exercise (IG only, t3)
  - Number of attended training sessions (group and home-based exercises). In case of omitted sessions: Reasons for non-compliance.
- Socio-demographic data (insurance data base)
  - The following socio-demographic data will be readout of the data base: date of birth, gender and labor situation (working, retired, unemployed, in rehabilitation status), complexity of work (from 1 = low to 4 = high), level of education (1 = no graduation to 4 = High School), highest level of educational attainment (from 1 = no qualification to 6 = doctoral degree), contractual form (permanent/fixed term contract, full time/part time).
- Economic data (insurance data base)
  - Patient-specific economic data comprise (1) unspecific and (2) specific osteoarthritis related health care costs for the diagnosis of hip and/or knee OA, (3) Specific and unspecific days of disability and (4) intervention related costs for the exercise program:
  - (1) Unspecific health care costs (overall costs): Sick-pay, hospital costs, out-patient costs, rehabilitation costs and costs related to drugs, physical modalities and adjuvants.
  - (2) Specific diagnosis (hip and knee OA) related health care costs: Sick-pay, hospital costs, out-patient costs, rehabilitation costs and costs related to disease related drugs, physical modalities and adjuvants.
  - (3) Specific (hip and knee OA related) and unspecific days of disability.
  - (4) Intervention related costs include human and physical resources that are required for the institutional exercise sessions. Intervention related costs will be added to the above mentioned diagnosis related costs for all participants of the intervention groups according to the number of their scheduled training session (8 units).

### Statistics

#### Sample size

Below are given details on the sample size estimation for primary clinical outcomes. However, aside from statistical power, a sufficiently large sample is necessary to obtain representative results for the general population and to allow sustainable statements on the effectiveness and efficiency of the intervention for secondary clinical as well as economic outcomes. We therefore plan to include all eligible patients willing to participate in the study and finally assume a number of n = 700 in each group. This number takes into consideration the degree of capacity utilization of hip and knee training courses as well as the number of subjects non eligible due to inclusion- and exclusion criteria as well as subjects refusing consent to participate in the study (Fig. 1).

The empirical basis of the sample size estimation for primary outcomes was retrieved from the paper by Krauss et al. [17]. In this randomized controlled trial (RCT), intra-individual differences of WOMAC subscale pain and WOMAC subscale physical function identically showed an effect size of about 0.5 between the intervention and the control group (pain, intervention: −8.5 ± 13.9, control: −1.3 ± 15.3, physical function, intervention: −8.4 ± 13.4, control: −2.1 ± 12.9).

Based on the previous results of the RCT and the so-called efficacy-effectiveness gap between RCTs and studies under real life conditions [32] we finally assume an effect size of ES = 0.3.

Due to the fact that we want to test the two endpoints (pain and physical function) simultaneously and without hierarchical order, we choose a level of significance of 0.025 (two-sided, Bonferroni correction) and a power of 0.90. This leads to a sample size of 278 subjects per group in the parallel group design without cluster effects (nquery release 7.0). The drop-out rate in the intervention group of the above mentioned RCT was 8 % [17]. As this value is quite low, we assume a somewhat higher number of non-finishers and estimate the rate to be 20 %. Thus 350 subjects should be allocated to each treatment arm.

However, in our study, cluster effects may appear for subjects within identical training groups: We expect 700 included subjects participating in 170 different training courses. Thus four participants of each training group will averagely be subjects of the study (whereas the rest of the group participants are no study subjects). With a rather high intra class correlation of 0.33 we obtain a variance inflation factor of 2 for the intervention. Therefore, with 700 subjects per study arm the aimed power should be reached.

We refrain from analyzing our data using a matched-pair design as propensity score matching does not guarantee that individual pairs will be well-matched on the full set of covariates, only that groups of individuals with similar propensity scores will have similar covariate distributions [33]. Despite this fact, propensity score matching including among other variables baseline WOMAC scores as matching factors may reduce variance of outcome measures and therefore probably increase the power of the study further if the propensity score is included as covariate in the statistical evaluation model. However, it is difficult to quantify this effect and thus we conservatively calculated the sample size obtained for the parallel group design with cluster effect as described in the preceding paragraph.

#### Matching procedures and statistical analysis of clinical endpoints

The matching procedure for the statistical twins of the control group (CO) will be conducted as followed: ten statistical controls for each participant of the intervention group will be selected from the insurance data base according to the matching criteria displayed in Table 5. If it is not possible to select ten matches for a subject of the intervention group according to the criteria and tolerances displayed in Table 5, the tolerance will be extended in an iterative manner (steps of 100 Euro or 1 day respectively) until ten controls can be extracted from the data base.

**Table 5:** Matching criteria to extract 10 controls for each participant of the intervention

Criteria	Tolerance
Co-morbidity: Quantity of hierarchical ordered morbidity groups in the previous year	0
Osteoarthritis of the hip or knee joint in the previous year: yes/no	0
Participation in a special general practitioner care program (“Hausarztzentrierte Versorgung”): yes/no	0
Routine data: Age (years), gender, type of insurance (compulsorily insured, family insured, pensioner, unemployed), complexity of work (from 1 = low to 4 = high), level of education (1 = no graduation to 4 = High School), highest level of educational attainment (from 1 = no qualification to 6 = doctoral degree), contractual form (permanent/fixed term contract, full time/part time)	0
Joint replacement in the last 24 month prior t0 at the hip and/or knee joint(s): yes/no	0
Sum of unspecific health care costs (overall costs) in the last 24 month prior t0: Sick-pay, hospital costs, out-patient costs, costs related to periods of disability and costs related to drugs, physical modalities and adjuvants.	+/− 100 EUR
Sum of unspecific health care costs (overall costs) in the last 6 month prior t0 (tm6): Sick-pay, hospital costs, out-patient costs, costs related to periods of disability and costs related to drugs, physical modalities and adjuvants.	+/− 100 EUR
Sum of specific diagnosis (hip/knee OA) related health care costs in the last 24 month prior t0: Sick-pay, hospital costs, out-patient costs, costs related to periods of disability and costs related to disease related drugs, physical modalities and adjuvants such as walkers, cranks or orthotics.	+/− 100 EUR
Sum of specific diagnosis (hip/knee OA) related health care costs in the last 6 month prior t0 (tm6): Sick-pay, hospital costs, out-patient costs, costs related to periods of disability and costs related to disease related drugs, physical modalities and adjuvants such as walkers, cranks or orthotics.	+/− 100 EUR
Disability days in the last 24 month prior t0 (tm24)	+/− 1 Day
Disability days in the last 6 month prior t0 (tm6)	+/− 1 Day
Specific disability days (hip/knee OA) days in the last 24 month prior t0 (tm24)	+/− 1 Day
Specific disability days (hip/knee OA) in the last 6 month prior t0 (tm6)	+/− 1 Day

Legend: a tolerance of “0” is equal to complete agreement, i.e. for age in years. Matching criteria are derived from the insurance data base

The matching process for the identification of statistical controls (CO) for each subject of the intervention group will be permuted in blocks: The matching procedure will be conducted quarterly until the number of needed subjects is reached.

Selected subjects will be asked for study participation (see section recruiting). The final statistical twins for each participant (1:1 matching) will be selected from all eligible responders. As a primary criterion, twins must be identical with regard to site(s) of OA (hip, knee or both). The following independent variables of the self-administered questionnaires (SAQ) and insurance data base (IDB) will then be used as input variables for the logistic regression of the PSM:

- Socio-demographic data at t0: age and gender (SAQ)
- Co-morbidity: Quantity of hierarchical ordered morbidity groups in the previous year (IDB)
- Osteoarthritis of the hip or knee in the previous year (IDB)
- WOMAC Index subscale pain at t0 (SAQ).
- WOMAC Index subscale physical functioning at t0 (SAQ).
- Quality Adjusted Life Years at t0 via VR-6D (SAQ).
- Body mass index at t0 (SAQ).
- Joint replacement hip and/or knee joint(s) at t0 (SAQ).
- General Self-Efficacy scale GSE at t0 (SAQ)
- Habitual physical and sports activity status at t0
- Participation in health activity programs (i.e. walking courses) at t0

If there are any cost/disability day categories, in which the standard mean differences after matching are above 0.05, a new matching will be conducted with these cost/disability day categories as matching variables in addition. No follow-up data will be analyzed prior to the final definition of the statistical twin of the CO for a given IG patient.

The finally defined controls and cases will be included into a linear model with the propensity score as covariate. Additionally, we will use models for longitudinal data using each measurement point (instead of only t0 and t3 for the primary analysis). The level of significance will be 0.025 for primary endpoints. Secondary endpoints will be analyzed analogously without claiming confirmatory interpretation of *p*-values.

Descriptive analysis will include absolute and percentage frequencies for categorical variables, means, medians, standard deviations, quartiles and ranges for quantitative variables and medians, quartiles and ranges for ordinal variables. For the main results, two-sided 95 % confidence limits will be given additionally to significance tests. For percentages exact confidence interval for proportions based on the binomial distribution will be given.

Exploratory, prognostic factors will be analyzed using multiple regression models (linear regression) to identify potential responders to the training.

Correlation analyses will use Pearson product moment correlation coefficient (normally distributed variables) and Spearman correlation (non-normally distributed variables).

The primary analysis will be done on the full set of patients recruited in consideration of the exclusion criteria “limited pain and impairment in physical function” (WOMAC Index subscales pain and physical function with values below 15) except for patients refusing consent during the study and obviously wrongly diagnosed patients. A secondary analysis will be done using the full set of eligible and included patients irrespective of their limitations in pain and physical function at baseline (t0). This secondary analysis will only claim confirmatory interpretation of p-values if the primary analysis is able to detect group differences in a statistical manner. Otherwise the secondary analysis will be done in an explorative manner.

Missing values will be imputed using multiple imputation approaches, complete case and last observation forward analysis will be performed as a sensitivity analysis. No interim analysis, except for administrative purposes will be performed. All statistical analysis will be done using the software SPSS and R in the newest release.

#### Economic evaluation

Cost-efficiency of the intervention will be quantified on the expanded perspective of the payer. If a dominant strategy doesn’t exist (i.e.: lower costs and higher health related effects in the intervention group), the costs will be related to the effects. The costs (including the supplementary costs of the pilot offer for participants) will be related to the differences between the groups (Double-Difference Method, two years pre and two years post intervention) in Quality Adjusted Life Years (QALYs, equation 1) and health related effects (WOMAC Index: equation 2). The costs of the intervention will further be related to the differences in costs (Double-Difference Method of the health care costs (unspecific health care costs (overall costs), specific health care costs and the costs for days of disability (human capital approach): equation 3). The time to surgery will be estimated using the Kaplan-Meier method. For the economic evaluation all participants have to be members of the insurance the period from 24 months before baseline (tm24) up to 24 month after baseline (t24).

Equation 1: Cost-Utility Analysis (CUA) = Incremental Cost Utility Ratio (ICUR)

$$ ICUR\kern0.49em =\kern0.49em \frac{\varDelta\ \mathrm{Cost}}{\varDelta\ \mathrm{QALYs}} $$

Equation 2: Cost-Effectiveness Analysis (CEA) = Incremental Cost-Effectiveness Ratio

$$ ICER\kern0.49em =\kern0.49em \frac{\varDelta\ \mathrm{Cost}}{\varDelta\ \mathrm{Effect}} $$

Equation 3: Cost-Benefit Analysis (CBA) = Incremental Cost-Benefit-Ratio

$$ ICBR\kern0.49em =\kern0.49em \frac{\varDelta\ \mathrm{Cost}}{\varDelta\ \mathrm{Benefit}} $$

### Timelines

Ethical approval was obtained in August 2015. Training of exercise instructors was undertaken in August and September 2014 and July 2015. Recruitment of participants for the study started in September 2015 for the first intervention period (first patient in) and will be continued until March 2016 (last patient in). All participants are expected to have completed the intervention period in July 2016. All participants are expected to have completed the study by February 2018 (last patient out).

### Expert report

The study will be assessed by an independent organization (XCENDA, http://www.xcenda.de/index.php/willkommen.html).

## Discussion

Despite the strong evidence for the short-term effects of physical exercises on symptoms in knee and hip OA [8, 15, 16], many open questions remain with respect to (I) the incorporation of interventions into patient care in a regular health care setting, (II) long-term effects of the intervention as well as its impact on disease progression, (III) dose–response-relationship according to FITT principles (frequency, intensity, type and time of training) and (IV) knowledge on variables that act as confounders for treatment response. Open questions are also related to (V) the economic efficiency of exercise interventions in the treatment of hip and knee OA and to (VI) safety aspects of the intervention [8, 11, 15].

This study proceeds in a real health care setting and has the potential to answer questions in the aforementioned research areas.

(I) This study allows us to determine whether the efficacy of an exercise intervention proofed in a randomized controlled trial can be transferred into effectiveness into a real life situation with less standardized and controlled conditions. The intervention described in this study protocol has been derived from a previously tested exercise intervention in patients with hip OA [17, 20]. Yet some adaptations to the intervention had to be made to give consideration to the needs and organizational framework of the health care provider: the formerly 12-week intervention with one supervised session/week and two additional home-exercise sessions/week was reduced to 8 supervised sessions. Patients are instructed to perform the accompanying home-exercise sessions for at least 11 weeks according to the exercise manual every participant receives [21]. Aside from this change that is related to the frequency and duration of the exercise intervention, the population under consideration has been extended: the RCT was conducted on subjects with hip OA only. As prevalence for knee OA in comparison to hip OA is even higher, the program will be offered to patients with hip and/or knee OA. Subjects with knee OA will therefore substitute some of the hip-exercises with knee-specific exercises. On this note, subjects with knee OA receive an amendment for the exercise manual with knee-specific exercises. Results of this study are therefore very valuable as they allow us to verify whether an “evidence-based exercise intervention” can successfully be implemented into everyday routine or not.

(II) Our follow-up data will be gathered up to 2 years after the initiation of the supervised exercise intervention and will therefore give information on clinical and economic outcomes in the long-term.

(III) The intervention program of this study is predefined and we do not differentiate between different dosages. Nonetheless data on adherence to exercise can be related to effect size and may allow an interpretation on continuity of an exercise intervention in relation to response, whereby confounders for exercise adherence such as pain or dissatisfaction with the intervention etc. have to be considered as well. Another aspect that is related to exercise dosage is the possibility to compare results of this study to other interventions with different exercise frequencies, intensities, times and types of intervention (FITT). This comparison may therefore contribute to the knowledge gained with respect to dose–response relationships of exercise interventions in OA.

(IV) Aside from the comparison of different interventions in general, an individual perspective may gather new knowledge in terms of personal contextual factors that are related to intervention outcome. Identification of possible predictors of treatment responsiveness is therefore another important issue of this research. For example data of previous studies report a gender-specific response to exercise [34, 35]. Additional confounders may be related to baseline pain level, self-efficacy, socio-demographic data etc. In the context of this study, a responder analysis will be conducted according to the OMERACT-OARSI Set of responder criteria [25]. A sub-analysis comparing personal contextual factors of responders versus non-responders may allow a better understanding of relevant prerequisites for successful exercise participation and therefore facilitate individualized treatment recommendations in OA.

(V) Considering published reports on cost-effectiveness of non-pharmacologic, non-surgical interventions for hip and/or knee OA, Pinto and colleagues concluded in their review in 2012 that there is only limited evidence for the cost-effectiveness of conservative treatments for hip and knee OA. In this context they highlighted the need for more high-quality economic evaluations [36]. Since then studies have been published that indicate no cost-saving but cost-effectiveness of exercise interventions in knee or hip OA [37, 38]. In addition, an increasing number of study protocols include economic outcomes and underline the need for the evaluation of economic outcomes aside from clinical measures [39–41]. The aforementioned studies as well as the trial of this protocol will allow a better understanding of cost-utility measures in consideration of different exercise programs and populations under study.

(VI) Although non-pharmaceutical trials are not obligated by law to report on adverse events, safety should be evaluated in clinical trials. Knowledge on adverse events may allow a more precise determination of contra-indications for a given exercise setting and/or population group. Report of adverse events is also important as exercise interventions can initially result in a slight increase in pain [42]. Knowledge on potential short-term side-effects in the primary phase of the intervention period is important as it allows health care providers to inform patients about potential harms with no significant lasting effect on health outcomes. Another important safety aspect is related to frequency and severity of adverse events according to the mode of delivery: Reports on adverse events related to exercises may differ between group sessions and home-based exercises. Safety in the home setting may be hampered as exercises are not controlled by a health care professional and the environment may be less appropriate for the execution of exercises (i.e. tripping hazards are more likely in a home-based setting). Information on adverse events is requested in the self-administered questionnaires at the end of the intervention period. This study may therefore gain knowledge on safety aspects and subsequently allow recommendations to improve safety of supervised as well as independently undertaken exercises. However it has to be mentioned that a reporting bias-especially in the context of home-based exercises - cannot be ruled out, as no immediate adverse event reporting is implemented in this study.

There are further limitations to our study which are—from our perspective—mainly related to concomitant health care interventions, limited long—term follow—up data and the statistical power in terms of economic evaluations.

Referring to the first point it has to be mentioned that all participants of the study are allowed to take part in additional health care offers. This holds true for participants of the control group as well. They may also attend the hip and knee training under study within the follow-up phase. This cannot be prohibited as all insurance holders have the same rights to participate in health care services offered. The exclusive participation in the assigned intervention (supervised exercise versus matched pair control group) can therefore only be guaranteed for the first 3 month of the study. This timeframe is also relevant for the evaluation of the primary outcomes. To control for potential confounders related to additional health care interventions, all follow-up questionnaires ask for participation in any course or training offer.

Although follow-up data for two years will be gathered in the context of this trial, statements on the effect of the intervention in the long run such as time to surgery and disease specific health care costs may be hampered. If 24-months evaluations are positive in terms of study outcomes, a protocol amendment for a 5-year follow up may be considered.

This study has 80 % power for unfavorably high cluster effects, in realty probably more than 90 % power to detect clinical relevant changes in the primary outcomes of the trial. Aside from clinical outcomes, cost-utility measures are of special interest in this study. Yet economic outcomes are expected to have much smaller effect sizes. We therefore decided to include all eligible patients, willing to participate in the trial, to increase power for the economic evaluation.

In due consideration of the burden of OA from an individual as well as economic perspective, the importance to evaluate the effectiveness and cost-efficiency of conservative treatment strategies is obvious. This applies in particular for lifestyle interventions such as physical exercises as they can be carried out by the patient himself and are therefore of special importance in the treatment of chronic diseases such as OA. Results of this trial will document accurately the effects of exercises on clinical and economic outcomes as well as safety aspects in a health care setting on the basis of a large sample size. As such, results of this trial should provide externally valid exercise recommendations in hip or knee osteoarthritis.

### Data privacy, ethics approval and consent to participate

Data privacy is warranted by including different parties to guarantee pseudonymization of patient data derived from different data sources. Each party has a predefined right of access to personal and study data of the participants. Only one independent party has access to personal insurance data as well as study outcomes. All other parties only have access to personal data or study data. Data privacy was approved by the data protection officer of the insurance company.

All participants of the exercise program receive a postal mailing prior to the first training session. This mailing includes information on the aims and the content of the study as well as on data privacy: in case of study participation, consolidation, analysis and utilization of data is carried out with random names only. The mailing further includes the informed consent form and the outcome questionnaire. The selected subjects from the insurance data base for the control group receive a similar mailing. Interested persons of the intervention as well as control group are informed that they confirm their informed consent by returning the informed consent form for data privacy and the pseudomyzed outcome questionnaires by post. Patients are informed about the voluntariness of study participation at all times. Participants of the exercise program are further informed that study participation is not a requirement for participation in this health care offer.

Ethical approval has been obtained from the Ethics Committee of the University of Tuebingen (Vote number 421/2015BO1).

Study data are erased after the legal retention period according to privacy policy apart from data that were already analyzed and recorded as study report and/or publication.

## Additional files

Additional file 1: Description hip training. (PDF 97 kb) Additional file 2: Description knee training. (PDF 96 kb) Additional file 3: Excerpt of the German-language exercise manual. (PDF 2202 kb)

## Abbreviations

BMI: Body Mass Index
CBA: Cost-Benefit Analysis
CEA: Cost-Effectiveness Analysis
CEA: Incremental Cost-Effectiveness Ratio
CO: Control group
CUA: Cost-Utility Analysis
FITT: Frequency, Intensity, Time and Type (of exercise interventions)
GSE: General Self-Efficacy scale
HKT: Group-based hip and knee training in combination with a structured home-exercise program
ICUR: Incremental Cost Utility Ratio
IDB: Insurance data base
IG: Intervention group
MM: Exercises for motor learning/mobilization
OA: Osteoarthritis
PC: Exercises to improve postural control
PSM: Propensity score matching
QALY: Quality adjusted life years
S: Exercises for strength training
SAQ: Self-administerd questionnaire
VR-12: 8-scale questionnaire to measure health related quality of life
WOMAC: Western Ontario and McMaster Universities Osteoarthritis Index
