# Supplementary material for: Effectiveness and efficiency of an 11-week exercise intervention for patients with hip or knee osteoarthritis: a protocol for a controlled study in the context of health services research
Source: BMC Public Health. 2016 Apr 30;16:367. doi: 10.1186/s12889-016-3030-0 (PMC4851810; doi:10.1186/s12889-016-3030-0)
Supplement: Additional file 1: — Description hip training. (PDF 97 kb) [file 12889_2016_3030_MOESM1_ESM.pdf]

Additional File 1: Brief description of home training exercises for patients with hip OA.

Table 1: Description of phase 1 exercises for patients with hip OA (exercises for postural control are not included in the table).

**Type[1] / No**	**Exercise**	**Position**	**M-Plane[2]**	**RoI[3]**	**Muscles**	**Tools[4]**
**Week 1**						
MM1 (H)	Mobilization	Supine	S	Hip/Lumbar spine		
MM2 (H)	Mobilization	Supine	S	Hip		
MM3 (H)	Mobilization	Supine	C/T	Hip		
MM4 (H)	Motor learning	Supine	C/T	Hip	Adductors	EB
MM5 (H)	Motor learning	Supine	S	Hip/Knee	Hip extensors<br>Knee flexors	EB
MM6 (H)	Mobilization	Seated	S	Hip/Lumbar spine		
MM7 (H)	Motor learning	Seated	S	Hip	Core	
MM8 (H)	Motor learning	Seated	S	Hip/Knee/Ankle	Hip flexors/Calf	
MM9 (H)	Mobilization	Seated	C/T	Hip		
MM10 (H)	Mobilization	Seated	T	Hip/Lumbar spine		
**Week 2**						
MM11 (H)	Motor learning	Seated	S	Contact area foot	Lower extremity	
MM12 (H)	Motor learning	Seated	T	Contact area foot	Lower extremity	
MM13 (H)	Stretching	Seated	S	Hip/Knee/Ankle	Hamstrings/Calf	
MM14 (H)	Motor learning	Standing		Contact area foot	Lower extremity	
MM15 (H)	Motor learning	Standing	F	Contact area foot	Lower extremity	
MM16 (H)	Motor learning	Standing	F	Contact area foot	Lower extremity	
MM17 (H)	Stretching	Standing	F	Hip	Adductors	
MM18 (H)	Motor learning	Standing	S	Hip/Knee/Ankle	Core<br>Lower extremity	
MM19 (H)	Stretching	Standing	S	Hip/Knee	Hip flexors<br>Knee extensors	RB
MM20 (H)	Stretching	Standing	S	Hip/Knee	Hamstrings/Calf	
**Week 3**						
MM21 (H)	Mobilization	Supine	T	Hip		
MM22 (H)	Mobilization	Supine	C	Hip/Lumbar spine		
MM23 (H)	Motor learning	Supine	S	Hip/Knee	Hamstrings	
MM24 (H)	Mobilization	On the side	S	Hip/Lumbar spine		
MM25 (H)	Motor learning	On the side	T/C	Hip	External rotators<br>Abductors	
MM26 (H)	Motor learning	On the side	T/C	Hip/Knee	External rotators<br>Abductors	
MM27 (H)	Motor learning	Step position		Contact area foot	Lower extremity	
MM28 (H)	Motor learning	Step position		Contact area foot	Lower extremity	
MM29 (H)	Motor learning	Step position		Contact area foot	Lower extremity	
MM30 (H)	Motor learning	Step position	S	Hip/Knee	Flexors	

[1] MM: Motor learning and mobilization | S: Strengthening | H: Hip specific (numbers correspond to exercise book)
[2] M-Plane (Movement plane): S: Sagittal plane | F: Coronal plane | T: Transverse plane
[3] RoI: Region of interest
[4] EB: Exercise balls | RB: Elastic rubber bands | WC: Weight cuff

Table 2: Description of phase 2 exercises for patients with hip OA (exercises for postural control are not included in the table).

Type[1] / No	Exercise	Position	M-Plane[2]	RoI[3]	Muscles	Tools[4]
**Week 4**						
S1 (H)	Strengthening	Supine	S	Hip	Extensors	
S2 (H)	Strengthening	Supine	S	Hip	Flexors	
S3 (H)	Strengthening	Supine	S	Hip	Abductors	RB
S4 (H)	Strengthening	Supine	S	Hip	Adductors	EB
**Week 5**						
S5 (H)	Strengthening	Standing	S	Hip	Extensors	
S6 (H)	Strengthening	Seated	S	Hip	Flexors	
S7 (H)	Strengthening	Seated	S	Hip	Abductors	RB
S8 (H)	Strengthening	Seated	S	Hip	Adductors	EB
**Week 6**						
First home training session according to week 4, second home training session according to week 5.						
**Week 7**						
One of the exercises S1-S8 for each muscle group (hip extensors, hip flexors, hip abductors, hip adductors). Type of exercise can be chosen according to personal preferences - 4 exercises in total.						

Table 3: Description of phase 3 exercises for patients with hip OA (exercises for postural control are not included in the table).

Type[1] / No	Exercise	Position	M-Plane[2]	RoI[3]	Muscles	Tools[4]
**Week 8-11: First home training session**						
S9 (H) or<br>S13 (H)	Strengthening	Supine or<br>Stand	S	Hip	Extensors	 WC
S10 (H) or<br>S14 (H)	Strengthening	Supine or<br>Seated	S	Hip	Flexors	WC<br>WC
S11 (H) or<br>S15 (H)	Strengthening	Supine or<br>Seated	S	Hip	Abductors	RB<br>RB
S12 (H) or<br>S16 (H)	Strengthening	Supine or<br>Seated	S	Hip	Adductors	EB<br>EB
**Week 8-11: Second home training session**						
S17 (H)	Strengthening	Standing	S	Hip	Extensors	EB
S18 (H)	Strengthening	Standing	S	Hip	Flexors	EB
S19 (H)	Strengthening	Standing	S	Hip	Abductors	EB
S20 (H)	Strengthening	Standing	S	Hip	Adductors	EB
