# Supplementary material for: Effectiveness and efficiency of an 11-week exercise intervention for patients with hip or knee osteoarthritis: a protocol for a controlled study in the context of health services research
Source: BMC Public Health. 2016 Apr 30;16:367. doi: 10.1186/s12889-016-3030-0 (PMC4851810; doi:10.1186/s12889-016-3030-0)
Supplement: Additional file 3: — Excerpt of the German-language exercise manual. (PDF 2202 kb) [file 12889_2016_3030_MOESM3_ESM.pdf]

HAUPT // JANSSEN // KRAUSS // STEINHILBER

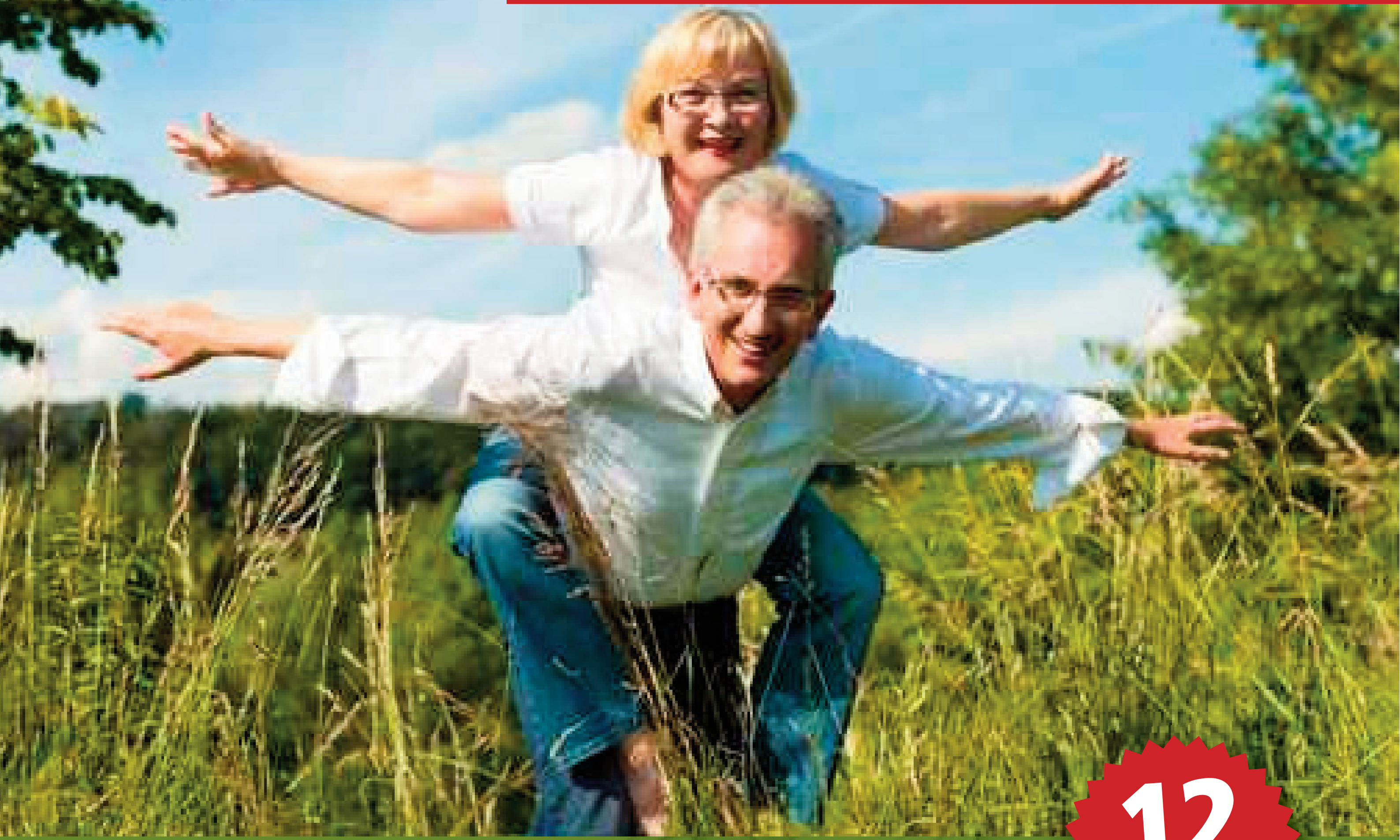

12 WOCHEN TRAINING

Das Tübinger Hüftkonzept

Von der Wissenschaft in die Praxis

1 2 3 4 5 6 7 8 9 10 11 12

12-WOCHEN-TRAINING FÜR EIN **AKTIVERES LEBEN MIT ARTHROSE**

THÜKo
Tübinger Hüftkonzept

INHALT

1 MEDIZINISCHE GRUNDLAGEN

1.1 Definition der (Hüft-) Arthrose 12
1.2 Wie häufig kommt die Hüftarthrose in der Bevölkerung vor? 12
1.3 Ursachen der Hüftarthrose 12
1.4 Symptomatik der Hüftarthrose 13
1.5 Diagnose der Hüftarthrose 14
1.6 Therapiemöglichkeiten zur Behandlung der Hüftarthrose 15
1.6.1 Allgemeines zur Therapie 15
1.6.2 Operative Therapie der Arthrose 16
1.6.3 Konservative Therapie der Arthrose 16
1.7 Sporttherapie als eine Form der Bewegungstherapie 21
1.7.1 Was ist Sporttherapie? 22
1.7.2 Wie wird Sporttherapie angewendet? 22
1.7.3 Wirkt Sporttherapie bei Hüftarthrose? 22
1.7.4 Wer bietet Sporttherapie bei Hüftarthrose an – kann ich diese auch selber durchführen? 24
1.7.5 Das Tübinger Hüftkonzept THüKo zur Behandlung der Coxarthrose 25
1.8 Literatur 26

2 WISSENSWERTES

2.1 Wissenswertes zur Anatomie der Hüfte 30
2.1.1 Knochen der Hüfte 31
2.1.2 Knorpel, Bänder, Kapsel 32
2.1.3 Hüftmuskulatur 33
2.2 Wissenswertes zur Biomechanik des Hüftgelenks 34
2.2.1 Die Hüfte als Kugelgelenk 34
2.2.2 Bewegungsausmaß der Hüfte 35
2.2.3 Gelenkbelastungen der Hüfte 35
2.2.4 Achsgerechtes Belasten der Hüfte 35
2.3 Wissenswertes zum Training 36
2.3.1 Training bei Hüftarthrose 36
2.3.2 Trainingsmethoden zur Wahrnehmungsschulung und Mobilisation 37
2.3.3 Krafttraining und Trainingsmethoden 37
2.3.4 Gleichgewichtsfähigkeit und Trainingsmethoden 42
2.3.5 THüKo: Trainingsmethoden auf einen Blick 44
2.4 Wissenswertes zur Trainingsdokumentation 45
2.4.1 Schmerzen beim Training 46
2.5 Phasen des Heimtrainingsprogramms von THüKo 47
2.6 Literatur 48

3 VON DER THEORIE ZUR PRAXIS

3.1 Einführung 50
3.1.1 Gesundheitliche Risiken und Kontraindikationen 50
3.1.2 Wichtige Informationen zur erfolgreichen Umsetzung des Heimtrainingsprogramms 50
3.1.3 Ausgangsstellungen 51
3.1.4 Übungsmaterialien 51
3.1.5 Rahmenbedingungen für ein erfolgreiches Training 57
3.2 Das Training beginnt 57
3.2.1 Phase I (Woche 1 – 3) 57
3.2.2 Phase II (Woche 4 – 8) 95
3.2.3 Phase III (Woche 9 – 12) 140

Die körperliche Symptomatik steht hierbei in Zusammenhang mit einer Reduktion der gesundheitsbezogenen Lebensqualität[20–23]. Im weiteren Verlauf der Erkrankung können immer wieder akut entzündliche Veränderungen im betroffenen Gelenk mit entsprechender Ergußbildung und Schmerzen auftreten[24]. Man spricht dann von einem »aktivierten« Zustand der Arthrose. Je weiter die Gelenkschädigung zunimmt, desto häufiger leidet der Betroffene an Ruhe- und Nachtschmerzen.

Der Verlauf der Arthrose unterscheidet sich individuell sehr stark und ist u. a. abhängig von der Lokalisation, der Belastung und der durchgeführten Behandlung[25].

1.5 DIAGNOSE DER HÜFTARTHROSE

Schon die Anamnese weist den behandelnden Arzt auf die Diagnose Arthrose hin. In den Leitlinien der medizinischen Fachgesellschaften (AWMF) sind Fragen zur speziellen Anamnese aufgeführt. Bei der Frage nach Schmerzen wird meistens die Leistenregion angegeben. Die Beschwerden können jedoch auch in Richtung Oberschenkel bis hin zum Knie ausstrahlen. Anlaufschmerzen müssen von regelmäßig auftretenden belastungsabhängigen Hüftbeschwerden (isoliert oder in Kombination mit Nachtschmerzen) unterschieden werden[26]. Typisch für die Hüftarthrose ist die sogenannte Morgensteifigkeit, die bis zu 60 Minuten anhalten kann.

Bei der Hüftgelenksarthrose sind die Schmerzen oft in der Leiste lokalisiert. Sie können jedoch in Richtung Oberschenkel und Kniegelenk ausstrahlen.

In vielen Fällen klagen Patienten über eine Abnahme der Lebensqualität, die durch die krankheitsbedingte Verringerung der Teilhabe am sozialen Leben sowie die erschwerte Durchführung bestimmter Aktivitäten im Alltag bedingt sein kann.

Nach der Anamnese folgt die körperliche Untersuchung, die die Gelenkbeweglichkeit, den Muskelstatus und den Zustand der benachbarten Gelenke erfasst. In einer allgemeinen Untersuchung wird eine Inspektion hinsichtlich Schwellung, Rötung und Überwärmung, Beurteilung der Durchblutung und eine orientierende neurologische Untersuchung (Motorik, Sensibilität) der unteren Extremitäten durchgeführt. Bei Verdacht auf Hüftarthrose erfolgt eine spezielle Untersuchung. Dabei werden Gangbild, Beckenstand und Beinlänge sowie die Funktion der Bein- und Gesäßmuskulatur beurteilt[26]. Spezifische Untersuchungen der schmerzhaften Hüfte folgen.

Bei klinischem Verdacht auf einen degenerativen Knorpelschaden wird der behandelnde Arzt eine Röntgenuntersuchung veranlassen, um das Stadium der Arthrose zu diagnostizieren. Im Röntgenbild sind die Veränderungen des Knorpelüberzugs selbst nicht zu sehen. Gut zu erkennen sind jedoch die knöchernen Veränderungen, z. B. eine Verschmälerung des Gelenksspalts, die auf eine Abnahme der Knorpeldicke schließen lässt. Auch eine zunehmende Verkalkung des unter dem Knorpel liegenden Knochens ist ein Zeichen einer Arthrose. Im weiteren Verlauf der Erkrankung bilden sich die osteophytären Randausziehungen aus, die ebenfalls gut im Röntgen zu erkennen sind. Im Frühstadium der Arthrose kann eine Kernspintomographische

Abbildung 1:
Auf der rechten Bildseite ist eine Arthrose des Hüftgelenks mit den klassischen knöchernen Veränderungen zu sehen.

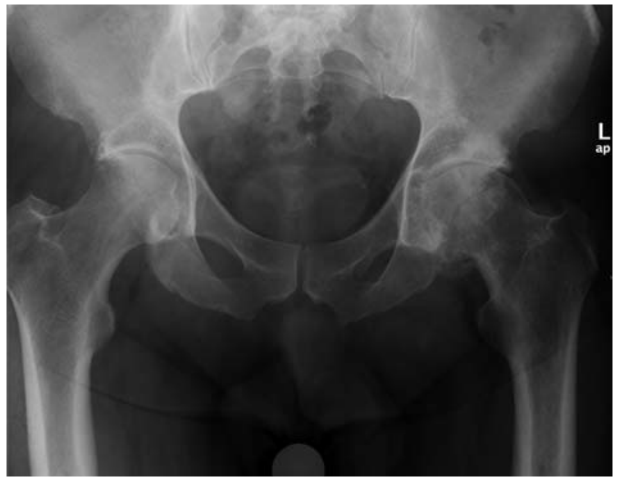

Untersuchung (Magnetresonanztomographie = MRT) zur Diagnostik des Knorpelschadens herangezogen werden. Bei dieser Untersuchung können nicht nur Knochen sondern insbesondere auch Knorpel- und Weichteilgewebe beurteilt werden.

1.6 THERAPIEMÖGLICHKEITEN ZUR BEHANDLUNG DER HÜFTARTHROSE

1.6.1 Allgemeines zur Therapie

Allen Bemühungen der Wissenschaftler und Forscher zum Trotz ist die Arthrose bis zum heutigen Tage nicht heilbar[27]. Arthrotisch bedingte Schäden des Knorpels sind irreparabel. Eine vollständige Wiederherstellung ist nicht mehr möglich[28]. Die Zielsetzungen der Therapie liegen demnach in der Verringerung der Gelenkschmerzen und Gelenksteifheit sowie der Aufrechterhaltung der Gelenkbeweglichkeit. Die Verbesserung der körperlichen Funktionsfähigkeit sowie die Verringerung der Behinderung durch die Erkrankungen sind neben der Verbesserung der gesundheitsbezogenen Lebensqualität weitere wichtige Aspekte. Die Therapie zielt ferner da rauf ab, das Fortschreiten der Erkrankung und die Destruktion des Gelenkes zu begrenzen und die Patienten über die Erkrankung sowie den Umgang mit dieser zu informieren[29].

Ziele der Arthrosetherapie:
- **Verringerung von Schmerz und Gelenksteifigkeit**
- **Aufrechterhaltung der Gelenkbeweglichkeit**
- **Verbesserung der körperlichen Funktionsfähigkeit**
- **Verbesserung der gesundheitsbezogenen Lebensqualität**
- **Wissensvermittlung für ein arthrosegerechtes Alltagsverhalten**

Das Behandlungsspektrum berücksichtigt medikamentöse, nicht-medikamentöse und operative Therapieverfahren[20]. Mehr als 50 Therapieoptionen stehen für die Behandlung der Arthrose an Hüft- und Kniegelenk zur Verfügung. Um entscheiden zu können, welche Therapie für den jeweils Betroffenen die beste Behandlungsmöglichkeit darstellt, wurden durch die medizinischen Fachgesellschaften Orientierungshilfen für das therapeutische Vorgehen formuliert[26; 30; 31]. Der Berücksichtigung individueller Voraussetzungen und Rahmenbedingungen soll hierbei ein hoher Stellenwert im Sinne einer personenorientierten Therapie eingeräumt werden.

Wichtige Kriterien sind dabei:
- das Stadium der Erkrankung
- der angegebene Hüftschmerz
- das Lebensalter
- der körperliche Allgemeinzustand
- die Begleiterkrankungen
- der Aktivitätsgrad des Patienten
- die berufliche Situation
- die persönlichen Wünsche und Präferenzen des Patienten
- der bisherige Krankheitsverlauf
- die Einschränkung der Lebensqualität
- die Rahmenbedingungen vor Ort

Es gilt mit dem Patienten zu entscheiden, ob eine konservative Therapie oder bereits eine operative Therapie durchgeführt werden sollte. Die Leitlinien der Fachgesellschaften betonen dabei, dass diese Entscheidung »nur individuell gemeinsam mit dem über das Krankheitsbild informierten und über die Therapiemöglichkeiten aufgeklärten Patienten getroffen werden kann, allgemeingültige verbindliche Regeln existieren nicht«[26].

Die Entscheidung, welche Therapie durchgeführt werden soll muss individuell gemeinsam mit dem über das Krankheitsbild informierten und über die Therapiemöglichkeiten aufgeklärten Patienten getroffen werden.

1.7.1 Was ist Sporttherapie?

Bei der Sporttherapie geht es nicht um Wettkampf, Leistungssport oder das Erlernen und Ausüben spezieller Sportarten. Vielmehr bedient sich die Sporttherapie geeigneter Mittel des Sports, um gestörte körperliche, psychische und soziale Funktionen zu kompensieren, zu regenerieren, Sekundärschäden vorzubeugen und gesundheitlich orientiertes Verhalten zu fördern[34].

Die Inhalte der Sporttherapie berücksichtigen mehrere Dimensionen: das Trainieren motorischer Fähigkeiten wie Kraft, Beweglichkeit und Ausdauer sowie das Erlernen und Üben motorischer Fertigkeiten im Sinne von Bewegungsmustern. Die Schulung solcher Fertigkeiten ermöglicht eine Verbesserung der für den Patienten alltagsrelevanten Handlungen und Aktivitäten. Die ergänzende Vermittlung von Kenntnissen und Wissen schafft zudem die Voraussetzung für eine selbständige und langfristige gesundheitsbezogene Handlungskompetenz. Im Rahmen der Therapie soll eine langfristige Motivation zur Durchführung von Eigenübungsprogrammen und zur Erreichung eines körperlich aktiven Lebensstils ermöglicht werden[60]. Erst durch die Ermutigung zur Aufnahme und Beibehaltung eines regelmäßigen körperlichen Trainings kann dieses Training auch wirken[29].

Die verschiedenen Inhalte der Sporttherapie spiegeln das ganzheitliche Gesundheitsverständnis der Weltgesundheitsorganisation WHO wider. Das zugrundeliegende bio-psycho-soziale Denkmodell orientiert sich nicht primär an den Defiziten, die ein Mensch aufgrund seiner Krankheit hat, sondern beschreibt und klassifiziert relevante Komponenten der Gesundheit. Neben den Strukturen und Funktionen des Körpers gehören die Komponenten der Aktivitäten (beispielsweise die Fertigkeit, alltagsrelevante Handlungen durchführen zu können) sowie die Teilhabe am gesellschaftlichen Leben zu der ganzheitlichen Betrachtung und Klassifikation der Gesundheit. Hierbei werden sowohl umweltbezogene als auch personenbezogene Faktoren in die Betrachtung einbezogen[60;61].

1.7.2 Wie wird Sporttherapie angewendet?

Sporttherapie ist sowohl an Land als auch im Wasser möglich. Dabei kann das Training in der Gruppe, in einer individuellen 1:1-Betreuung, in Eigenregie als Heimtraining oder in kombinierter Form erfolgen. Gerätetraining, Walking und Ergometertraining finden genauso Anwendung wie beispielsweise Trainingsformen aus dem Thai Chi oder aus dem Bereich der physiotherapeutischen Behandlungsmethoden. Die Gewichtung der Trainingsschwerpunkte (z. B Krafttraining, Gruppenspiele, Gleichgewichtstraining, Bewegungsschulung etc.) kann bei den unterschiedlichen Angebotsformen stark variieren. Gleiches gilt auch für die Auswahl der Trainingsmittel (z. B. elastische Bänder oder Gewichtsmanschetten) und die Belastungsdosierung (Dauer der Einheit, Umfang der Trainingsinhalte, Intensität der Trainingsinhalte und Häufigkeit des Trainings).

Der Schwerpunkt des vorliegenden Buches liegt in der detaillierten Darstellung eines hüftspezifischen Heimtrainingsprogramms. Weitere Informationen zur Trainingsgestaltung und Trainingssteuerung sowie zu relevantem Hintergrundwissen sind den Kapiteln »Wissenswertes« und »von der Theorie zur Praxis« zu entnehmen.

1.7.3 Wirkt Sporttherapie bei Hüftarthrose?

Der behandelnde Arzt entscheidet in der Regel mit seinem Patienten, welche Therapie zur Behandlung seiner Erkrankung sinnvoll ist. Die Entscheidung des Arztes basiert üblicherweise auf seinem Wissen und seiner Erfahrung. Seit dem vergangenen Jahrhundert ermöglichen eine immer größer werdende Anzahl wissenschaftlicher Untersuchung enorme Fortschritte in der medizinischen Versorgung. Studien zur Wirksamkeit therapeutischer Maßnahmen helfen dem Arzt bei der Auswahl der optimalen Behandlungsform[62].

Evidenzbasierte Medizin – so bezeichnet man die Nutzung neuester wissenschaftlicher Erkenntnisse bei der Auswahl der optimalen Behandlung für den einzelnen Patienten. Voraussetzung hierfür ist ein in wissenschaftlichen Studien erfolgter Wirksamkeitsnachweis der Therapie. Dies gilt nicht nur für operative oder medikamentöse Verfahren sondern gleichsam für die sport- und bewegungstherapeutische Behandlung der Arthrose. Werden Sport und Bewegung als Therapie angewendet, so muss auch für diese Therapieformen der Wirksamkeitsnachweis im Sinne einer Schmerzreduktion sowie Verbesserung der körperlichen Funktionsfähigkeit und der gesundheitsbezogenen Lebensqualität erbracht werden.

!

Als evidenzbasierte Medizin bezeichnet man die Nutzung neuester wissenschaftlicher Erkenntnisse bei der Auswahl der optimalen Behandlung für den einzelnen Patienten.

Doch was wissen wir derzeit über die Wirksamkeit der Sporttherapie bei einer vorliegenden Arthrose des Hüft- oder Kniegelenks? Wo bestehen noch Wissenslücken, die es in Zukunft durch weitere wissenschaftliche Untersuchungen zu schließen gilt? Die folgende Darstellung der Ergebnisse wissenschaftlicher Studien, die sich mit der Wirksamkeit der Sporttherapie bei Knie- und Hüftarthrose befasst haben, sollen eine Antwort auf diese beiden Fragen geben.

Wissenschaftliche Untersuchungen zur Sporttherapie bei Arthrose berücksichtigen in den meisten Fällen Patienten mit Kniearthrose. Erst seit wenigen Jahren können auch konkrete Aussagen zur Wirksamkeit der Sporttherapie bei Hüftarthrose gemacht werden. Zuvor basierten die Empfehlungen für körperlicheres Training bei Hüftarthrose auf der Annahme, dass die für die Kniearthrose beschriebenen Wirkmechanismen der Intervention auch auf Beschwerden am Hüftgelenk übertragbar sind[29;63]. Es ist jedoch davon auszugehen, dass es unterschiedliche Behandlungseffekte in Abhängigkeit vom Ort der Arthrose gibt und der wissenschaftliche Nachweis für jedes Gelenk für sich erfolgen sollte. So sind in Hinblick auf die Risiko- und Einflussfaktoren für das Fortschreiten der Erkrankung bereits Unterschiede in Abhängigkeit von dem von Arthrose betroffenen Gelenk bekannt. Gleiches gilt für die Zusammenhänge zwischen Schmerzsymptomatik und struktureller Veränderung des Gelenkes[20]. Eine gesonderte Betrachtung der Effektivität sporttherapeutischer Interventionen bei Patienten mit Hüftarthrose wird deshalb nachdrücklich gefordert[20;64].

2008 wurde erstmalig eine zusammenfassende Analyse (Meta-Analyse) wissenschaftlicher Studien veröffentlicht, die spezifische Aussagen zur Wirksamkeit sporttherapeutischer Interventionen bei Hüftarthrose zulässt und hierbei der Therapieform eine schmerzlindernde Wirkung zuschreibt. Die Analyse berücksichtigte sowohl Studien mit Therapieformen an Land, als auch im Wasser[65]. Bei alleiniger Betrachtung der landbasierten Therapieformen in einer gesonderten Meta-Analyse kommen die zum Teil gleichen Autoren zu dem Schluss, dass bisher lediglich kleine Behandlungseffekte eines landbasierten Trainings in Hinblick auf die Schmerzreduktion nachgewiesen werden konnten. Der Nachweis der Effektivität eines Trainings zur Verbesserung der körperlichen Funktionsfähigkeit konnte bis dato nicht erbracht werden[64]. Aktuelle Studien geben jedoch auch hier deutliche Hinweise, dass Hüftarthrosepatienten von der Sporttherapie profitieren können. So wurde die Wirksamkeit einer zusätzlich zur Standardversorgung durch den Hausarzt durchgeführte Sporttherapie untersucht. Die ergänzende Sporttherapie führte zu keiner zusätzlichen Schmerzreduktion. Allerdings konnte die körperliche Funktionsfähigkeit kurz- und mittelfristig durch das Training verbessert werden[68]. In einer von den Autoren des vorliegenden Buches durchgeführten Studie konnte gezeigt werden, dass ein zusätzlich zu einem wöchentlich stattfindenden Gruppentraining durchgeführtes Heimtrainingsprogramm über einen Zeitraum von 8 Wochen die Kraftfähigkeiten der hüftumgebenden Muskulatur verbessern kann[67]. In einer Folgestudie wurde dann das Tübinger Hüftkonzept (THüKo), welches dem hier vorgestellten Heimtrainingsprogramm zugrunde liegt an mehr als 200 Probanden mit Coxarthrose wissenschaftlich überprüft. Die Ergebnisse zeigen, dass das über 12 Wochen durchgeführte THüKo-Trainings-

programm zu einer Verbesserung der Kraftfähigkeiten, der Koordination, der Schmerzsymptomatik sowie der körperlichen Funktionsfähigkeit führt[68–70].

1.7.4 Wer bietet Sporttherapie bei Hüftarthrose an – kann ich diese auch selber durchführen?

Gezielte sporttherapeutische Angebote zur Behandlung der Hüftarthrose werden in den meisten Fällen ärztlich verordnet und durch einen Therapeuten geplant und dosiert. Angebote finden sich sowohl im ambulanten, als auch im stationären Bereich. Die Bewegungs- und Sporttherapie spielt beispielsweise im Rahmen der stationären Rehabilitation eine wesentliche Rolle. Auch im ambulanten Bereich können sporttherapeutische Maßnahmen vom Arzt verordnet werden. Nach dem Sozialgesetzbuch IX gehören der Rehabilitationssport und das Funktionstraining zu den ergänzenden Leistungen der Rehabilitation. Die Bedingungen für die genannten Leistungen wurden 2011 von der Bundesarbeitsgemeinschaft Rehabilitation (BAR) durch die Rehabilitationsträger und die Leistungserbringer sowie Selbsthilfegruppen in der Rahmenvereinbarung über den Rehabilitationssport und das Funktionstraining festgelegt[34;71]. Funktionstraining wird im Allgemeinen von den örtlichen Arbeitsgemeinschaften der Landesverbände der Deutschen Rheumaliga angeboten, Rehabilitationssport hingegen von örtlichen Rehabilitationssportgruppen der Landesbehinderten-Sportverbände, die dem Deutschen Behindertensportverband (DBS) angehören[71]. An dieser Stelle muss jedoch angemerkt werden, dass zum jetzigen Zeitpunkt noch kein flächendeckendes Netz für ein hüftspezifisches sporttherapeutisches Angebot in den genannten Organisationen vorliegt. Nähere Informationen zu bestehenden Angeboten und den organisatorischen Rahmenbedingungen sind der jeweiligen Homepage der Leistungserbringer zu entnehmen. Für das Funktionstraining verweisen wir auf die Seiten der Deutschen Rheumaliga (www.rheuma-liga.de/arthrose/), für den Rehabilitationssport auf die Seiten des Deutschen Behindertensportverbandes (www.dbs-npc.de/sportentwicklung-rehabilitations-sport-aktuelles.html).

> **!** **Sporttherapie kann sowohl ambulant als auch stationär durchgeführt werden und wird in der Regel vom Arzt verordnet.**
>
> **Angebote gibt es von Rehabilitationseinrichtungen, Verbänden und Selbsthilfegruppen. Auch Physiotherapiepraxen und Sportvereine sind mögliche Anbieter passender Sportprogramme.**

Während Funktionstraining und Rehabilitationssport in der Gruppe angeboten werden, kann der behandelnde Arzt bei gegebener Indikation allgemeine Krankengymnastik, gerätegestützte Krankengymnastik oder Übungsbehandlung verordnen. Diese Maßnahmen berücksichtigen auch Elemente der Sporttherapie. Inhalt, Umfang und Häufigkeit der verordnungsfähigen Heilmittel richtet sich für Versicherte der gesetzlichen Krankenversicherungen nach dem gültigen Heilmittelkatalog. Für die Diagnose »Arthrose« regt diese Richtlinie zudem an, die Patienten in die eigenständige Ausführung eines Übungsprogrammes einzuweisen und zudem eine Gelenkschule anzubieten. Schriftliche Unterlagen werden hierbei empfohlen[72]. Das vorliegende Buch soll in diesem Zusammenhang ein hilfreiches Instrument sein, um den Patienten beim eigenständigen Üben gezielt zu unterstützen und anzuleiten.

Neben den ärztlich verordneten sporttherapeutischen Angeboten gibt es auch andere Anbieter, die ein sinnvolles Trainingsangebot für Patienten mit Hüftarthrose bereithalten. So kann man vielerorts im »normalen Sportverein«, an der Volkshochschule, in Fitnessstudios oder in Physiotherapiepraxen passende Möglichkeiten für einen hüftgelenkfreundlichen Sport finden.

Wer lieber zuhause übt oder das Training in der Gruppe durch ein zusätzliches Heimtrainingsprogramm ergänzen möchte, kann mit Hilfe des vorliegenden Buches eigenständig ein hüftspezifisches Trainingsprogramm absolvieren. Die praktischen Übungsanweisungen werden hierbei durch wichtige Informationen zu Arthrose, anatomischen Grundlagen sowie Grundlagen der Trainingssteuerung und Trainingsdokumentation ergänzt.

1.7.5 Das Tübinger Hüftkonzept THüKo zur Behandlung der Coxarthrose

Das Tübinger Hüftkonzept THüKo basiert auf langjährigen Erfahrungen aus den »Hüftsportgruppen«, die in Tübingen erstmalig 1995 ins Leben gerufen wurden. Im Rahmen des Kursangebotes trainieren je Gruppe 10 – 15 Personen einmal wöchentlich gemeinsam mit einem Übungsleiter. Das Angebot kann als Rehabilitationssport im Sinne des §44 Abs. 1 Nr. 3 Sozialgesetzbuch IX vom Arzt verordnet werden (vgl. hierzu Kapitel 1.7.4).

> **!** **Sporttherapie kann durch ein zusätzliches Heimtrainingsprogramm sinnvoll ergänzt werden. Das Tübinger Hüftkonzept (THüKo) ist dafür beispielhaft. Es beinhaltet ein 12-wöchiges Heimtrainingsprogramm, welches speziell auf die Bedürfnisse der Patienten mit Hüftarthrose abgestimmt wurde und eigenständig zuhause durchführbar ist.**

Neben dem einmal wöchentlich stattfindenden Gruppentraining berücksichtigt THüKo im Vergleich zu den klassischen Hüftsportgruppen auch ein zweimal wöchentlich durchzuführendes Heimtrainingsprogramm in Eigenregie. Das Trainingsprogramm ist speziell auf die Bedürfnisse der Patienten mit Hüftarthrose abgestimmt. Neben dem Training in der Gruppe lernt der Patient in Heimtrainingseinheiten eigenständig, die Muskulatur zu kräftigen, das Gleichgewicht zu schulen und die Beweglichkeit des betroffenen Gelenks zu verbessern. Daneben werden wichtige theoretische Inhalte zur Trainingsdurchführung, zu einem hüftgerechten Verhalten im Alltag und zu medizinischen Grundlagen vermittelt, um den Patienten zum Experten in eigener Sache zu machen. In den vergangenen Jahren wurde nun die Wirksamkeit des Trainingsprogramms im Rahmen einer wissenschaftlichen Studie nachgewiesen (siehe auch Kapitel 1.7.3).

Das in dem vorliegenden Buch vorgestellte Heimtrainingsprogramm ist ein wesentlicher Bestandteil des Tübinger Hüftkonzepts. Um es Patienten zugänglich zu machen, wurde es als 12-Wochen-Trainingsprogramm in Buchform aufbereitet und kann so von den Patienten eigenständig zuhause durchgeführt werden.

2 WISSENSWERTES

Das vorliegende Kapitel »Wissenswertes« liefert Informationen über:

- die Anatomie der Hüfte
- die Biomechanik des Hüftgelenks
- das Training bei Hüftarthrose
- den Aufbau des Heimtrainingsprogramms von THüKo
- die schriftliche Dokumentation des Trainings.

Diese Informationen helfen das Heimtrainingsprogramm von THüKo in seinen Einzelheiten zu verstehen, den Sinn jeder Übung zu erkennen und die Bedeutung eines regelmäßigen Trainings zu verinnerlichen.

Außerdem steht dieses Kapitel in direktem Zusammenhang mit dem praktischen Teil des Buches (Kapitel 3) und sollte deshalb beim Training stets griffbereit sein. Das dritte Kapitel enthält auf den Seiten »Aufgepasst« immer wieder Verweise, die Sie zu den entsprechenden Abschnitten dieses Theorieteils führen. Hier können Sie dann die theoretischen Grundlagen bei Bedarf nochmals nachlesen.

2.1 WISSENSWERTES ZUR ANATOMIE DER HÜFTE

Das Hüftgelenk ist die Schnittstelle von Rumpf und unterer Extremität. Der Übergangsbereich von der Lendenwirbelsäule bis zum Oberschenkel (siehe Abbildung 1) stellt anatomisch und funktionell ein sehr komplexes Gebilde dar. Dies wirkt sich auf bestimmte Aspekte des Trainings aus. Beispielsweise können Knochenpunkte des Beckens mit der Hand ertastet und bei verschiedenen Übungen genutzt werden, um die Bewegung im Hüftgelenk zu kontrollieren. Durch die Nähe des Hüftgelenks zur Lendenwirbelsäule wirken viele der Übungen auf beide anatomische Bereiche. Daraus ergibt sich eine besondere Anforderung an die hüftspezifischen Übungen: der Gesundheitszustand der Lendenwirbelsäule darf nicht außer Acht gelassen werden, um dort bestehende Beschwerden nicht zu verstärken oder gar auszulösen.

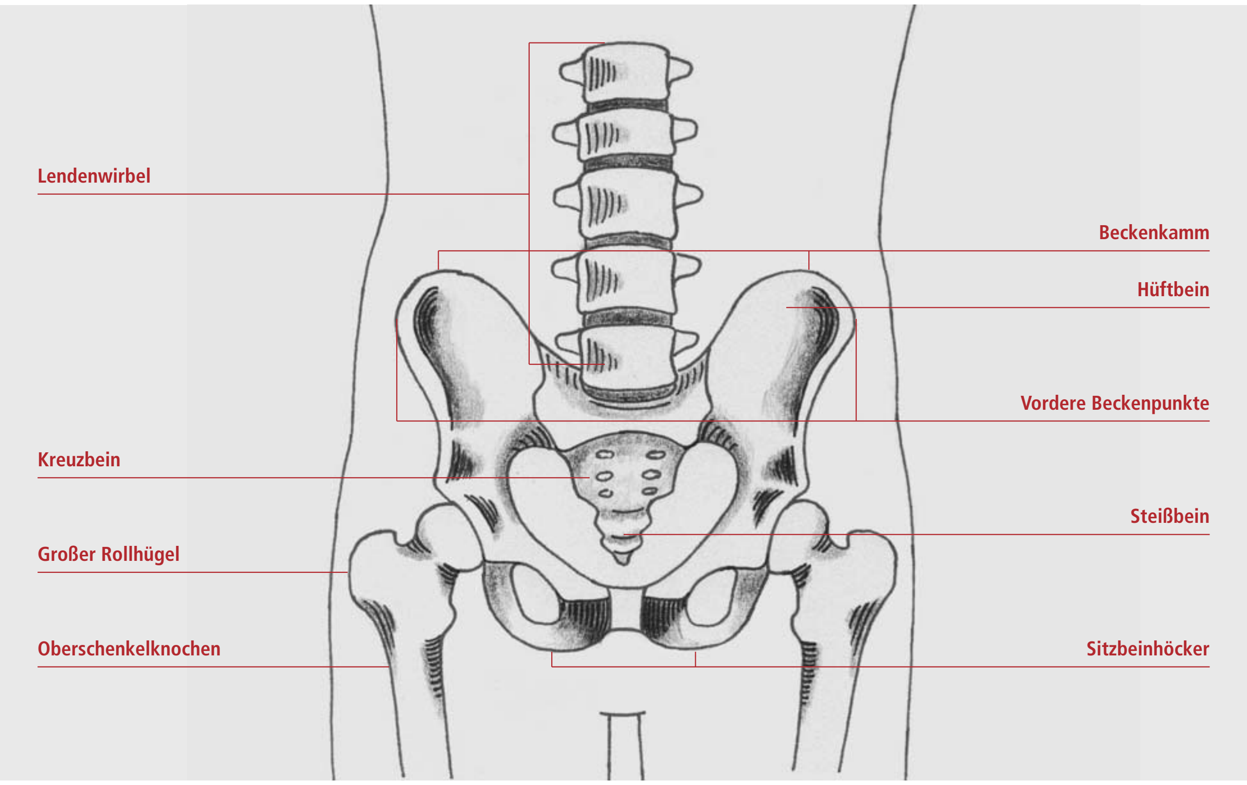

Abbildung 1: Hüft-Becken-Übersicht von vorne

2.1.1 Knochen der Hüfte

Die Lendenwirbelsäule mit ihren fünf Wirbelkörpern setzt sich im Kreuzbein fort, dem sich das Steißbein anschließt. Der obere Anteil des Kreuzbeins bildet zusammen mit dem linken und rechten Hüftbein den Beckengürtel (siehe Abbildung 2). Das knöcherne Becken besteht aus einem rechten und linken Hüftbein. Jedes wiederum besteht aus drei Knochen: Sitzbein, Schambein und Darmbein. Gemeinsam bilden sie die knöcherne Grundlage der Hüftpfanne[1] (siehe Abbildung 3).

Die Hüftpfanne ist eine halbkugelförmige Vertiefung und stellt den rumpfnahen Gelenkpartner des Hüftgelenks dar. Der rumpfferne Gelenkpartner ist der kugelförmige Hüftkopf, der sich am oberen Ende des Oberschenkelknochens befindet. Von ihm aus verläuft der etwa fünf Zentimeter lange Schenkelhals nach außen bis zum langen Schaft des Oberschenkelknochens. Am Übergang von Schenkelhals zu Oberschenkelknochen befindet sich außen der »große Rollhügel«, der mehreren Muskeln als Ansatz dient (siehe Abbildung 4).

Verschiedene knöcherne Orientierungspunkte lassen sich gut ertasten bzw. erspüren und damit zur Bewegungskontrolle beim Training einsetzen. Dazu zählen die vorderen Beckenpunkte am Darmbein. Sie stellen den vorderen Teil des Beckenkamms dar, der wiederum den oberen Rand des Beckens bildet. Dadurch geben die vorderen Beckenpunkte Auskunft über die Stellung des Beckens. Diese Punkte werden bei den Übungen häufig zur Kontrolle der Bewegung eingesetzt.

Die Sitzbeinhöcker des Sitzbeins sind der untere Teil des Beckens. Wie der Name vermuten lässt, stellen sie die knöcherne Auflagefläche im Sitz dar und können deshalb in dieser Position einfach erspürt und damit zur Bewegungskontrolle genutzt werden.

Die folgende Tabelle (siehe Tabelle 1) gibt eine Übersicht über die beschriebenen knöchernen Orientierungspunkte. Übungen, in denen diese knöchernen Orientierungspunkte zur Bewegungskontrolle eingesetzt werden, sind mit den entsprechenden Hinweisen gekennzeichnet.

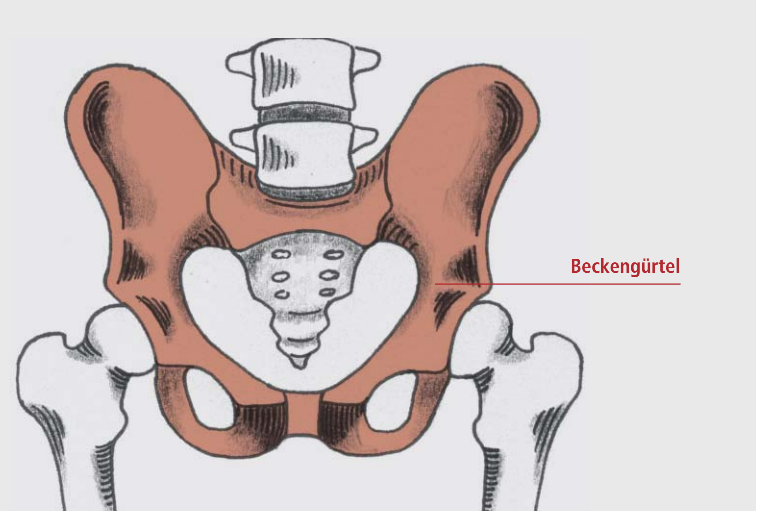

Abbildung 2: Beckengürtel von vorne

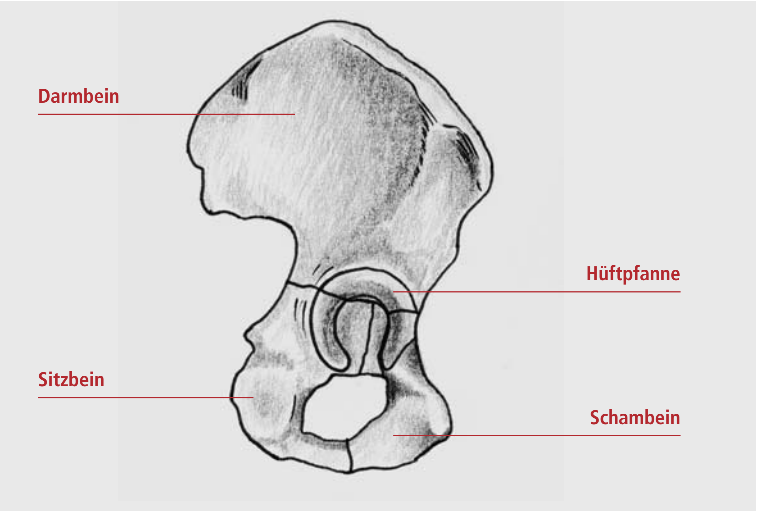

Abbildung 3: Rechtes Hüftbein von der Seite

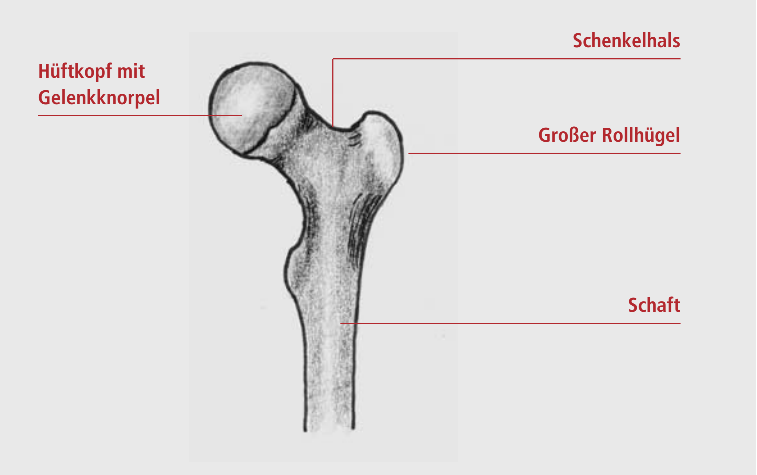

Abbildung 4: Linker Hüftkopf und gelenknaher Oberschenkel von vorne

Tabelle 1: Knöcherne Orientierungspunkte

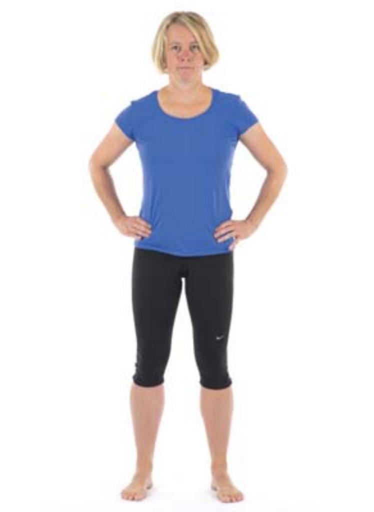	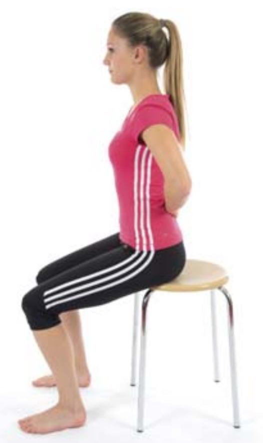	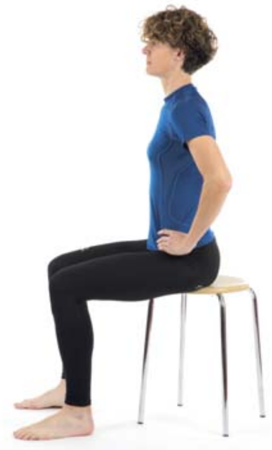
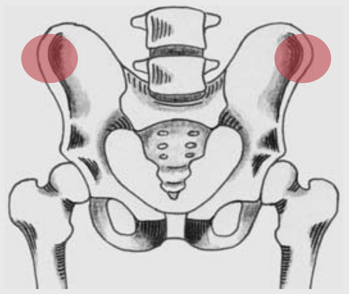	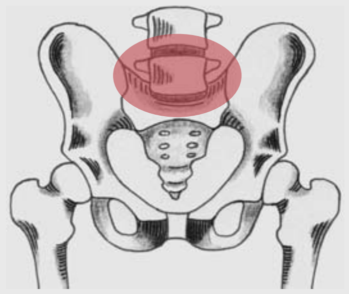	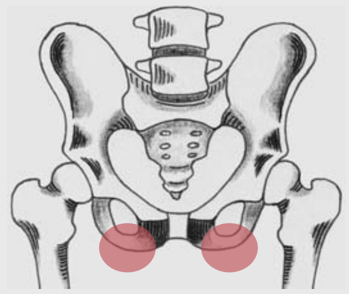
Die vorderen Beckenpunkte sind vorne seitlich mit den Zeigefingern zu tasten. Sie können einfach erreicht werden, indem die Hände mit dem Daumen nach hinten und dem Zeigefinger nach vorne auf den Beckenkamm gelegt werden. Der Zeigefinger ertastet nun den am weitesten nach vorne stehenden Knochenpunkt.	Der Übergangsbereich von der Lendenwirbelsäule zum Kreuzbein kann durch Auflegen des Handrückens quer zur Lendenwirbelsäule kontrolliert werden.	Die beiden Sitzbeinhöcker sind als harte Auflagepunkte im Sitz spürbar.

2.1.2 Knorpel, Bänder, Kapsel

Die knöchernen Gelenkpartner (Hüftkopf und Hüftpfanne) werden von einer Knorpelschicht überzogen und von einem festen Kapsel-Band-Apparat umgeben. Der Knorpel gewährleistet ein reibungsarmes Gleiten der Gelenkflächen und federt Stoßbelastungen ab.

Die umgebende Kapsel schützt als äußere Hülle den Gelenkinnenraum. Sie wird von Schleimhaut ausgekleidet, die die Gelenkflüssigkeit (Synovia) produziert. Diese Flüssigkeit dient dem Gelenk als »Schmiermittel« und enthält Nährstoffe, die den Knorpel versorgen. Zudem befinden sich in der Kapsel unterschiedliche Rezeptoren, die Informationen über Gelenkstellung und -bewegung sowie Schmerzen an das Gehirn weiterleiten.

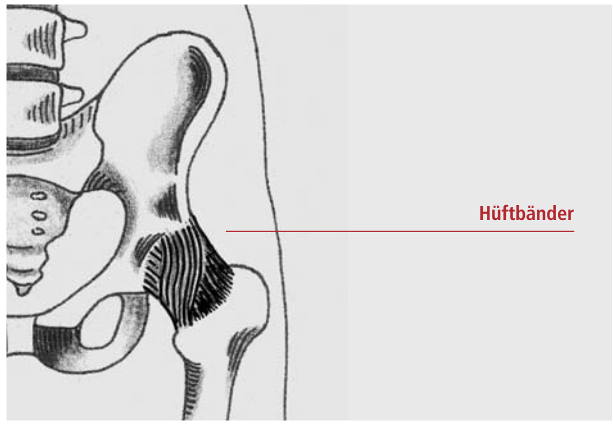

Abbildung 5: Bänder der Hüfte

Die mit der Kapsel eng verbundenen Bänder begrenzen bestimmte Bewegungen und verleihen dem Hüftgelenk zusammen mit der Muskulatur seine Stabilität (siehe Abbildung 5).

2.1.3 Hüftmuskulatur

Die Muskulatur ist in der Lage sich über physiologische, energieverbrauchende und durch Nerven gesteuerte Vorgänge zu kontrahieren und damit das Gelenk zu bewegen und zu stabilisieren[2]. In den Sehnen, die die Muskeln und Knochen verbinden sowie in der Muskulatur selber befinden sich Rezeptoren, mit denen Längen- oder Spannungsänderungen der Muskulatur wahrgenommen werden. Diese Informationen werden im zentralen Nervensystem des Rückenmarks und des Gehirns verarbeitet und mit entsprechenden Muskelaktionen beantwortet[3].

Die Hüftmuskulatur wird in Funktionsgruppen eingeteilt. Die Einteilung entspricht der Richtung der Gelenkbewegung, die durch die jeweilige Muskulatur ermöglicht wird[2]. Ausgehend vom aufrechten Stand unterscheidet man folgende Muskelgruppen:

- Hüftstrecker (Hüftextensoren) strecken das Hüftgelenk und bewegen das Bein dabei nach hinten.
- Hüftbeuger (Hüftflexoren) beugen das Hüftgelenk und bewegen das Bein dabei nach vorne
- Hüftabspreizer (Hüftabduktoren) spreizen das Bein im Hüftgelenk seitlich ab
- Hüftadduktoren führen das Bein im Hüftgelenk seitlich nach innen, auch aus abgespreizter Position
- Hüftinnenrotatoren drehen das Bein im Hüftgelenk nach innen
- Hüftaußenrotatoren drehen das Bein im Hüftgelenk nach außen

Hüftstrecker
Die das Hüftgelenk streckende Muskulatur befindet sich hinter dem Hüftgelenk. Sie verläuft vom Becken bis zum Ober- und Unterschenkel. Die zum Oberschenkel verlaufenden Muskeln bewegen nur das Hüftgelenk, die zum Unterschenkel verlaufenden Muskeln das Hüft- und Kniegelenk.

Hüftbeuger
Ähnlich verhält es sich mit den vor dem Hüftgelenk gelegenen Hüftbeugern. Sie verlaufen vom Becken bzw. der Lendenwirbelsäule bis zum Ober- und Unterschenkel. Daher haben manche Hüftflexoren neben ihrer Bewegungsfunktion im Hüftgelenk auch eine Bewegungsfunktion im Kniegelenk.

Hüftabspreizer
Die abspreizende Muskulatur zieht seitlich außen über das Hüftgelenk. Sie nimmt ihren Ursprung außen am Becken und zieht zum großen Rollhügel und am seitlichen Oberschenkel bis zum Kniegelenk.

Hüftadduktoren
Die Hüftadduktoren als muskuläre Gegenspieler der abspreizenden Muskulatur entspringen am Schambein und ziehen zum größten Teil zur Innenseite des Oberschenkels[2].

» Abbildung 6 zeigt den Verlauf und die Lage einiger Hauptmuskeln der jeweiligen Bewegungsrichtung.

Hüftinnen- und Außenrotatoren
Viele der Hüftrotatoren haben ihren Ursprung am Becken und setzen am großen Rollhügel des Oberschenkels an. Neben ihrer Funktion, das Gelenk zu bewegen, zentrieren sie den Hüftkopf in der Gelenkpfanne. Im Trainingsprogramm werden diese Muskelgruppen nicht explizit erwähnt, die ausgewählten Übungen sprechen diese jedoch ebenfalls an.

3 VON DER THEORIE ZUR PRAXIS

3.1 EINFÜHRUNG

3.1.1 Gesundheitliche Risiken und Kontraindikationen

Grundsätzlich stellt die Sporttherapie eine nebenwirkungsarme Therapieform dar und es bestehen kaum Kontraindikationen. Bei vorliegenden Begleiterkrankungen des Herz-Kreislaufsystems, systemischen Erkrankungen sowie vorausgegangenen Operationen stellt dieses Trainingsprogramm jedoch möglicherweise nicht die geeignete Therapieform dar. Wir empfehlen, mögliche Risiken durch Ihren Hausarzt abklären zu lassen.

3.1.2 Wichtige Informationen zur erfolgreichen Umsetzung des Heimtrainingsprogramms

Im folgenden Kapitel erläutern wir das notwendige Handwerkszeug für Ihr Heimtrainingsprogramm wie z. B. die Übungsanleitungen, das Trainingstagebuch und die Trainingsmittel.

Das Trainingsprogramm besteht aus drei Phasen. Die erste Phase dauert 3 Wochen. In diesen Wochen lernen Sie mit den anatomischen Besonderheiten der Hüftregion umzugehen, Bewegung präzise auszuführen und muskuläre Anspannung wahrzunehmen. Von Beginn an sollten Sie die bereitgestellten Trainingstagebücher nutzen, um das Training zu dokumentieren.

In den 5 Wochen der zweiten Phase werden Übungen eingesetzt, um die Gleichgewichtsfähigkeit und die Kraftausdauer der hüftumgebenden Muskulatur zu verbessern. Schließlich wird in den 4 Wochen der dritten Phase das Gleichgewichtstraining durch dynamische Übungsformen ergänzt und das Krafttraining für den Muskelaufbau deutlich intensiviert.

Übungsbezeichnungen und -anleitungen
Jede Übung ist durch eine Abkürzung gekennzeichnet und fortlaufend nummeriert. Die Abkürzung gibt Aufschluss darüber, welches primäre Ziel mit einer Übung verfolgt wird. **WM** steht für Wahrnehmungs- und Mobilisationsübungen, **GG** für Gleichgewichtsübungen und **K** weist auf Kräftigungsübungen hin.

!

WM Wahrnehmungs- und Mobilisationsübungen
GG Gleichgewichtsübungen
K Kräftigungsübungen

Jede Übung startet in einer definierten **Ausgangsstellung**. Aus dieser erfolgt eine **Aktion**, die einen Bewegungsauftrag beschreibt. Wenn Sie die Bewegung technisch richtig und mit der entsprechenden Trainingsintensität ausführen, sollte sich der beschriebene **Effekt** einstellen. Ist der Effekt für Sie nicht wahrnehmbar, sollten Sie Ihre Übungsausführung nochmals genau kontrollieren und gegebenenfalls korrigieren. Unter den meisten Übungen finden Sie in einem grauen Feld Hinweise, die Sie auf mögliche Fehler aufmerksam machen. Zudem werden hier Alternativen aufgezeigt, falls die Standardausführung Schwierigkeiten bereitet.

!

Die Übungsbeschreibung erfolgt jeweils über die
1. Angabe der Ausgangsstellung
2. Beschreibung der Aktion
3. Beschreibung des gewünschten Übungseffektes

Wenn Übungen mit dem linken und rechten Bein separat ausgeführt werden, beginnen Sie bitte stets mit dem linken Bein. Dies ist wichtig, da die Bilder und Beschreibungen die Übungsausführung mit dem linken Bein darstellen. Die Übung ist anschließend in gleicher Weise mit dem rechten Bein durchzuführen.

3.1.3 Ausgangsstellungen

Die mehr als 60 Übungen des Trainingsprogramms werden aus wenigen unterschiedlichen Ausgangsstellungen ausgeführt. Auf den nächsten Seiten werden diese erläutert.

3.1.3.1 Rückenlage, Seitenlage (siehe Seite 52)

Die Rückenlage und Seitenlage sind Ausgangsstellungen, die Ihnen eine sehr stabile Körperlage verleihen und gleichzeitig die Belastung für die Hüftgelenke klein halten. Übungen in dieser Ausgangslage eignen sich daher in besonderem Maße zur Mobilisation und zur Schulung der Wahrnehmung von muskulärer Spannung. Zum Einnehmen dieser Ausgangsstellungen kann eine Abstützhilfe wie z. B. ein Hocker oder Stuhl nützlich sein. Achten Sie darauf, dass die Abstützhilfe stets sicher steht.

Treten Beschwerden im Bereich des Nackens auf, können Sie diesen durch das Unterlagern des Kopfes mit einem Kissen oder einer Nackenrolle entgegenwirken. Bei den Übungen in Seitenlage empfiehlt es sich, auf der nicht betroffenen oder weniger schmerzhaften Seite (Hüfte) zu liegen. Neben dem Kissen unter dem Kopf kann ein »Abstandshalter« zwischen den Knien das Training für Sie angenehmer gestalten.

3.1.3.2 Sitz (siehe Seite 53)

Die Ausgangsstellung Sitz ist wie die liegenden Ausgangsstellungen durch eine geringe Belastung der Hüftgelenke gekennzeichnet. Für die Übungen im Sitz eignen sich am besten ein Hocker oder Stuhl mit ausreichender Stabilität. Setzen Sie sich auf den vorderen Bereich der Sitzfläche und achten Sie darauf, dass sich Ihr Gesäß bei senkrecht stehenden Unterschenkeln deutlich über den Knien befindet. Große Personen, eine stark eingeschränkte Hüftbeugung oder Knieprobleme erfordern eine zusätzliche Erhöhung der Sitzposition. Hierfür eignen sich ein festes Kissen oder ein Stabilisationstrainer. Für manche Personen kann auch die Tischkante eine geeignete Sitzmöglichkeit darstellen.

3.1.3.3 Stand – Parallelstand, Schrittstellung und Einbeinstand (siehe Seite 54 + 55)

In der Ausgangsstellung Stand besteht Bodenkontakt entweder mit beiden (Parallelstand und Schrittstellung) oder nur mit einem Fuß (Einbeinstand). Die Belastung für das Hüftgelenk ist hierbei höher als in den Ausgangsstellungen Sitz, Rücken- und Seitenlage. Vor allem im Einbeinstand nimmt die Belastung auf die Hüfte deutlich zu. So kann es sinnvoll sein, dass die Wiederholungen einer Übung im Einbeinstand wechselseitig durchgeführt werden, um eine kurzzeitige Entlastung für das Hüftgelenk des Standbeines zu erreichen.

Bei Dehn- und Kräftigungsübungen im Stand kann man sich bei Bedarf abstützen, um genügend Stabilität für die Übungen zu erlangen. Mögliche Stabilitätshilfen sind auf Seite 55 dargestellt.

Eine gute Wahrnehmung der Fußsohle und Beinachse spielt bei den Übungen im Stand eine wichtige Rolle. Dadurch schaffen Sie von »unten nach oben« die Basis für eine schonende Körperhaltung im Stehen.

3.1.4 Übungsmaterialien

Für das Heimtrainingsprogramm brauchen Sie verschiedene Materialien.

Neben den Übungsanleitungen und Trainingstagebüchern, die Sie als festen Bestandteil des Buches vor sich haben, benötigen Sie das Lesezeichen des Buches sowie verschiedene Trainingsmittel, die Sie beispielsweise im Orthopädiefachhandel beziehen können: Gymnastikmatte, Ball, Band, Gewichtsmanschette und Stabilisationstrainer. Sollte kein Stabilisationstrainer zur Verfügung stehen, so kann hierfür auch ein mehrfach gefaltetes, dickes Badetuch verwendet werden. Neben den Trainingsmitteln können Lagerungshilfen wie ein Kissen erforderlich werden, um einzelne Übungen problemloser zu gestalten. Die Übungsmaterialien sind auf den Seiten 52 – 55 dargestellt.

Rückenlage – Beine angestellt

- seitlicher Fußabstand hüftbreit
- Knie gebeugt
- Lendenwirbelsäule hat Kontakt zur Unterlage
- Kopf unterlagert

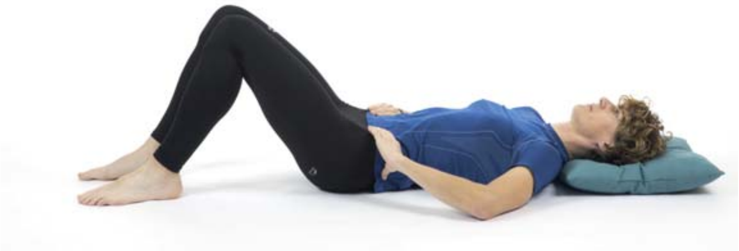

Rückenlage – Beine gestreckt

- seitlicher Fußabstand hüftbreit
- Knie gestreckt bzw. in leichter Beugung unterlagert
- Kopf unterlagert

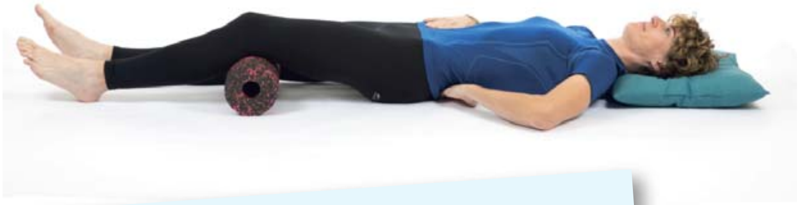

Materialien für Rückenlage und Seitenlage

- **alle Bilder:** *Kopfkissen*
- **Bild mittig:** *Knierolle oder Kissen zur Unterlagerung des Kniegelenks*
- **Bild unten:** *Stabilisationstrainer oder Kissen*

Seitenlage –
auf gesunder bzw. weniger betroffener Seite

- Knie und Hüfte gebeugt
- Kopf unterlagert

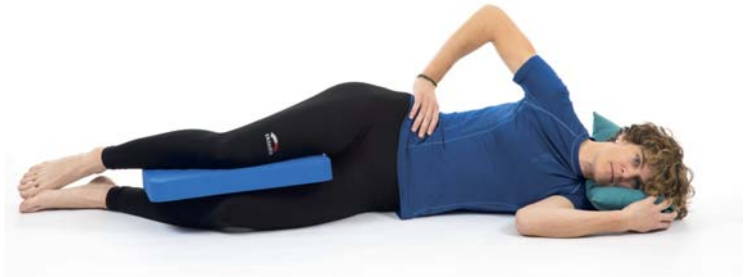

!

- Rückenlage – Beine gestreckt: Bei Schmerzen in Leiste oder Lendenwirbelsäule sollte das Knie auf der entsprechenden Seite unterlagert werden.
- Seitenlage: Bereitet die enge Knieposition Schmerzen, sollte ein Abstandhalter zwischen die Knie gelegt werden.

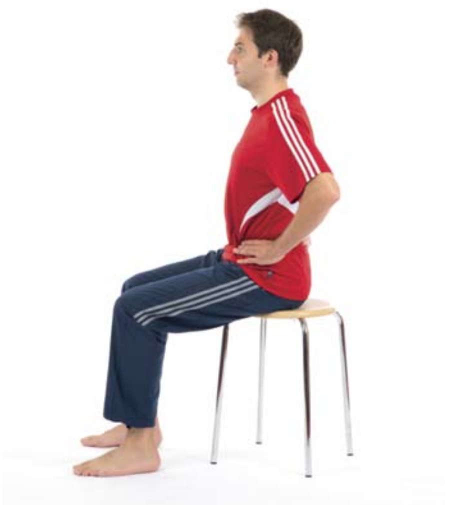

- Sitzfläche deutlich über Kniehöhe
- Gesäß auf vorderem Teil der Sitzfläche
- seitlicher Fußabstand übungsabhängig
- Fußspitzen zeigen nach vorne
- Knie senkrecht über Füßen
- Knie zeigen nach vorne
- Oberkörper aufrecht
- Blick nach vorne gerichtet

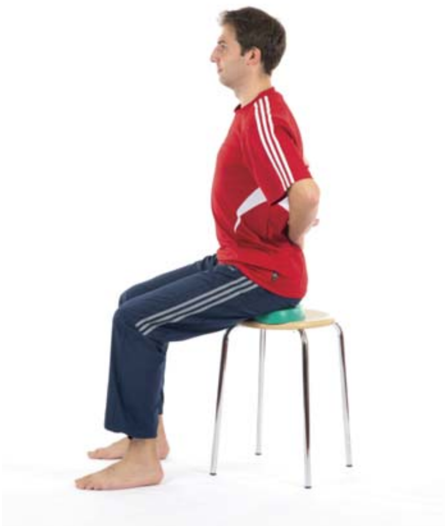

Variationsmöglichkeiten der Sitzhöhe:

- Bild oben: Hocker oder Stuhl
- Bild mittig: Sitzkissen oder Stabilisationstrainer zur Erhöhung der Sitzposition
- Bild unten: Tisch als deutlich erhöhte Sitzoption

Materialien für Sitz

Bild mittig: *Stabilisationstrainer als Sitzerhöhung*
Bild unten: *Gewichtsmanschette*

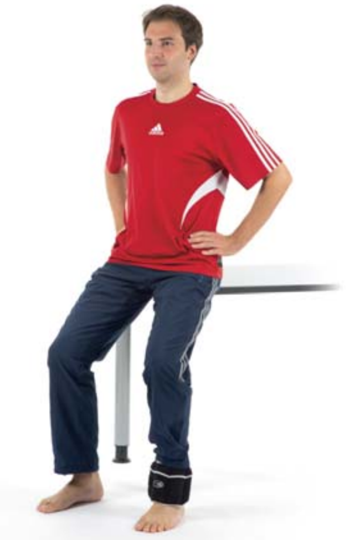

!

- Unbedingt eine stabile Sitzgelegenheit wählen, die nicht wegrutschen, wegrollen oder kippen kann.
- Sitzfläche ist bei senkrecht stehenden Unterschenkeln deutlich über Kniehöhe.
- Bei eingeschränkter Hüftbeugung oder Schmerzen in der Hüfte (z. B. Leiste) sollte die Sitzhöhe erhöht werden.

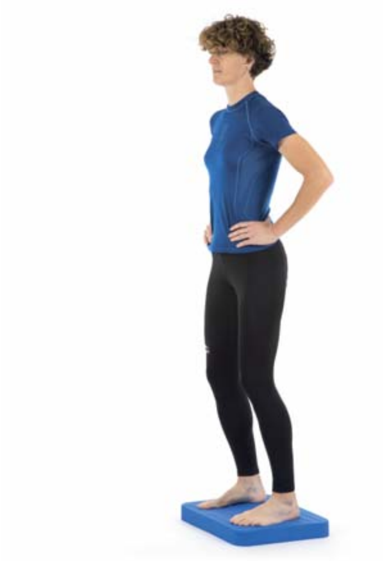

Parallelstand

- seitlicher Fußabstand übungsabhängig
- Fußspitzen zeigen nach vorne
- Füße gleichmäßig belastet
- Knie leicht gebeugt
- Knie zeigen nach vorne
- Oberkörper aufrecht
- Hände auf Beckenkamm
- Blick nach vorne gerichtet

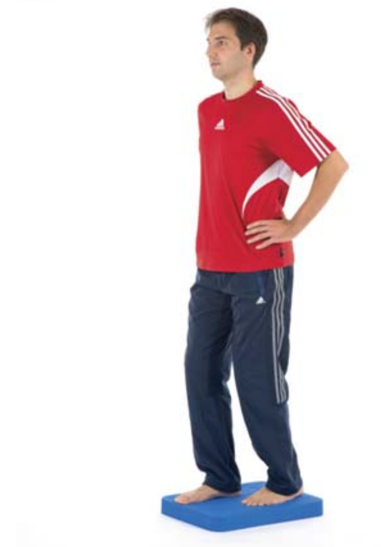

Schrittstellung

- Füße in Schrittstellung hintereinander
- seitlicher Fußabstand hüftbreit
- Füße gleichmäßig belastet
- Knie leicht gebeugt
- Knie und Fußspitzen zeigen nach vorne
- Hände auf Beckenkamm
- Blick nach vorne gerichtet

Materialien für Stand:

Alle Bilder: Stabilisationstrainer in unterschiedlichen Ausführungen

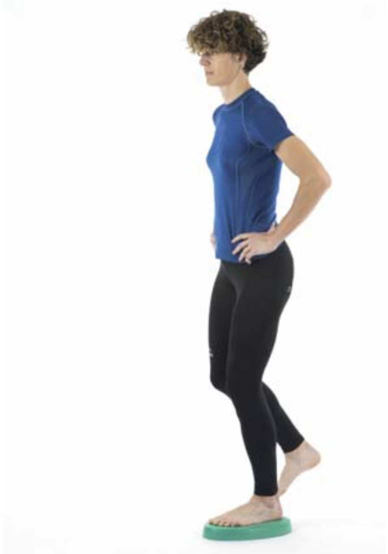

Einbeinstand

- Standbein-Knie leicht gebeugt
- Knie und Fußspitzen zeigen nach vorne
- Spielbein 2 cm vom Boden abgehoben
- Hände auf Beckenkamm
- Blick nach vorne
- Beine berühren sich nicht

!

- Abstützhilfen für den Stand bei Kräftigungs- und Dehnübungen sind auf der Folgeseite dargestellt.

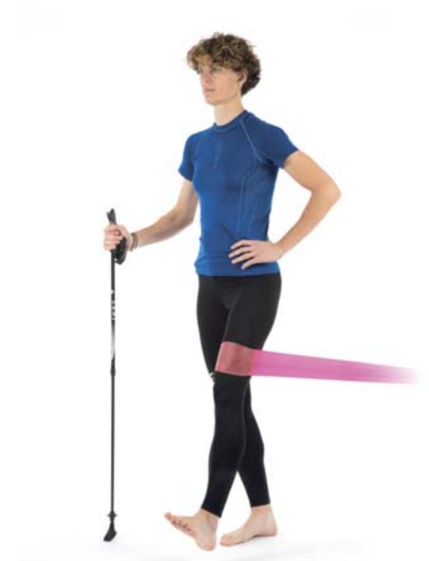

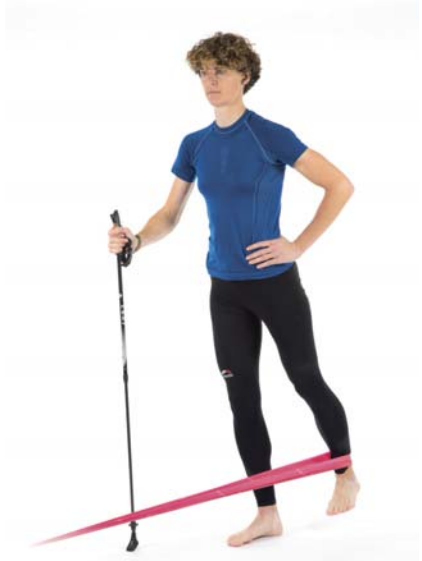

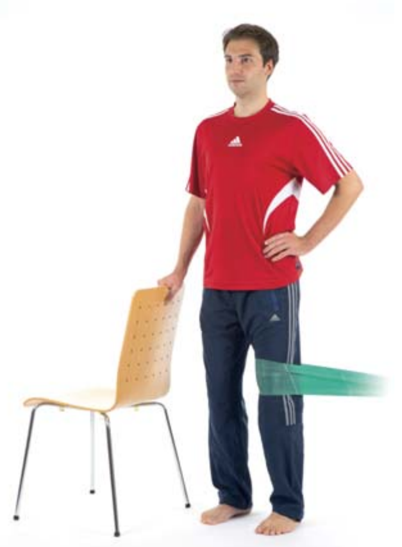

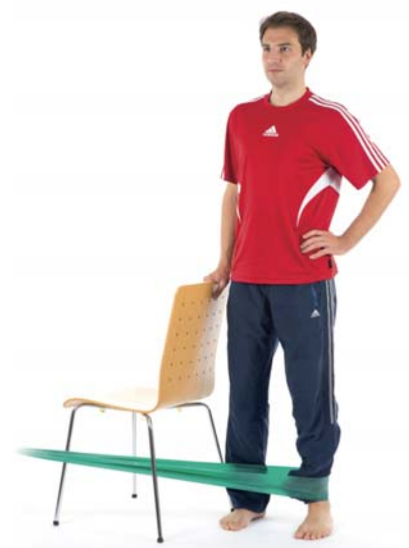

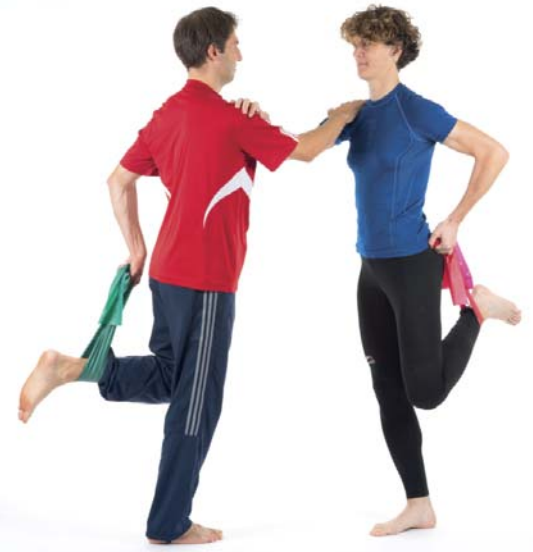

Abstützhilfen und mögliche Bandanlagen:

- **Bild oben links:** Stock, Bandanlage auf Kniehöhe
- **Bild oben rechts:** Stock, Bandanlage oberhalb des Knöchels
- **Bild mittig links:** Stuhl, Bandanlage auf Kniehöhe
- **Bild mittig rechts:** Stuhl, Bandanlage oberhalb des Knöchels
- **Bild unten:** Partner
- **Ohne Bild:** Wand, Schrank, Sims

DAS DIENSTLEISTUNGSZENTRUM
FÜR IHRE GESUNDHEIT

- Orthopädie-Technik
- Orthopädie-Schuhtechnik
- Sanitätshaus
- Rehabilitations-Technik
- Krankenpflege-Hilfsmittel

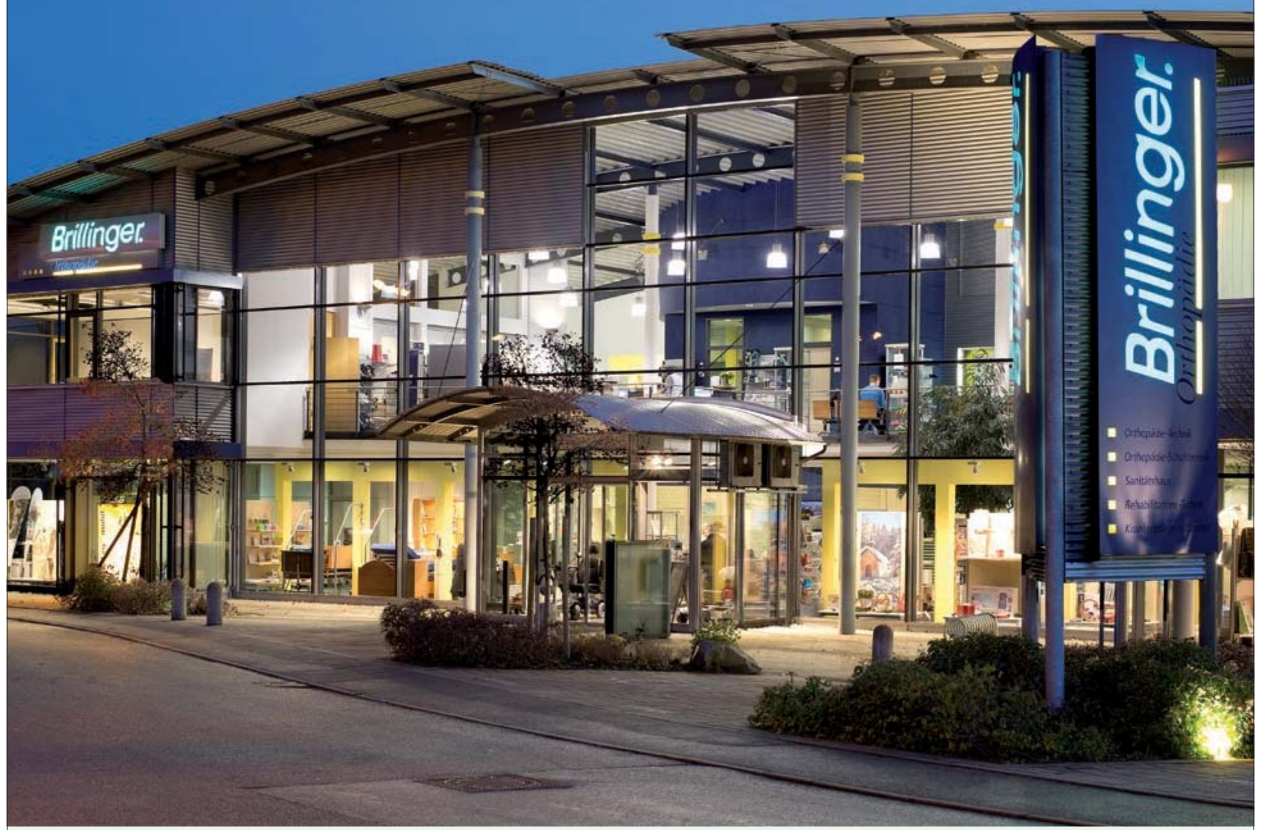

Brillinger.
Orthopädie
Das Gesundheitshaus

72070 Tübingen-West, Handwerker-Park 25
Tel. 0 70 71-41 04-0

72072 Tübingen, Karlstraße 6, Facharztzentrum
Tel. 0 70 71-40 03 10

72076 Tübingen, Hoppe-Seyler-Straße 6,
Gesundheitszentrum Tübingen (GZT)
Tel. 0 70 71-29-8 83 43

72764 Reutlingen, Obere Wässere 6–8
Tel. 0 71 21-5 14 90-0

72116 Mössingen, Karl-Jaggy-Straße 45
Tel. 0 74 73-13 21

72108 Rottenburg, Eugen-Bolz-Platz 3
Tel. 0 74 72-9 37 91 51

Weitere Infos unter: **www.brillinger.de**

3.1.5 Rahmenbedingungen für ein erfolgreiches Training

Bevor Sie nun mit dem Training beginnen möchten wir Sie auf einige wichtige Bedingungen für Ihr Training hinweisen:

!

1. **Nehmen Sie sich Zeit: Pro Einheit sollten Sie mit einer Übungsdauer von 40-50 Minuten rechnen.**

2. **Integrieren Sie das Training in Ihren Alltag: Versuchen Sie die beiden Trainingseinheiten fest in Ihren Wochenrhythmus einzubinden.**

3. **Verteilen Sie die Trainingseinheiten wenn möglich gleichmäßig auf die Woche. Auf diese Weise werden die Trainingsreize kontinuierlich gesetzt und Ihr Körper hat zudem zwischen den Einheiten genügend Zeit, sich zu erholen.**

4. **Beachten Sie die Trainingsvorgaben: Üben Sie kontrolliert und dosiert und versuchen Sie dabei die Angaben zur Bewegungsausführung, Übungsabfolge und Trainingsintensität korrekt umzusetzen. Halten Sie die Trainingsabfolge unbedingt ein, da die Übungen aufeinander aufbauen.**

5. **Achten Sie beim Üben auf Ihre Atmung: Atmen Sie stets gleichmäßig und vermeiden Sie Pressatmung. Pressatmung stellt eine zusätzliche Belastung für das Herz-Kreislaufsystem dar.**

6. **Berücksichtigen Sie Ihr Befinden und Ihre Schmerzen: Passen Sie Ihr Training entsprechend an bzw. verschieben Sie eine Trainingseinheit wenn notwendig.**

3.2 DAS TRAINING BEGINNT

3.2.1 Phase I (Woche 1 – 3)

Die ersten Wochen des Heimtrainingsprogramms enthalten vor allem Übungen mit sehr geringer Trainingsintensität. Hierbei ist eine sehr sorgfältige und kontrollierte Bewegungsdurchführung wichtig, um alle späteren Übungen technisch korrekt und effektiv ausführen zu können. Zur Bewegungskontrolle werden knöcherne Orientierungspunkte wie beispielsweise die vorderen Beckenpunkte eingesetzt. In Kapitel 2 finden Sie hierzu eine Übersicht sowie eine Anleitung wie Sie diese Orientierungspunkte, am besten auffinden können (siehe Seite 32, Tabelle 1).

Neben dem Erlernen der Bewegung dienen viele Übungen der Mobilisation des Hüftgelenks. Die Mobilisationsübungen finden in Ausgangsstellungen mit geringer Gelenkbelastung statt und fördern die Beweglichkeit des Hüftgelenks. Zudem wird die Durchblutung im Bereich des mobilisierten Gelenks angeregt und es kann zu einer kurzzeitigen Schmerzlinderung kommen. Die Verbesserung der Wahrnehmungsfähigkeit sowohl von muskulärer Spannung als auch der Kontaktfläche Fuß sind weitere Übungsschwerpunkte . Das achsgerechte Belasten der Beine wird im Sitz vorbereitet und anschließend im Stand fortgeführt (siehe Seite 36).

!

Obwohl die Übungen der ersten Phase nur wenig schweißtreibend sind, bilden sie die Grundlage aller weiterführenden Übungen. Bitte nehmen Sie diese Übungen ernst, dies wird Ihnen in den späteren Phasen des Programms zugutekommen.

Absolvieren Sie Ihr Trainingsprogramm 2 x pro Woche. In der ersten Phase sind die Übungseinheiten in Einheit 1 und Einheit 2 identisch.

AUGEPASST

WOCHE 1

1 2 3 4 5 6 7 8 9 10 11 12

ÜBUNGSMATERIALIEN

- Übungskatalog (Übungsanweisungen WM 1 – 10 + Trainingstagebuch Woche 1)
- Lesezeichen
- Hilfsmittel: Matte, Ball, Hocker/Stuhl, bei Bedarf Sitzerhöhung und Lagerungshilfen

AUSGANGSSTELLUNG

Rückenlage: WM 1 – 5
(1) Die Beine sind angestellt, d.h. in Hüfte und Knie gebeugt.
(2) Die Füße haben über die Ferse oder ganze Fußsohle Kontakt zum Boden.
(3) Die Arme liegen seitlich am Körper.

Sitz: WM 6 – 10
(1) Haltung aufrecht
(2) Sitzhöhe – mindestens kniehoch
(3) Sitzerhöhung – über Stabilisationstrainer, Kissen etc. möglich

ÜBUNGSINHALTE

Knöcherne Punkte nutzen – Orientierung, Kontrolle, Korrektur

Lendenwirbelsäule	durch Auflegen der Hand
Vorderer Beckenpunkt	durch Tasten mit dem Zeigefinger
Sitzbeinhöcker	durch spürbare Auflagepunkte im Sitz

Gelenk mobilisieren
Bewegungen langsam, kontrolliert (mit Hilfe von knöchernen Punkten) und nur mit minimalem Kraftaufwand 30 mal über 120 Sekunden im im schmerzfreien bzw. schmerzarmen Bereich ausführen.

Muskulatur wahrnehmen
(1) Muskuläre Spannung dosiert aufbauen
» 3 Sekunden halten » dann kontrolliert abbauen und entspannen
(2) 10 Wiederholungen mit langsamer Bewegungsausführung (mindestens 5 Sekunden)

WISSENSWERTES

Knochen und Orientierung .... Seite 31/32
Gelenk, -bewegung und -mobilisation .... Seite 31/35/37
Muskulatur und Wahrnehmung........................ Seite 33/34/37
Dokumentation .... Seite 45

WM 1	MOBILISATION
WIEDERHOLUNGSZAHL: 30	ÜBUNGSDAUER: MIND. 120 SEK. GESAMT

Ausgangsstellung: Rückenlage (Beine angestellt)

- seitlicher Fußabstand hüftbreit
- Knie gebeugt
- Hände auf Beckenkamm
- Zeigefinger auf vorderem Beckenpunkt

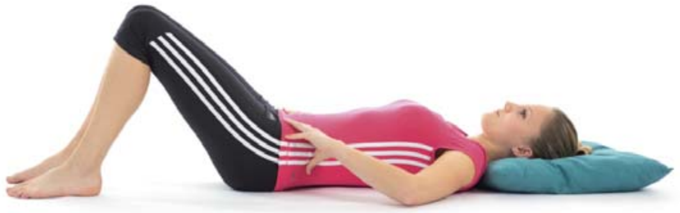

Aktion 1:
- Becken nach hinten rollen, bis gesamte Lendenwirbelsäule Kontakt zur Unterlage hat (Bauch einziehen)

» **Effekt 1:**
- Beckenpunkte bewegen sich nach unten

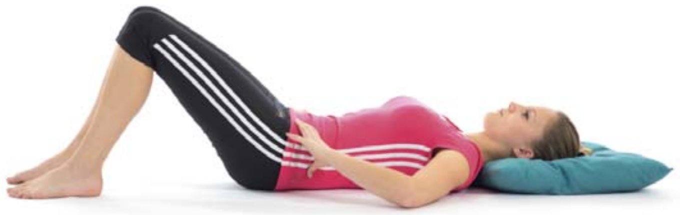

Aktion 2:
- Becken langsam nach vorne kippen, bis leichtes Hohlkreuz entsteht

» **Effekt 2:**
- Beckenpunkte bewegen sich nach oben

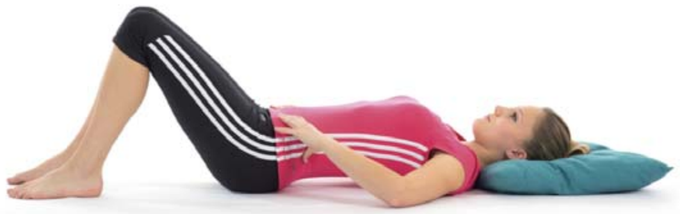

!

- Gesäß bleibt während der Übung in Kontakt zu der Unterlage.
- Beckenbewegung ist mit Händen und Zeigefinger wahrnehmbar.

WM 2	WAHRNEHMUNG
WIEDERHOLUNGSZAHL: 10 JE SEITE	ÜBUNGSDAUER JE WIEDERHOLUNG: MIND. 5 SEK.

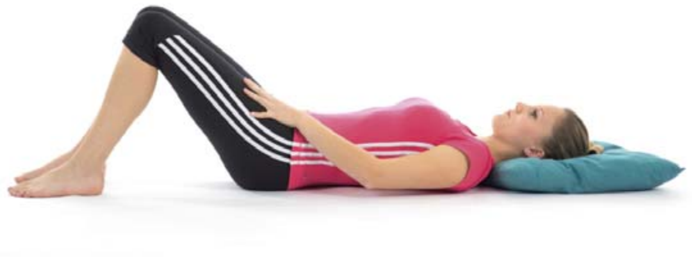

Ausgangsstellung: Rückenlage (Beine angestellt)

- seitlicher Fußabstand 10 cm
- Knie gebeugt
- Lendenwirbelsäule hat Kontakt zur Unterlage
- linke Hand auf Oberschenkel
- rechte Hand auf Beckenkamm

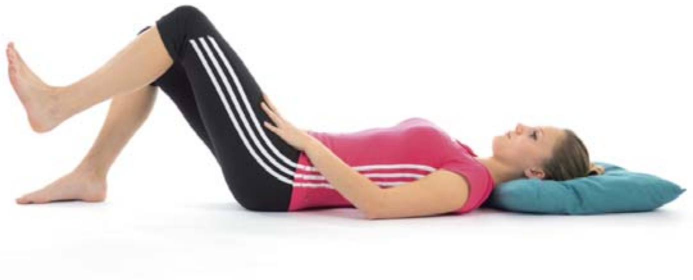

Aktion 1:
- linkes Bein etwas anheben
- Position 3 Sekunden halten

» Effekt 1:
- Muskuläre Anspannung des linken Oberschenkel nimmt zu (mit linker Hand spürbar)

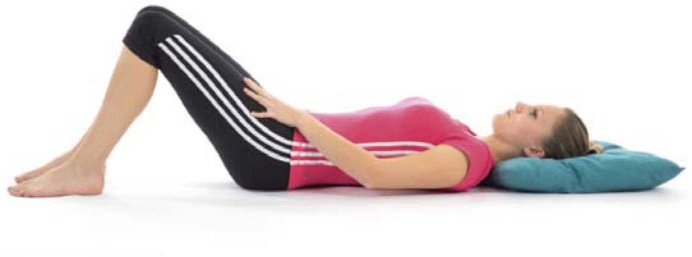

Aktion 2:
- linkes Bein langsam wieder absenken und Fuß abstellen
- Position 3 Sekunden halten

» Effekt 2:
- Muskuläre Anspannung des linken Oberschenkel nimmt wieder ab (mit linker Hand spürbar)

!

- Lendenwirbelsäule bleibt während der Übung in Kontakt zu der Unterlage.
- Nach vorgegebener Wiederholungszahl Seite wechseln.

WM 3	MOBILISATION
WIEDERHOLUNGSZAHL: 30	ÜBUNGSDAUER: MIND. 120 SEK. GESAMT

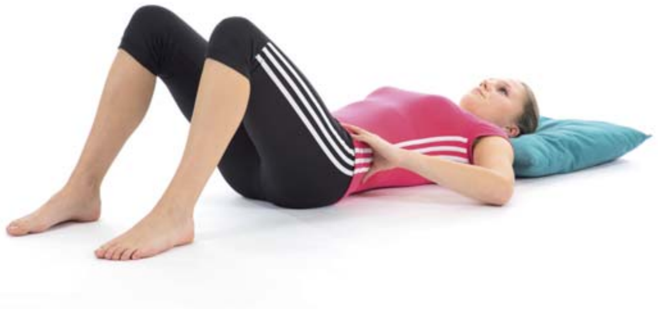

Ausgangsstellung: Rückenlage (Beine angestellt)

- seitlicher Fußabstand mindestens hüftbreit
- Knie gebeugt
- Lendenwirbelsäule und Gesäß haben Kontakt zur Unterlage
- Hände auf Beckenkamm

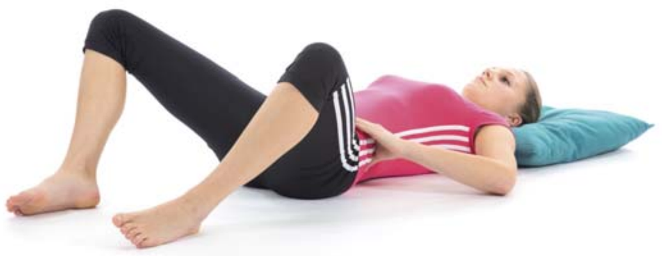

Aktion 1:
- beide Knie langsam nach außen bewegen

» Effekt 1:
- Abstand zwischen den Knien wird größer
- Fußinnenseite hebt ab

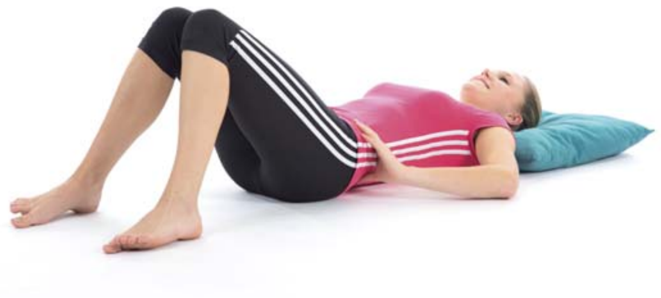

Aktion 2:
- beide Knie langsam soweit wie möglich nach innen bewegen

» Effekt 2:
- Abstand zwischen den Knien wird kleiner
- Fußaußenseite hebt ab

!

- Bewegung nicht schwunghaft ausführen oder mit Kraft nachdrücken.
- Lendenwirbelsäule und Gesäß bleiben während der Übung in Kontakt zu der Unterlage.

WM 4	WAHRNEHMUNG
WIEDERHOLUNGSZAHL: 10	ÜBUNGSDAUER JE WIEDERHOLUNG: MIND. 5 SEK.

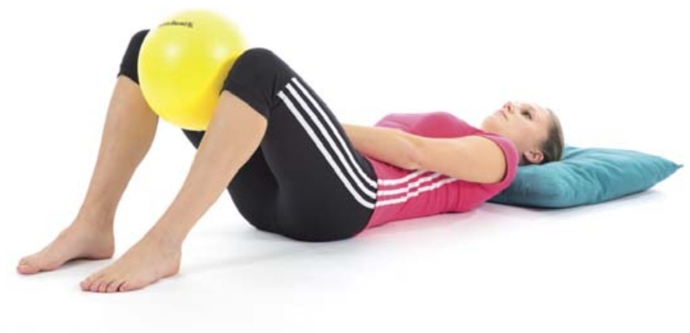

Ausgangsstellung: Rückenlage (Beine angestellt)

- seitlicher Fußabstand 10 cm
- Knie gebeugt
- Ball hängt zwischen den Knien
- Lendenwirbelsäule hat Kontakt zur Unterlage
- linke Hand auf rechter Oberschenkelinnenseite

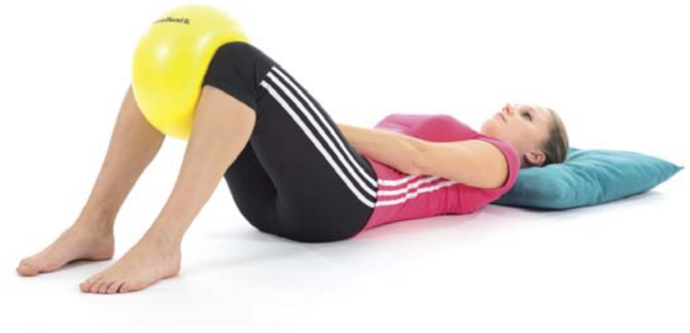

Aktion 1:
- beide Knie langsam mit wenig Kraft gegen den Ball drücken
- Position 3 Sekunden halten

» Effekt 1:
- Muskuläre Anspannung der Oberschenkelinnenseiten nimmt zu (mit linker Hand spürbar)

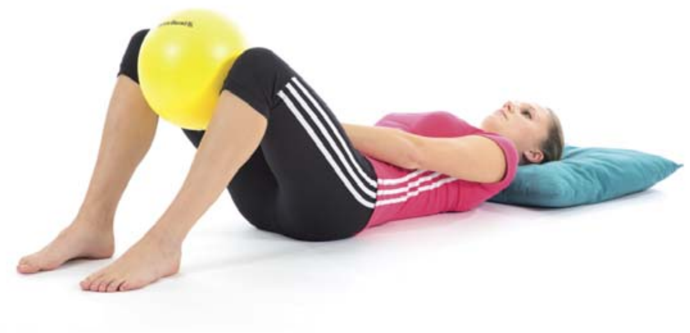

Aktion 2:
- Druck gegen Ball langsam verringern

» Effekt 2:
- Muskuläre Anspannung der Oberschenkelinnenseiten nimmt ab (mit linker Hand spürbar)

!

- Lendenwirbelsäule bleibt während der Übung in Kontakt zu der Unterlage.

WM 5	WAHRNEHMUNG
WIEDERHOLUNGSZAHL: 10 JE SEITE	ÜBUNGSDAUER JE WIEDERHOLUNG: MIND. 5 SEK.

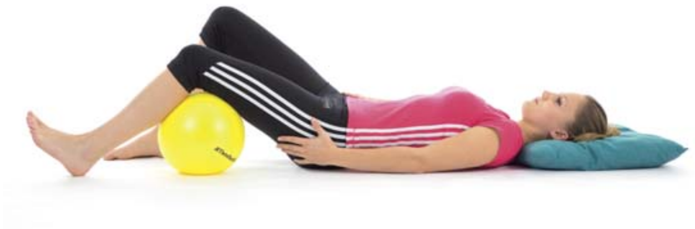

Ausgangsstellung: Rückenlage (Beine angestellt)

- seitlicher Fußabstand hüftbreit
- linkes Knie liegt auf Ball und zeigt zur Decke
- rechtes Knie gebeugt
- Lendenwirbelsäule hat Kontakt zur Unterlage
- linke Hand auf linker Oberschenkelrückseite/Gesäß

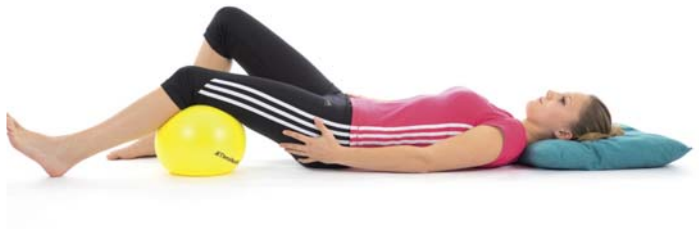

Aktion 1:
- linkes Knie langsam mit wenig Kraft gegen den Ball drücken
- Position 3 Sekunden halten

» Effekt 1:
- muskuläre Anspannung der linken Oberschenkelrückseite nimmt zu (mit linker Hand spürbar)

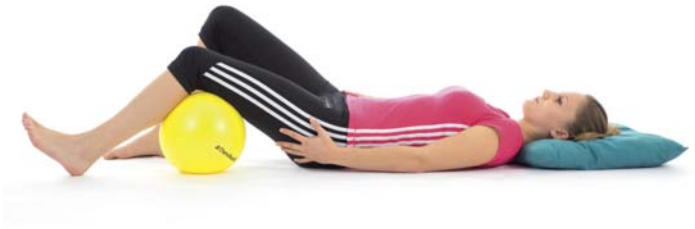

Aktion 2:
- Druck gegen Ball langsam verringern

» Effekt 3:
- Muskuläre Anspannung der linken Oberschenkelrückseite nimmt ab (mit linker Hand spürbar)

!

- Lendenwirbelsäule bleibt während der Übung in Kontakt zu der Unterlage.
- Fersen bleiben während der Übung in Kontakt zu der Unterlage.
- Bereitet der Ball in der Kniekehle Schmerzen, sollte er etwas oberhalb des Knies positioniert werden.
- Nach vorgegebener Wiederholungszahl Seite wechseln.

WM 6	MOBILISATION
WIEDERHOLUNGSZAHL: 30	ÜBUNGSDAUER: MIND. 120 SEK. GESAMT

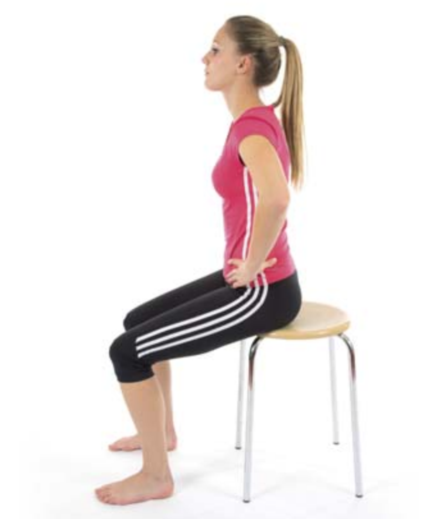

Ausgangsstellung: Sitz

- seitlicher Fußabstand hüftbreit
- beide Sitzbeinhöcker spürbar
- Hände auf Beckenkamm
- Zeigefinger auf vorderem Beckenpunkt

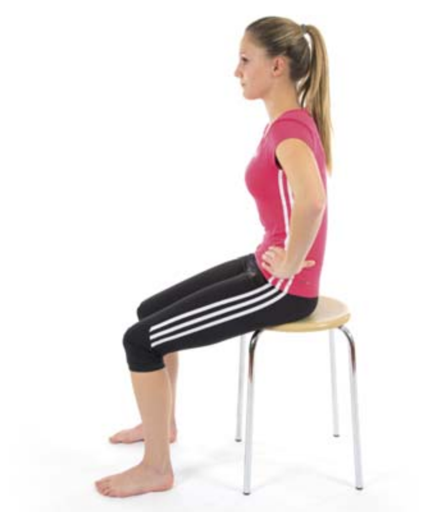

Aktion 1:
- Becken langsam nach hinten rollen, bis sich Lendenwirbelsäule leicht nach hinten wölbt (Bauch einziehen)

» Effekt 1:
- Beckenpunkte bewegen sich nach hinten
- Sitzbeinhöcker bei Rollbewegung spürbar

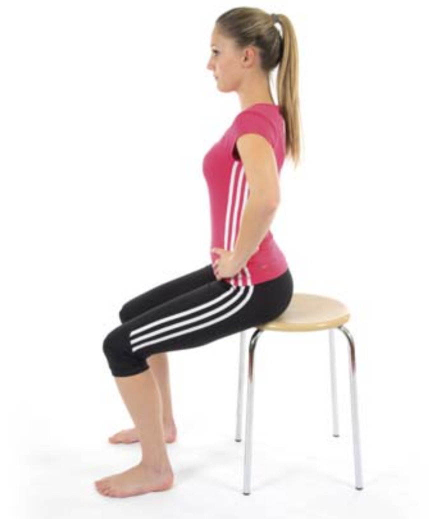

Aktion 2:
- Becken langsam nach vorne kippen, bis ein leichtes Hohlkreuz entsteht

» Effekt 2:
- Beckenpunkte bewegen sich nach vorne
- Sitzbeinhöcker bei Rollbewegung spürbar

!

- Bei der Bewegung rollt das Becken über die Sitzbeinhöcker vor und zurück.
- Beckenbewegung ist mit den Händen und Zeigefingern wahrnehmbar.

WM 7	WAHRNEHMUNG
WIEDERHOLUNGSZAHL: 10	ÜBUNGSDAUER JE WIEDERHOLUNG: MIND. 5 SEK.

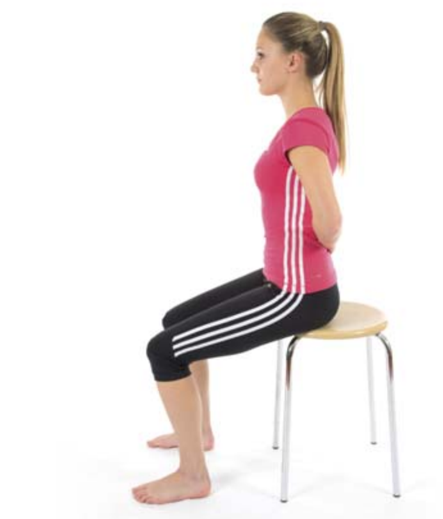

Ausgangsstellung: Sitz

- aufrechte Haltung
- Blick nach vorne gerichtet
- seitlicher Fußabstand mindestens hüftbreit
- Sitzbeinhöcker spürbar
- Knie senkrecht über Fußspitzen
- linke Hand auf Lendenwirbelsäule

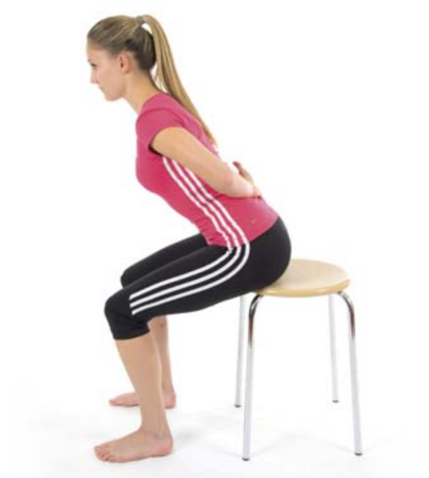

Aktion 1:
- Becken, Oberkörper und Kopf als Ganzes langsam nach vorne neigen (Wirbelsäule bleibt in sich gerade)
- Position 3 Sekunden halten

» Effekt 1:
- Hand und Lendenwirbelsäule bewegen sich nach vorne
- Blick richtet sich zunehmend nach vorne unten
- Belastung der Sitzbeinhöcker nimmt ab

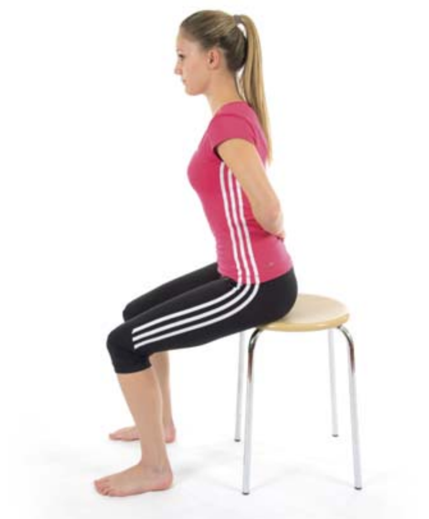

Aktion 2:
- Becken, Oberkörper und Kopf als Ganzes langsam zurück in die Ausgangsstellung führen (Wirbelsäule bleibt in sich gerade)

» Effekt 2:
- Hand und Lendenwirbelsäule bewegen sich nach hinten
- Blick richtet sich zunehmend nach vorne
- Belastung der Sitzbeinhöcker nimmt zu

!

- Während der Übung findet zwischen Becken und Oberkörper keine Bewegung statt – Kontrolle erfolgt über die Hand auf der Lendenwirbelsäule.

WM 8	WAHRNEHMUNG
WIEDERHOLUNGSZAHL: 10	ÜBUNGSDAUER JE WIEDERHOLUNG: MIND. 5 SEK.

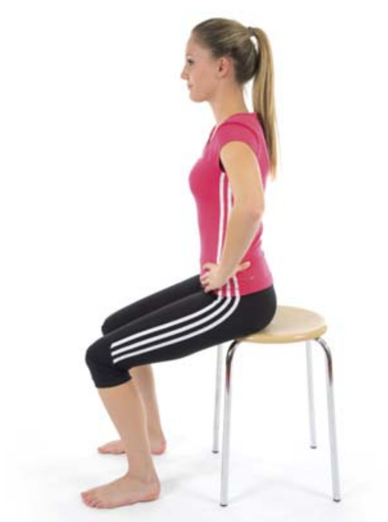

Ausgangsstellung: Sitz

- seitlicher Fußabstand hüftbreit
- Füße etwas nach hinten versetzt
- Knie senkrecht über Fußspitzen
- Hände auf Beckenkamm

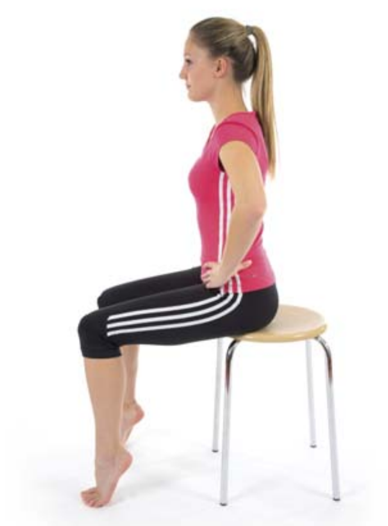

Aktion 1:
- beide Fersen deutlich vom Boden abheben
- Fußspitzen behalten Kontakt zum Boden
- Position 3 Sekunden halten

» Effekt 1:
- Knie und Oberschenkel bewegen sich nach oben

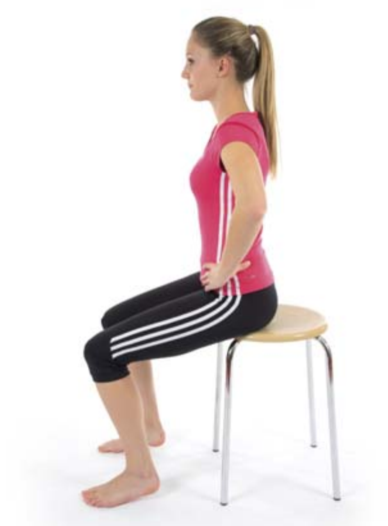

Aktion 2:
- beide Fersen wieder zum Boden absenken

» Effekt 2:
- Knie und Oberschenkel bewegen sich nach unten

!

- Während der Übung findet am Becken keine Bewegung statt – Kontrolle erfolgt über die Hand am Beckenkamm.
- Knie bewegen sich nicht nach innen und außen, sondern nur nach oben und unten.

WM 9	MOBILISATION
WIEDERHOLUNGSZAHL: 30	ÜBUNGSDAUER: MIND. 120 SEK. GESAMT

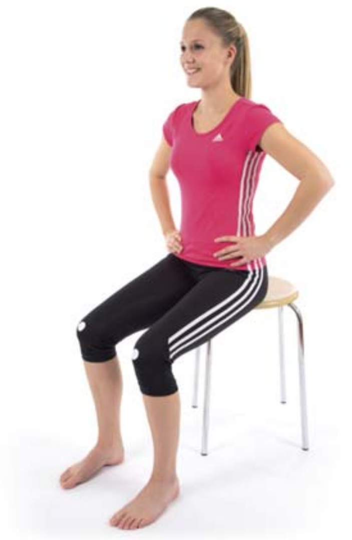

Ausgangsstellung: Sitz

- seitlicher Fußabstand hüftbreit
- Knie senkrecht über Füßen
- Hände auf Beckenkamm

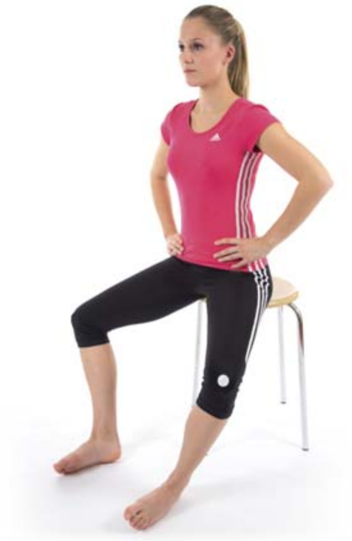

Aktion 1:
- beide Knie langsam nach außen bewegen

» Effekt 1:
- Abstand der Knie wird größer
- Fußinnenseite hebt etwas ab

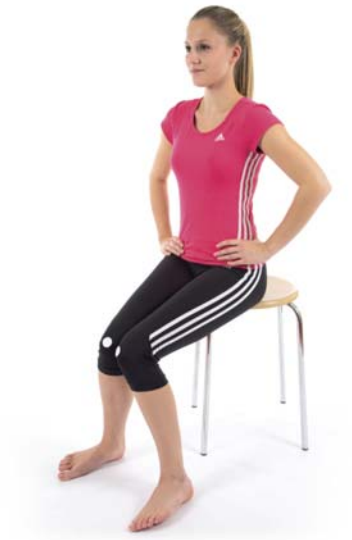

Aktion 2:
- beide Knie langsam so weit wie möglich nach innen bewegen

» Effekt 2:
- Abstand der Knie wird kleiner
- Fußaußenseite hebt etwas ab

!

- Während der Übung findet am Becken keine Bewegung statt – Kontrolle erfolgt über die Hand am Beckenkamm.
- Bewegung nicht schwunghaft ausführen oder mit Kraft nachdrücken.

WM 10	MOBILISATION
WIEDERHOLUNGSZAHL: 30	ÜBUNGSDAUER: MIND. 120 SEK. GESAMT

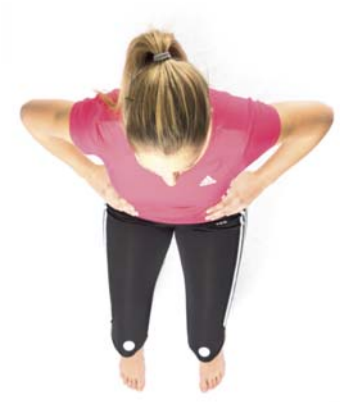
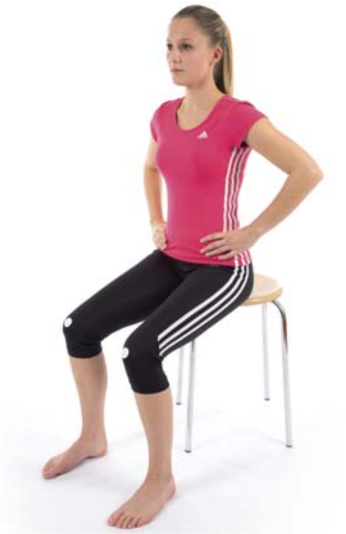

Ausgangsstellung: Sitz

- seitlicher Fußabstand hüftbreit
- Knie senkrecht über Fuß
- Sitzbeinhöcker nachspüren
- Hand liegt auf Beckenkamm
- Zeigefinger auf vorderem Beckenpunkt

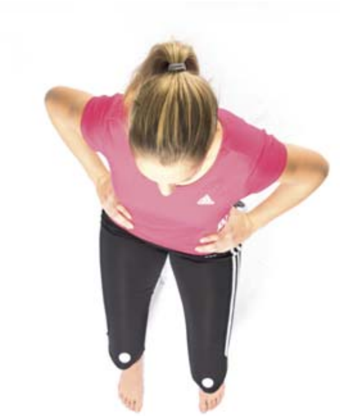
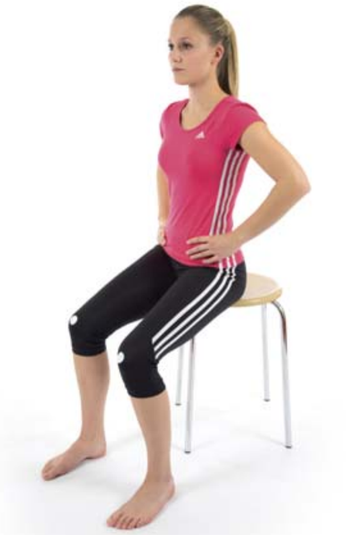

Aktion 1:

- linkes Knie langsam nach vorne schieben, gleichzeitig rechtes Knie langsam nach hinten ziehen
- Füße bleiben stehen

» Effekt 1:

- linkes Knie und linker Sitzbeinhöcker bewegen sich nach vorne
- rechtes Knie und rechter Sitzbeinhöcker bewegen sich nach hinten

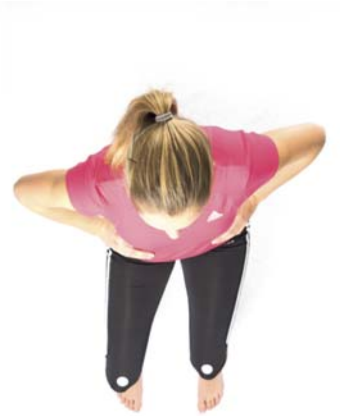
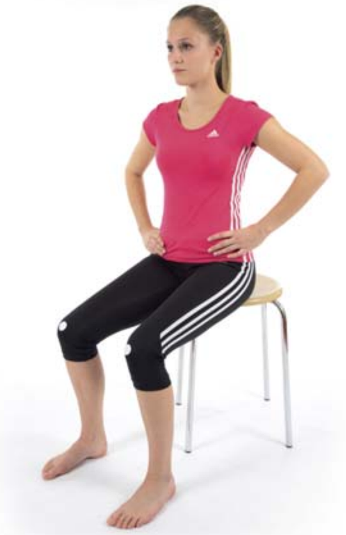

Aktion 2:

- rechtes Knie langsam nach vorne schieben, gleichzeitig linkes Knie langsam nach hinten ziehen
- Füße bleiben stehen

» Effekt 2:

- rechtes Knie und rechter Sitzbeinhöcker bewegen sich nach vorne
- linkes Knie und linker Sitzbeinhöcker bewegen sich nach hinten

!

- Bewegung nicht schwunghaft ausführen oder mit Kraft nachdrücken.

TRAININGSTAGEBUCH WOCHE 1 — **TRAININGSEINHEIT 1**

BEFINDEN/SCHMERZ VOR DEM TRAINING

Schmerzintensität	Lokalisation	Mögliche Ursache
kein 0 1 2 3 4 5 6 7 8 9 10 extrem		

Übung	Trainingsvorgabe Durchgang	Wiederholungen	Check ☒	Schmerz Intensität 1 – 10	Schmerz Lokalisation
WM 1	1	30	☐		
WM 2	1	10	L R		
WM 3	1	30	☐		
WM 4	1	10	☐		
WM 5	1	10	L R		
WM 6	1	30	☐		
WM 7	1	10	☐		
WM 8	1	10	☐		
WM 9	1	30	☐		
WM 10	1	30	☐		

TRAININGSTAGEBUCH WOCHE 1 — **TRAININGSEINHEIT 2**

BEFINDEN/SCHMERZ VOR DEM TRAINING

Schmerzintensität	Lokalisation	Mögliche Ursache
kein 0 1 2 3 4 5 6 7 8 9 10 extrem		

Übung	Trainingsvorgabe Durchgang	Wiederholungen	Check ☒	Schmerz Intensität 1 – 10	Schmerz Lokalisation
WM 1	1	30	☐		
WM 2	1	10	L R		
WM 3	1	30	☐		
WM 4	1	10	☐		
WM 5	1	10	L R		
WM 6	1	30	☐		
WM 7	1	10	☐		
WM 8	1	10	☐		
WM 9	1	30	☐		
WM 10	1	30	☐		

Tabelle 9 Muskelgruppen der Hüfte – Ausgangsstellungen und Übungsnummern.

Muskulatur	Hüftstrecker	Hüftbeuger	Hüftabspreizer	Adduktoren
Kräftigungsübungen in Rückenlage	K 1a/b	K 2a/b	K 3	K 4
Kräftigungsübungen im Stand und Sitz	K 5a/b	K 6a/b	K 7	K 8

Hintergrund für dieses Vorgehen ist, dass nicht jeder mit allen Ausgangsstellungen in gleichem Maße und somit mit den unterschiedlichen Übungen zu Recht kommt. Die Wahlmöglichkeit soll es Ihnen ermöglichen, Ihr Training stets in optimaler Art und Weise durchzuführen.

Kriterien für die Auswahl einer Übung sind:

- der Schmerz (falls vorhanden) sollte möglichst gering sein
- die empfundene Anstrengung sollte am Ende eines Durchgangs mindestens »anstrengend« oder »sehr anstrengend« empfunden werden
- die technische Übungsausführung sollte Ihnen keine Schwierigkeiten bereiten

!

- **Zwischen den 2 Trainingseinheiten/Woche sollte mindestens 1 Tag Pause liegen.**
- **Die Übungen der beiden Trainingseinheiten sind ab Woche 6 nicht mehr identisch.**
- **Sie sollten Ihr Training immer mit einem kurzen Aufwärmprogramm beginnen (siehe Lesezeichen).**
- **Im Anschluß daran folgt das Gleichgewichtstraining und danach die Kräftigung.**

ÜBUNGSMATERIALIEN

- Übungskatalog (Übungsanweisungen GG 1 – 4, K 1 – 4 + Trainingstagebuch Woche 4)
- Lesezeichen
- Hilfsmittel: Matte, Band, Ball; bei Bedarf Lagerungs- und Abstützhilfen

AUSGANGSSTELLUNG

Stand: GG 1 – 4
In allen 4 Positionen zeigen die Füße bei leicht gebeugtem Knie nach vorne.
Die Hände liegen auf dem Beckenkamm.

Rückenlage: K 1 – 4
Die Kräftigungsübungen werden zuerst mit angestellten Beinen ausgeführt.
K1 und K2 können dann zur Steigerung der Intensität mit vermehrt gestrecktem Bein absolviert werden.

ÜBUNGSINHALTE

Training statisches Gleichgewicht
(1) Wahlweise in den Varianten A-D
(2) Je Durchgang 6 Wiederholungen über jeweils 15 Sekunden

Kraftausdauer in Rückenlage
(1) 20 Wiederholungen, 2 Durchgänge
(2) Die Pause (ca. 60 Sekunden) zwischen dem ersten und zweiten Durchgang kann für Mobilisationsübungen und/oder zur Trainingsdokumentation genutzt werden.
(3) Bei Übungen mit einbeiniger Ausführung ist eine Pause nicht erforderlich.

WISSENSWERTES

Training .... Seite 36/37
Kraft und Kraftausdauer .... Seite 37/38
Trainingsintensität .... Seite 39 – 41
Gleichgewicht statisch .... Seite 42 – 44
Dokumentation .... Seite 45

GG 3 | GLEICHGEWICHT TANDEMSTAND

WIEDERHOLUNGSZAHL: 6 JE SEITE | ÜBUNGSDAUER: POSITION 15 SEK. HALTEN

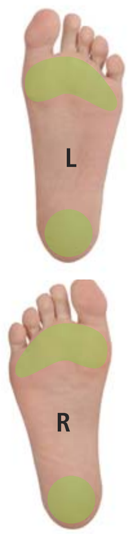

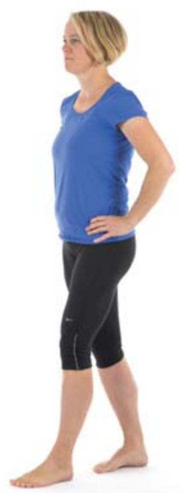

Ausgangsstellung: Tandemstand

- linker Fuß steht (wenn möglich) direkt vor rechtem Fuß
- gleichmäßige Belastung der Füße
- Knie leicht gebeugt
- Knie und Fußspitzen zeigen nach vorne
- Hände liegen auf Beckenkamm
- Blick nach vorne

Wählen Sie aus! Variante A, B, C oder D

VARIANTEN A – D

A Augen offen
Standfläche stabil

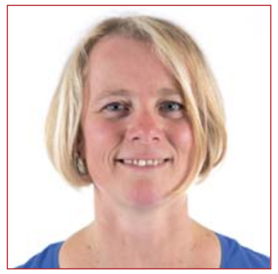

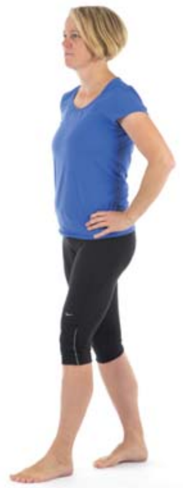

B Augen geschlossen
Standfläche stabil

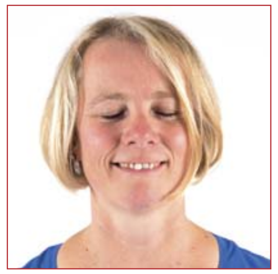

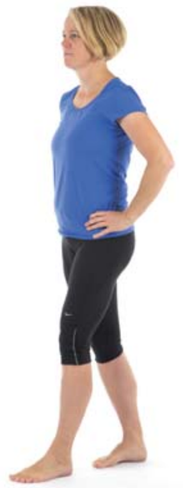

C Augen offen
Standfläche instabil

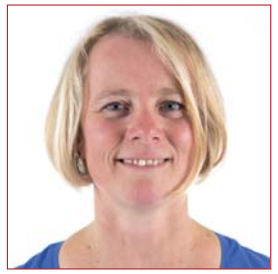

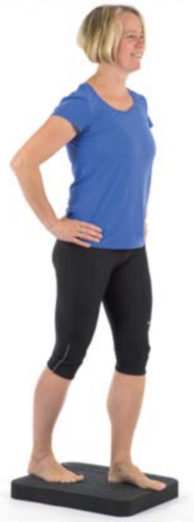

D Augen geschlossen
Standfläche instabil

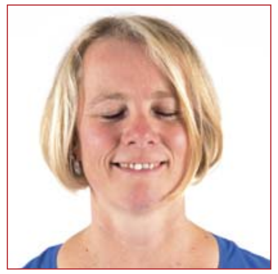

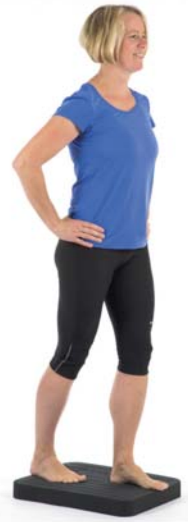

!

- Nach 15 Sekunden Haltedauer jeweils Fußposition wechseln.
- Knie bleiben leicht gebeugt, Oberkörper so ruhig wie möglich halten.
- Keine Ausweichbewegungen mit den Armen zulassen.

GG 4 | GLEICHGEWICHT EINBEINSTAND

WIEDERHOLUNGSZAHL: 6 JE SEITE | ÜBUNGSDAUER: POSITION 15 SEK. HALTEN

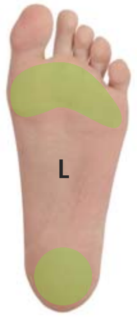

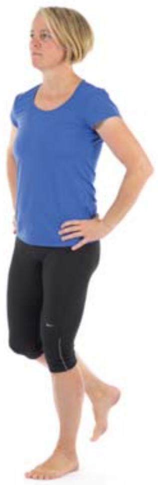

Ausgangsstellung: Einbeinstand

- Standbein links
- linkes Knie leicht gebeugt
- Knie und Fußspitzen zeigen nach vorne
- rechtes Bein 2 cm vom Boden abgehoben
- Hände auf Beckenkamm
- Blick nach vorne
- linkes und rechtes Bein berühren sich nicht

Wählen Sie aus! Variante A, B, C oder D

VARIANTEN A – D

A Augen offen
Standfläche stabil

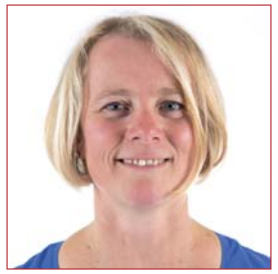

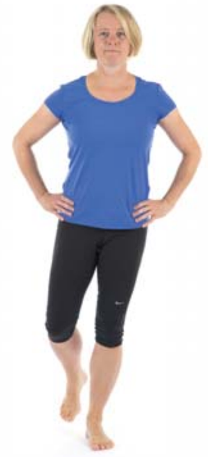

B Augen geschlossen
Standfläche stabil

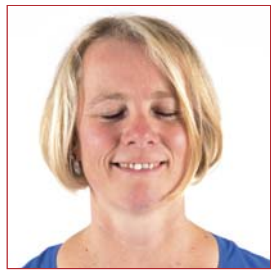

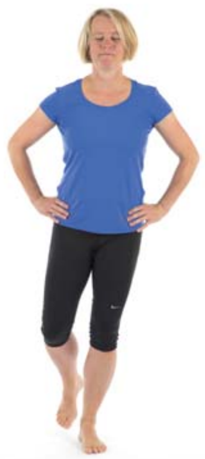

C Augen offen
Standfläche instabil

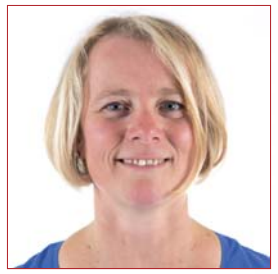

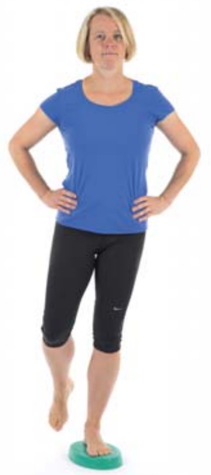

D Augen geschlossen
Standfläche instabil

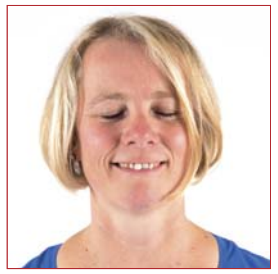

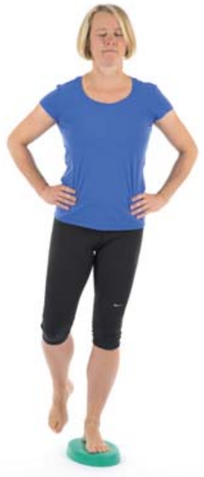

!

- Nach 15 Sekunden Haltedauer jeweils Fußposition wechseln.
- Knie des Standbeins bleibt leicht gebeugt, Oberkörper und Spielbein so ruhig wie möglich halten.
- Keine Ausweichbewegungen mit den Armen zulassen.
- Kontakt des Spielbeins zum Standbein vermeiden.
- Wenn Ihnen die Übung sehr schwer fällt, können Sie die Zehen des Spielbeins als Stabilisierungshilfe auf den Boden aufsetzen.

K 1a | **KRÄFTIGUNG HÜFTSTRECKER** (EINFACHE VARIANTE)

WIEDERHOLUNGSZAHL: 20 | DURCHGÄNGE: 2

Wählen Sie aus! Übung K 1a oder b

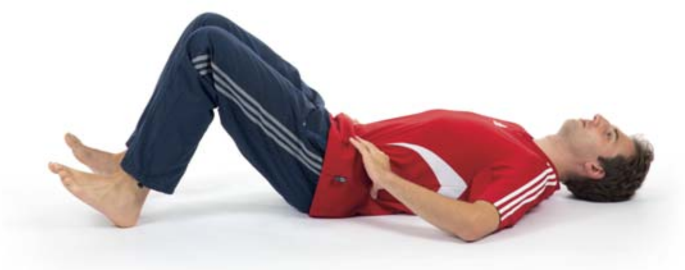

Ausgangsstellung:
Rückenlage (Beine angestellt)

- seitlicher Fußabstand hüftbreit
- Knie gebeugt
- Lendenwirbelsäule hat Kontakt zu der Unterlage
- Hände auf Beckenkamm
- Zeigefinger auf vorderem Beckenpunkt

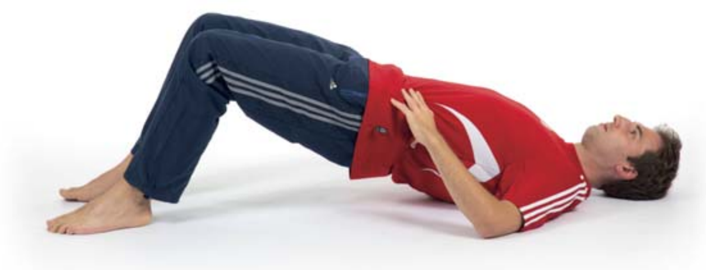

Aktion 1:
- Gesäß mit eingezogenem Bauch abheben
- Position 1 – 2 Sekunden halten

» **Effekt 1:**
- muskuläre Anspannung an Rücken, Gesäß und Oberschenkel nimmt zu

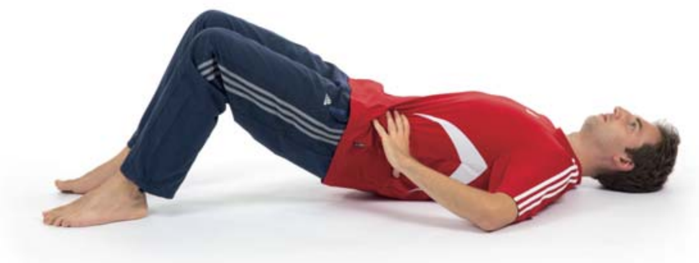

Aktion 2:
- Gesäß langsam bis kurz über Boden absenken

» **Effekt 2:**
- muskuläre Anspannung nimmt ab
- Lendenwirbelsäule hat wieder Kontakt zur Unterlage, das Gesäß nicht!

!

- Bei der Übung kein Hohlkreuz zulassen.
- Das Gesäß kann kurzzeitig abgelegt werden, wenn eine Pause erforderlich ist.
- Beide vordere Beckenpunkte befinden sich während der Übung auf gleicher Höhe. Kein einseitiges Absinken des Beckens zulassen – Kontrolle über Zeigefinger.

K 1b | **KRÄFTIGUNG HÜFTSTRECKER** (INTENSIVE VARIANTE)

WIEDERHOLUNGSZAHL: 20 | DURCHGÄNGE: 2

Wählen Sie aus! Übung K 1a oder b

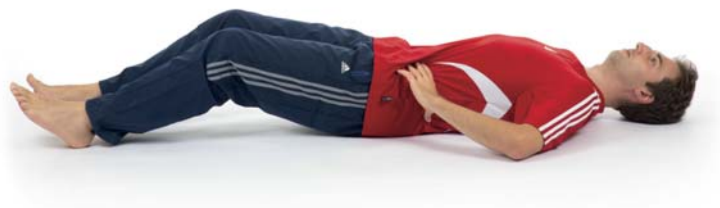

Ausgangsstellung: Rückenlage

- seitlicher Fußabstand hüftbreit
- Knie nur leicht gebeugt
- Lendenwirbelsäule hat Kontakt zu der Unterlage
- Hände auf Beckenkamm
- Zeigefinger auf vorderem Beckenpunkt

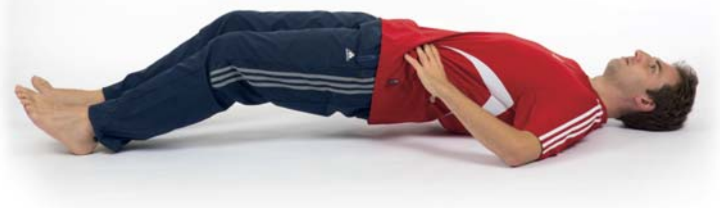

Aktion 1:
- Gesäß mit eingezogenem Bauch abheben
- Position 1 – 2 Sekunden halten

» **Effekt 1:**
- muskuläre Anspannung an Rücken, Gesäß und Oberschenkel nimmt zu

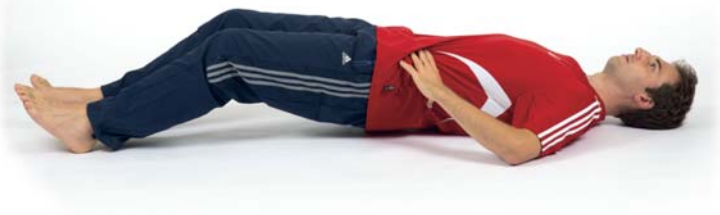

Aktion 2:
- Gesäß langsam bis kurz über Boden absenken

» **Effekt 2:**
- muskuläre Anspannung nimmt ab
- Lendenwirbelsäule hat wieder Kontakt zur Unterlage, das Gesäß nicht !

!

- Bei der Übung kein Hohlkreuz zulassen.
- Das Gesäß kann kurzzeitig abgelegt werden, wenn eine Pause erforderlich ist.
- Beide vordere Beckenpunkte befinden sich während der Übung auf gleicher Höhe. Kein einseitiges Absinken des Beckens zulassen – Kontrolle über Zeigefinger.

K 3	KRÄFTIGUNG HÜFTABSPREIZER
WIEDERHOLUNGSZAHL: 20	DURCHGÄNGE: 2

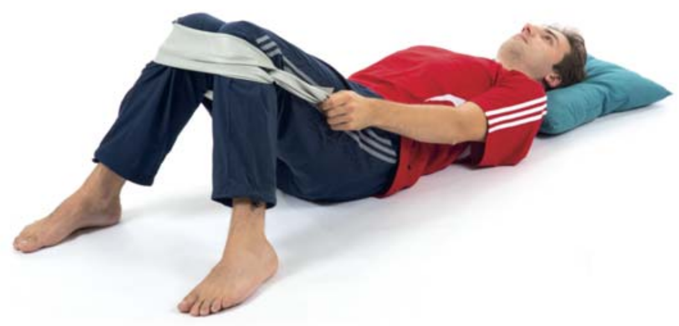

Ausgangsstellung: Rückenlage (Beine angestellt)

- seitlicher Fußabstand mindestens hüftbreit
- Knie gebeugt
- Band knapp unterhalb der Knie
- Abstand der Knie möglichst klein
- Lendenwirbelsäule hat Kontakt zu der Unterlage
- linke Hand hält Band
- rechte Hand an Oberschenkelaußenseite

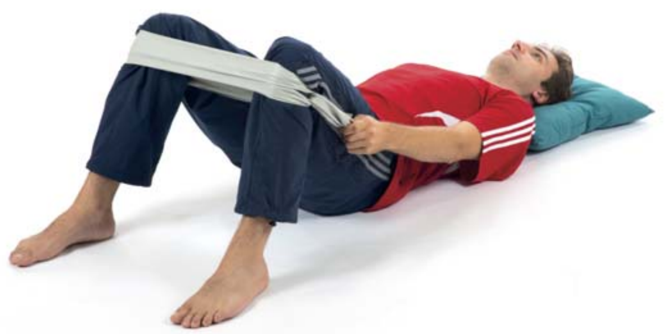

Aktion 1:
- beide Knie langsam nach außen führen, bis Abstand zwischen Knien dem Abstand der Füße entspricht
- Position 1 – 2 Sekunden halten

» **Effekt 1:**
- Bandspannung steigt
- muskuläre Anspannung Beinaußenseite nimmt zu

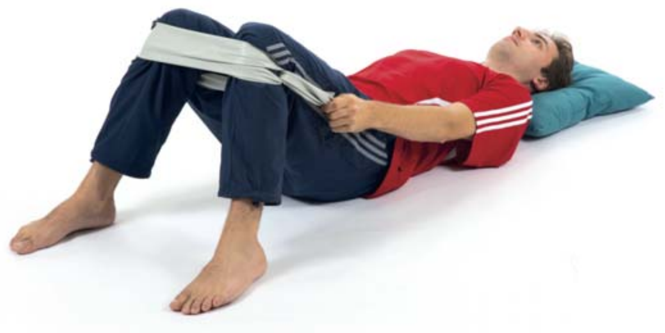

Aktion 2:
- langsam abbremsend beide Knie durch Zug des Bandes nach innen führen lassen

» **Effekt 2:**
- Bandspannung sinkt
- muskuläre Anspannung Beinaußenseite nimmt ab

!

- Bei Knieproblemen sollte das Band knapp oberhalb der Knie angelegt werden.

K 4	KRÄFTIGUNG HÜFTADDUKTOREN
WIEDERHOLUNGSZAHL: 20	DURCHGÄNGE: 2

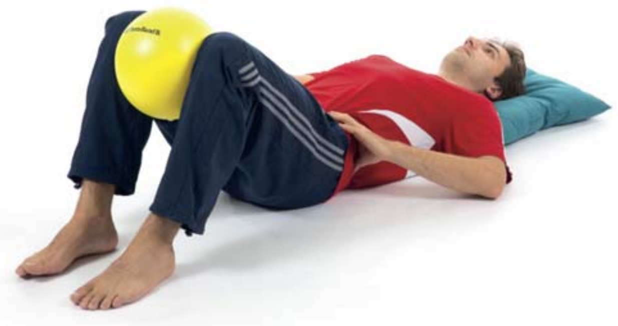

Ausgansstellung: Rückenlage (Bein angestellt)

- seitlicher Fußabstand 10 cm
- Knie gebeugt
- Ball hängt zwischen den Knien
- Lendenwirbelsäule hat Kontakt zu der Unterlage
- linke Hand auf Beckenkamm
- Zeigefinger auf vorderem Beckenpunkt
- rechte Hand auf Oberschenkelinnenseite

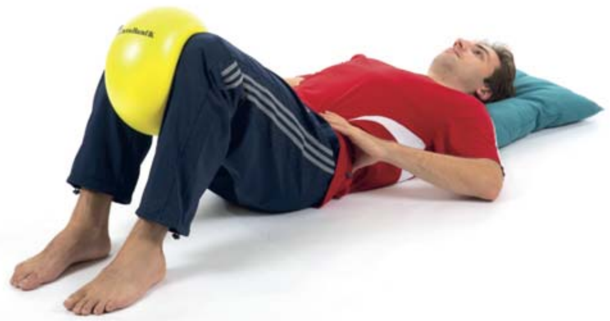

Aktion 1:
- beide Knie langsam und kräftig gegen Ball drücken
- Position 1-2 s halten

» **Effekt 1:**
- Druck auf Ball steigt
- muskuläre Anspannung Oberschenkelinnenseite nimmt zu

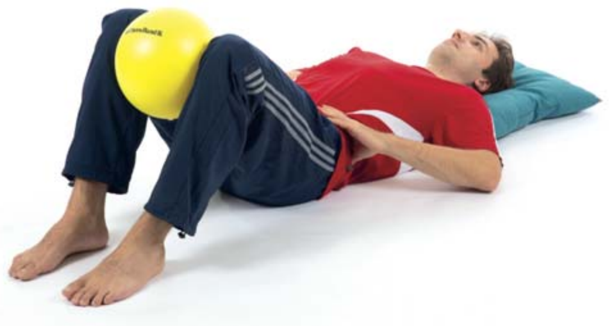

Aktion 2:
- beide Knie langsam wieder nach außen führen

» **Effekt 2:**
- Druck auf Ball sinkt
- muskuläre Anspannung Oberschenkelinnenseite nimmt ab

3.2.3 Phase III (Woche 9 – 12)

3.2.3.1 Übersicht

Auch in den letzten vier Wochen verläuft Ihr Training wie gewohnt. Erst Aufwärmen, dann das Gleichgewichtstraining und anschließend das Krafttraining.

Das Gleichgewichtstraining wird in der jeweils 2. Trainingseinheit durch Übungen zur Verbesserung der dynamischen Gleichgewichtsfähigkeit ergänzt. Dabei handelt es sich um 3 verschiedene Grundübungen mit je zwei Ausführungsvarianten (A + B). Sie absolvieren je einen Durchgang mit 6 Wiederholungen, in der von Ihnen gewählten Variante.

!

- **Zwischen den 2 Trainingseinheiten/Woche sollten mindestens 2 Tage Pause liegen.**
- **Die Übungen der beiden Trainingseinheiten unterscheiden sich grundsätzlich.**
- **Die Abfolge des Trainings bleibt jedoch in beiden Trainingseinheiten erhalten (Aufwärmen, Gleichgewichtstraining, Kräftigung).**

Kriterien für die Auswahl der Ausführungsvariante im dynamischen Gleichgewichtstraining:

- Wenn Sie die gewählte Ausführungsvariante ohne größere Anstrengung/Konzentration halten können, sollten Sie die Ausführungsvariante wählen, die Sie mehr fordert.
- Ist die Bewegung nur mit starken Ausgleichbewegungen möglich, wählen Sie bitte eine leichtere Variante.
  » Die Bewegung sollte ohne starke Ausgleichbewegungen der Arme und Schwankungen des Oberkörpers möglich sein. Zudem sollte die Bewegung rhythmisch und fließend sein und ein hohes Maß Ihrer Konzentration erfordern.

Das Krafttraining wechselt von der Kraftausdauermethode zum Muskelaufbautraining. Hierzu werden weniger Wiederholungen pro Durchgang bei deutlich höherer Trainingsintensität ausgeführt. (siehe Seite 39 – 41; 44).

Die Kräftigungsübungen erfolgen in der Trainingseinheit 1 wie bisher in der von Ihnen bevorzugten Ausgangsstellung. Sie werden in der Trainingseinheit 2 durch Übungen im Stand mit dem elastischen Band ergänzt. Falls es durch die Erhöhung der Belastungsintensität zu vermehrten Beschwerden kommt, finden Sie im Übungskatalog entsprechende Alternativen zu den jeweiligen Übungen.

Tabelle 10: Muskelgruppen der Hüfte – Ausgangsstellungen und Übungsnummern.

Muskulatur	Hüftstrecker	Hüftbeuger	Hüftabspreizer	Adduktoren
Kräftigungsübungen in Rückenlage	K 9	K 10a/b	K 11	K 12
Kräftigungsübungen im Stand und Sitz	K 13a/b	K 14a/b	K 15	K 16
Kräftigungsübungen im Stand	K 17	K 18	K 19	K 20

AUFGEPASST

WOCHE 9 – 12

1 2 3 4 5 6 7 8 **9 10 11 12**

ÜBUNGSMATERIALIEN

- Übungskatalog (Übungsanweisungen GG1 – 7, K 9 – 20 + Trainingstagebuch Woche 9 – 12)
- Lesezeichen
- Hilfsmittel: Band, Ball; Stabilisationstrainer; bei Bedarf Lagerungs- und Abstützhilfen

AUSGANGSSTELLUNG

Stand: GG1 – GG4 (Trainingseinheit 1)
Stand und Gang: GG5 – GG7 (Trainingseinheit 2)
Fließender sicherer Gang
Vermeidung von Ausgleichsbewegungen

Rückenlage: K9 – K12 (Trainingseinheit 1)

Stand und Sitz: K13 – K16 (Trainingseinheit 1)

Stand: K17 – K20 (Trainingseinheit 2)
Bei den Übungen zeigt der Fuß und das leicht gebeugte Knie des Standbeins nach vorne
Die Standposition kann durch Abstützen stabilisiert werden (siehe Seite 55)

ÜBUNGSINHALTE

Trainingseinheit 1
(1) **Gleichgewicht, statisch:** GG 1 – 4 wahlweise mit den Varianten A – D
(2) Kräftigung in Rückenlage und/oder Stand und Sitz: K9 – K16, alle Bewegungsrichtungen bei freier Wahl der Ausgangsstellung
Woche 9 + 10: 15 Wiederholungen, 3 Durchgänge (Pause zwischen den Durchgängen ca. 60 Sekunden)
Woche 11 + 12: 10 Wiederholungen, 4 Durchgänge (Pause zwischen den Durchgängen ca. 60 Sekunden)
Belastungsintensität veränderbar über Anlegen von Gewichtsmanschette (K 9 – K 10, K 13 – K 14) und Verlängerung des Hebels (K 9 b – K 10 b, K 13b – K 14b)

Trainingseinheit 2
(1) **Gleichgewicht, dynamisch:** GG 5 – GG 7 wahlweise mit den Varianten a oder b
(2) Kräftigung im Stand: K 17 – K 20, alle Bewegungsrichtungen im Stand mit Band
Woche 9 + 10: 15 Wiederholungen, 3 Durchgänge (Pause zwischen den Durchgängen ca. 60 Sekunden)
Woche 11 + 12: 10 Wiederholungen, 4 Durchgänge (Pause zwischen den Durchgängen ca. 60 Sekunden)
Belastungsintensität veränderbar über Bandanlage (Knie oder Sprunggelenk) und gewählter Bandstärke (Farbe) sowie Vorspannung des Bandes

WISSENSWERTES

Muskelaufbautraining .... Seite 39
Trainingsintensität .... Seite 39 – 41
Gleichgewicht dynamisch .... Seite 42 – 44
Dokumentation .... Seite 45
Schmerz .... Seite 46

GG 1 – 4 | TRAININGSEINHEIT 1 | **STATISCHES GLEICHGEWICHT**

WIEDERHOLUNGSZAHL: 6 | ÜBUNGSDAUER: POSITION 15 SEK. HALTEN

Ausführliche Beschreibung auf Seite 98 – 101

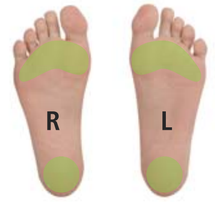

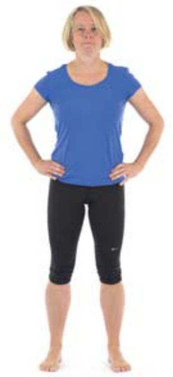

Ausgangsstellung:
Parallelstand

- seitlicher Fußabstand hüftbreit
- Knie leicht gebeugt
- Knie und Fußspitzen zeigen nach vorne
- Hände auf Beckenkamm
- Blick nach vorne

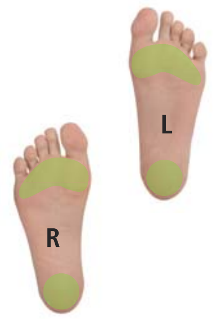

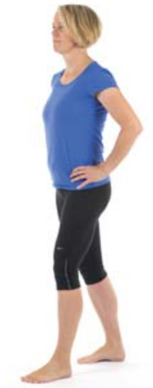

Ausgangsstellung: Schrittstellung

- linker Fuß steht vorne
- Füße hüftbreit hintereinander
- gleichmäßige Belastung der Füße
- Knie leicht gebeugt
- Knie und Fußspitzen zeigen nach vorne
- Hände auf Beckenkamm
- Blick nach vorne

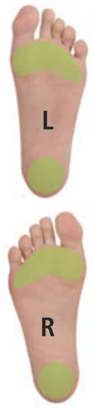

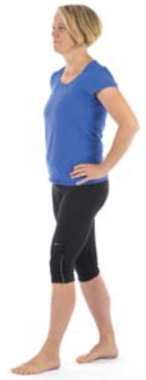

Ausgangsstellung: Tandemstand

- linker Fuß steht (wenn möglich) direkt vor rechtem Fuß
- gleichmäßige Belastung der Füße
- Knie leicht gebeugt
- Knie und Fußspitzen zeigen nach vorne
- Hände liegen auf Beckenkamm
- Blick nach vorne

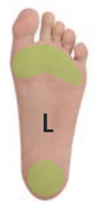

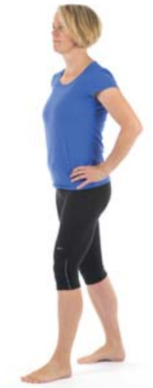

Ausgangsstellung: Einbeinstand

- Standbein links
- linkes Knie leicht gebeugt
- Knie und Fußspitzen zeigen nach vorne
- rechtes Bein 2 cm vom Boden abgehoben
- Hände auf Beckenkamm
- Blick nach vorne
- linkes und rechtes Bein berühren sich nicht

VARIANTEN A – D

Wählen Sie aus! Variante A, B, C oder D

A

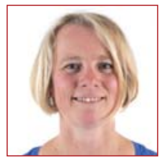

B

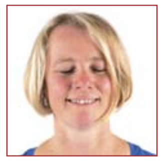

C

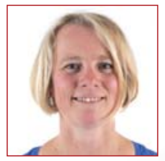

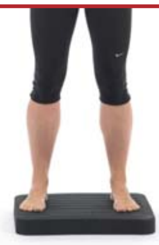

D

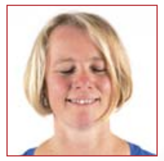

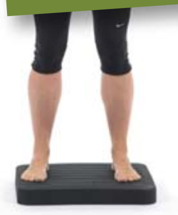

K 9 | TRAININGSEINHEIT 1 | **KRÄFTIGUNG HÜFTSTRECKER**

WOCHE 9+10 WIEDERHOLUNGSZAHL: 15 JE SEITE | DURCHGÄNGE: 3

WOCHE 11+12 WIEDERHOLUNGSZAHL: 10 JE SEITE | DURCHGÄNGE: 4

Wählen Sie aus! Übung K 9 oder K 13 a / b

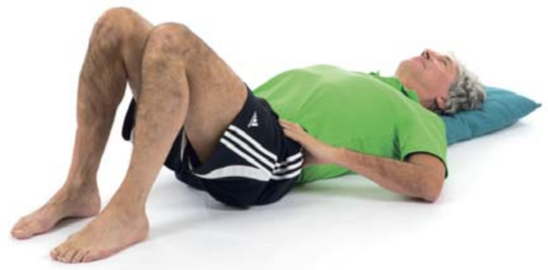

Ausgangsstellung:
Rückenlage (Beine angestellt)

- seitlicher Fußabstand hüftbreit
- Knie gebeugt
- Lendenwirbelsäule hat Kontakt zur Unterlage
- Hand auf Beckenkamm
- Zeigefinger auf vorderem Beckenpunkt

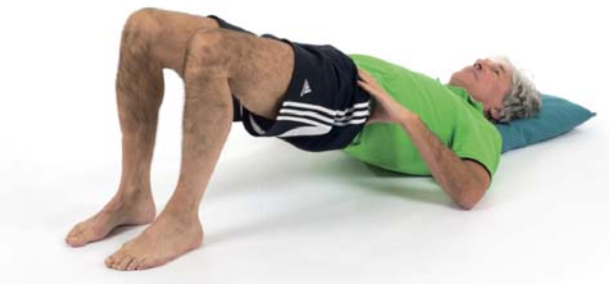

Aktion 1:
- Gesäß mit eingezogenem Bauch ohne Hohlkreuz abheben
- rechten Fuß kräftig gegen Boden drücken

» **Effekt 1:**
- muskuläre Anspannung an Rücken, Gesäß und rechtem Oberschenkel nimmt zu

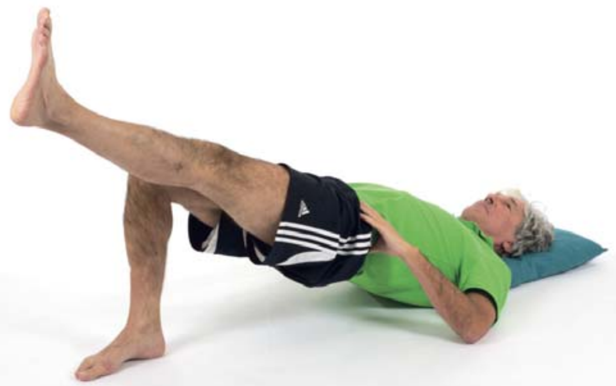

Aktion 2:
- linkes Bein abheben und strecken
- Position 1 – 2 Sekunden halten

» **Effekt 2:**
- muskuläre Anspannung an Rücken, Gesäß und rechtem Oberschenkel nimmt weiter zu

!

- Bei der Übung kein Hohlkreuz zulassen!
- Das Gesäß kann kurzzeitig abgelegt werden, wenn eine Pause erforderlich ist.
- Beide vordere Beckenpunkte befinden sich während der Übung auf gleicher Höhe. Kein einseitiges Absinken des Beckens zulassen – Kontrolle über Zeigefinger.
- **Alternative:** Wenn die Übung zu schmerzhaft oder zu schwer ist, können alternativ die Übungen K 1a oder K 1b ausgeführt werden.

K 13a | TRAININGSEINHEIT 1	KRÄFTIGUNG HÜFTSTRECKER (EINFACHE VARIANTE)
WOCHE 9+10 WIEDERHOLUNGSZAHL: 15 JE SEITE	DURCHGÄNGE: 3
WOCHE 11+12 WIEDERHOLUNGSZAHL: 10 JE SEITE	DURCHGÄNGE: 4

Wählen Sie aus! Übung K 9 oder K 13 a / b

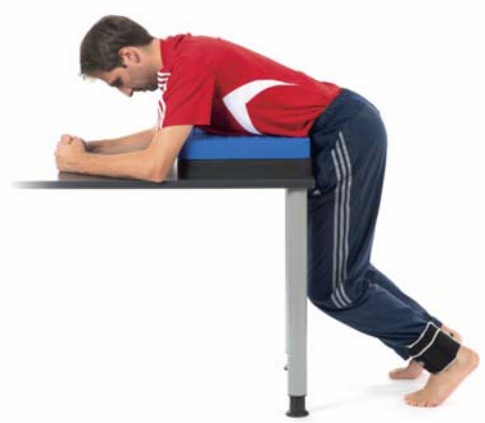

Ausgangsstellung: abgestützter Stand

- Gewichtsmanschette oberhalb der Knöchel anlegen
- seitlicher Fußabstand 10 cm
- Knie und Hüfte gebeugt
- Oberkörper liegt auf und wird über Arme abgestützt
- Leiste hat Kontakt zur Tischkante

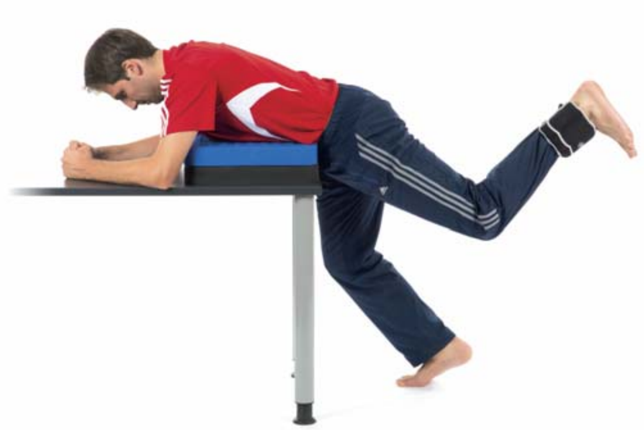

Aktion 1:
- linkes Knie anbeugen und langsam nach hinten oben führen (bis maximal in Verlängerung Oberkörper)
- Position 1 – 2 Sekunden halten

» **Effekt 1:**
- muskuläre Spannung Oberschenkelrückseite und Gesäß nimmt zu

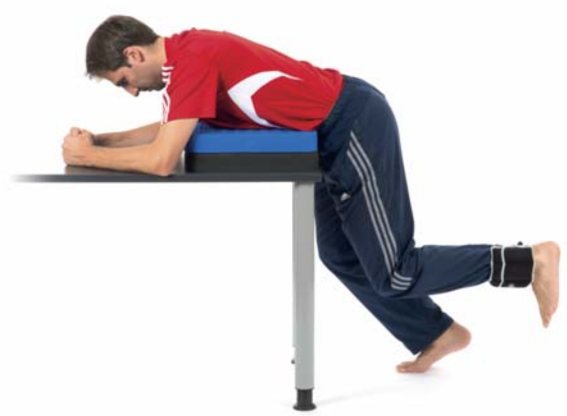

Aktion 2:
- linkes Bein langsam zur Ausgangsstellung absenken

» **Effekt 2:**
- muskuläre Spannung an Oberschenkelrückseite und Gesäß nimmt ab

!

- Über die gesamte Übungsdauer den Kontakt der Leiste und des Oberkörpers zum Tisch beibehalten.
- Während der Übung keine Beckenverdrehung oder Hohlkreuz zulassen.
- Bewegung nicht schwunghaft ausführen.
- Nach vorgegebener Wiederholungszahl Seite wechseln.
- **Alternative:** Wenn die Übung zu schmerzhaft oder zu schwer ist, können alternativ die Übungen K 5a oder K 5b ausgeführt werden.

K 13b | TRAININGSEINHEIT 1	KRÄFTIGUNG HÜFTSTRECKER (INTENSIVE VARIANTE)
WOCHE 9+10 WIEDERHOLUNGSZAHL: 15 JE SEITE	DURCHGÄNGE: 3
WOCHE 11+12 WIEDERHOLUNGSZAHL: 10 JE SEITE	DURCHGÄNGE: 4

Wählen Sie aus! Übung K 9 oder K 13 a / b

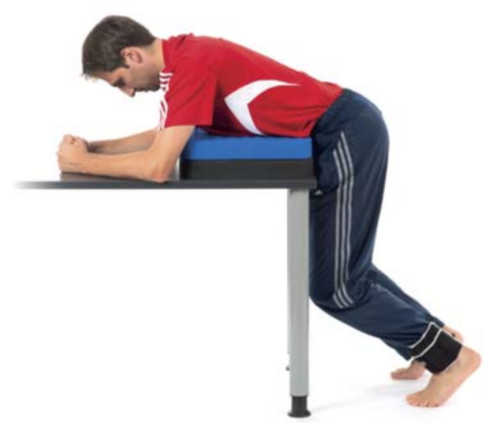

Ausgangsstellung: abgestützter Stand

- Gewichtsmanschette oberhalb der Knöchel anlegen
- seitlicher Fußabstand 10 cm
- Knie und Hüfte gebeugt
- Oberkörper liegt auf und wird über Arme abgestützt
- Leiste hat Kontakt zur Tischkante

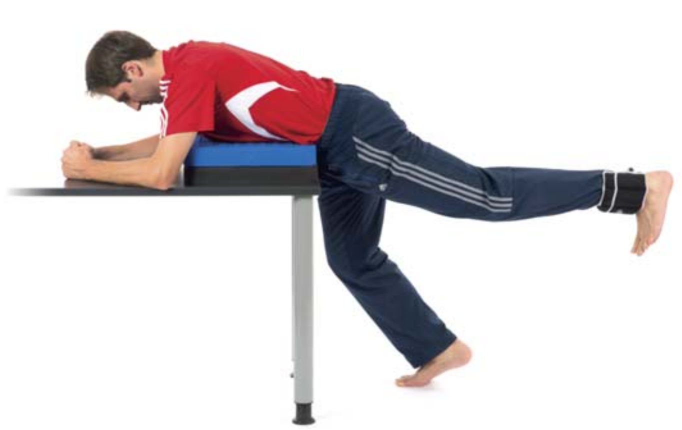

Aktion 1:
- linkes Knie anbeugen und langsam nach hinten oben führen, dabei das Knie zunehmend strecken
- Position 1 – 2 Sekunden halten

» **Effekt 1:**
- muskuläre Spannung Oberschenkelrückseite und Gesäß nimmt zu

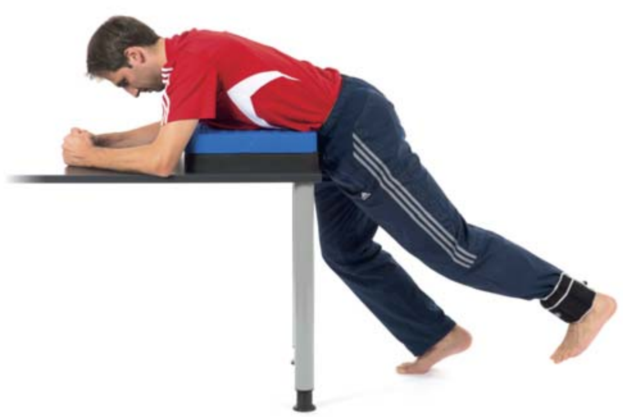

Aktion 2:
- linkes Bein langsam zur Ausgangsstellung absenken

» **Effekt 2:**
- muskuläre Spannung an Oberschenkel-rückseite und Gesäß nimmt ab

!

- Über die gesamte Übungsdauer den Kontakt der Leiste und des Oberkörpers zum Tisch beibehalten.
- Während der Übung keine Beckenverdrehung oder Hohlkreuz zulassen.
- Bewegung nicht schwunghaft ausführen.
- Nach vorgegebener Wiederholungszahl Seite wechseln.
- **Alternative:** Wenn die Übung zu schmerzhaft oder zu schwer ist, können alternativ die Übungen K 5a oder K 5b ausgeführt werden

K 12 | TRAININGSEINHEIT 1		KRÄFTIGUNG HÜFTADDUKTOREN
**WOCHE 9+10**	WIEDERHOLUNGSZAHL: 15 JE SEITE	DURCHGÄNGE: 3
**WOCHE 11+12**	WIEDERHOLUNGSZAHL: 10 JE SEITE	DURCHGÄNGE: 4

Wählen Sie aus! Übung K 12 oder K 16

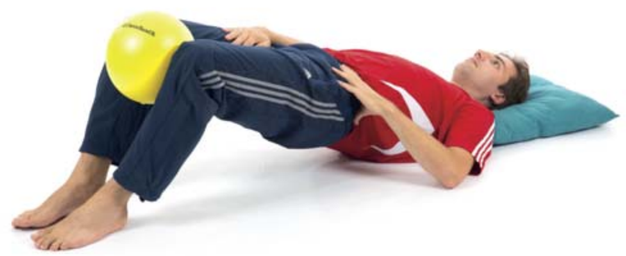

Ausgangsstellung:
Rückenlage

- seitlicher Fußabstand 10 cm
- Knie gebeugt
- Ball hängt zwischen den Knien
- Gesäß ist abgehoben
- Hand auf Oberschenkelinnenseite

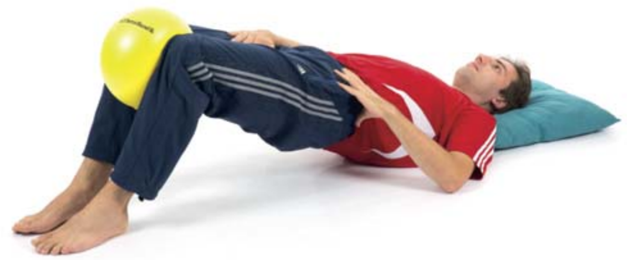

Aktion 1:
- beide Knie kräftig gegen den Ball drücken
- Position 1 – 2 Sekunden halten

» Effekt 1:
- Druck auf Ball steigt
- muskuläre Anspannung Oberschenkelinnenseite nimmt zu

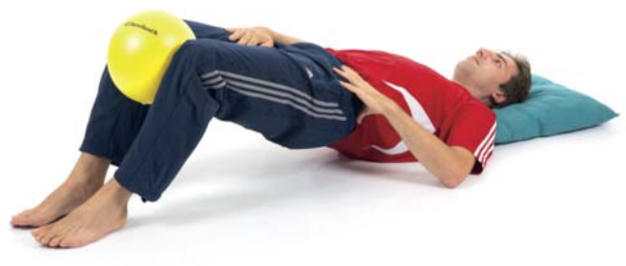

Aktion 2:
- beide Knie nach außen führen
- Gesäß bleibt oben

» Effekt 2:
- Druck auf Ball sinkt
- muskuläre Anspannung Oberschenkelinnenseite nimmt ab

!

- Während der Übung kein Hohlkreuz machen.
- Gesäß möglichst über die gesamte Wiederholungszahl oben halten.
  Falls nicht möglich, Gesäß zwischendurch ablegen
- **Alternative:** Wenn die Übung zu schmerzhaft oder zu schwer ist, kann alternativ die Übung K 4 ausgeführt werden.

K 16 | TRAININGSEINHEIT 1		KRÄFTIGUNG HÜFTADDUKTOREN
**WOCHE 9+10**	WIEDERHOLUNGSZAHL: 15 JE SEITE	DURCHGÄNGE: 3
**WOCHE 11+12**	WIEDERHOLUNGSZAHL: 10 JE SEITE	DURCHGÄNGE: 4

Wählen Sie aus! Übung K 12 oder K 16

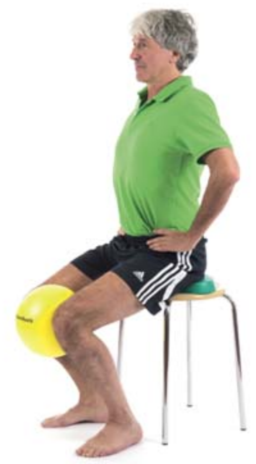

Ausgangsstellung: Sitz

- seitlicher Fußabstand 10 cm
- Füße etwas nach hinten versetzt
- Knie gebeugt und senkrecht über Fußspitzen
- Ball hängt zwischen den Knien
- Hände am Beckenkamm

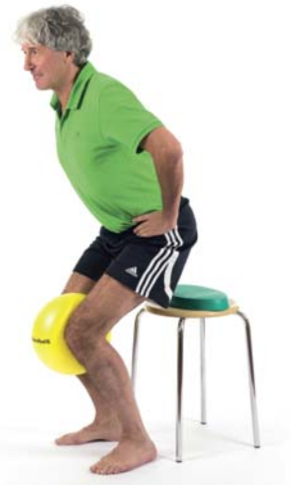

Aktion 1:
- Oberkörper nach vorne neigen
- Gesäß etwas abheben
- beide Knie langsam und kräftig gegen den Ball drücken
- Position 1 – 2 Sekunden halten

» Effekt 1:
- Druck auf Ball steigt
- muskuläre Anspannung Beine nimmt zu

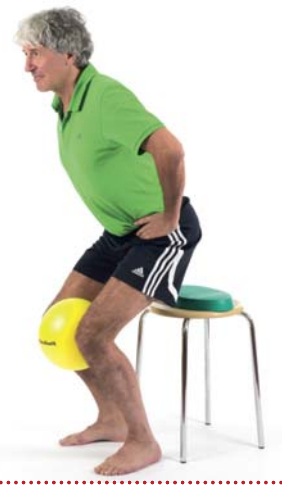

Aktion 2:
- beide Knie langsam nach außen führen
- Position von Oberkörper und Gesäß bleiben unverändert

» Effekt 2:
- Druck auf Ball sinkt
- muskuläre Anspannung an Beininnenseite nimmt etwas ab

!

- Das Gesäß möglichst über die gesamte Wiederholungszahl oben halten.
- Kein Hohlkreuz, kein Rundrücken zulassen!
- Bei Knieproblemen sollte die Übung in geringerer Kniebeugung durchgeführt werden.
- **Alternative:** Wenn die Übung zu schmerzhaft oder zu schwer ist, kann alternativ die Übung K 8 ausgeführt werden

GG 7A | TRAININGSEINHEIT 2

SCHRITT (EINFACHE VARIANTE)

WIEDERHOLUNGSZAHL: 6

DURCHGÄNGE: 1 JE SEITE

Wählen Sie aus! Übung GG 7a oder b

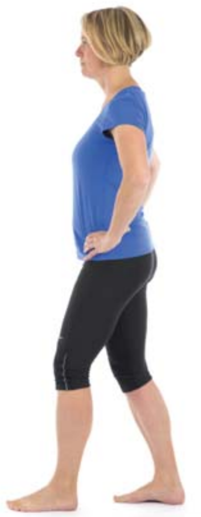

Ausgangsstellung: Schrittstellung, linker Fuß vorne

- seitlicher Fußabstand 5 cm
- Knie leicht gebeugt
- Knie und Füße zeigen nach vorne
- Füße gleichmäßig belastet
- Hände auf Beckenkamm

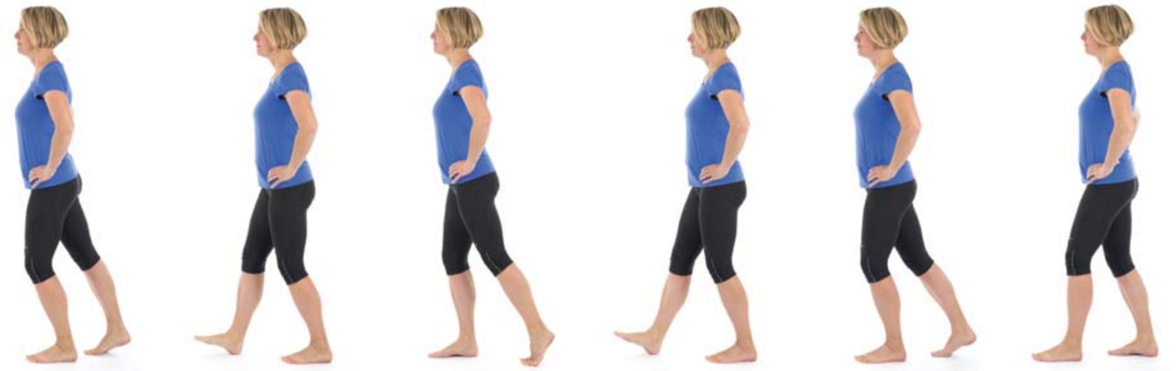

Aktion 1 Aktion 2 Aktion 3 Aktion 4 Aktion 5 Aktion 6

Aktion 1:
- Oberkörper, Becken und linkes Knie nach vorne verlagern

Aktion 2:
- rechtes Bein nach vorne bewegen und rechte Ferse aufsetzen

Aktion 3
- Oberkörper, Becken und rechtes Knie nach vorne verlagern
- Position 3 Sekunden halten

Aktion 4:
- Oberkörper und Becken nach hinten über linkes Bein verlagern

Aktion 5
- rechtes Bein nach hinten bewegen und hinter linkem Fuß aufsetzen

Aktion 6:
- Oberkörper und Becken nach hinten über rechtes Bein verlagern
- Position 3 Sekunden halten

» Effekt
- Standbein wechselt

!
- Führen sie die einzelnen Aktionen langsam und kontrolliert durch.
- Becken und Oberkörper bewegen sich ausschließlich nach vorne und hinten – nicht zur Seite.

GG 7B | TRAININGSEINHEIT 2

SCHRITT (SCHWIERIGE VARIANTE)

WIEDERHOLUNGSZAHL: 6

DURCHGÄNGE: 1 JE SEITE

Wählen Sie aus! Übung GG 7a oder b

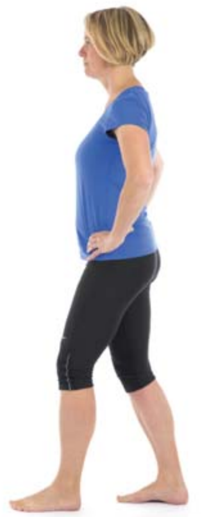

Ausgangsstellung: Schrittstellung, linker Fuß vorne

- seitlicher Fußabstand 5 cm
- Knie leicht gebeugt
- Knie und Füße zeigen nach vorne
- Füße gleichmäßig belastet
- Hände auf Beckenkamm

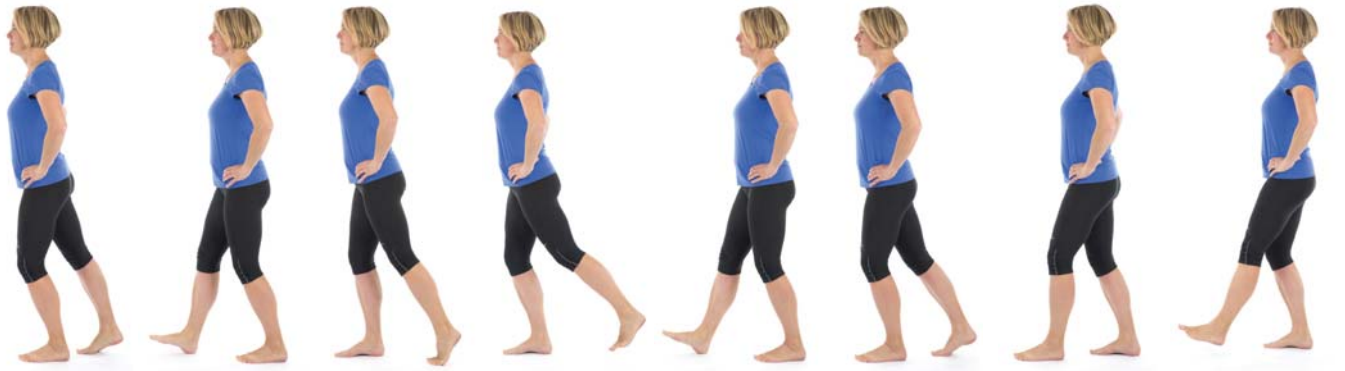

Aktion 1 Aktion 2 Aktion 3 Aktion 4 Aktion 5 Aktion 6

Aktion 1:
- Oberkörper, Becken und linkes Knie nach vorne verlagern

Aktion 2:
- rechtes Bein nach vorne bewegen und rechte Ferse aufsetzen

Aktion 3
- Oberkörper, Becken und rechtes Knie nach vorne verlagern
- linken Fuß etwa 5 cm vom Boden abheben
- Position 3 Sekunden halten

Aktion 4:
- linken Fuß wieder aufsetzen
- Oberkörper und Becken nach hinten über linkes Bein verlagern

Aktion 5
- rechtes Bein nach hinten bewegen und hinter linkem Fuß aufsetzen

Aktion 6:
- Oberkörper und Becken nach hinten über rechtes Bein verlagern
- linken Fuß etwa 5 cm vom Boden abheben
- Position 3 Sekunden halten

» Effekt
- Standbein wechselt

!
- Führen sie die einzelnen Aktionen langsam und kontrolliert durch.
- Becken und Oberkörper bewegen sich ausschließlich nach vorne und hinten – nicht zur Seite.
